# The effect of ‘*Candidatus* Liberibacter asiaticus’ infection on the proteomic profiles and nutritional status of pre-symptomatic and symptomatic grapefruit (*Citrus paradisi*) plants

**DOI:** 10.1186/1471-2229-13-59

**Published:** 2013-04-11

**Authors:** Chika C Nwugo, Hong Lin, Yongping Duan, Edwin L Civerolo

**Affiliations:** 1 San Joaquin valley Agricultural Sciences Center, USDA-ARS Parlier, California, 93648, USA; 2 USDA-ARS-USHRL, Fort Pierce, Florida, 34945, USA

**Keywords:** Grapefruit, Huanglongbing, Proteomics, Nutrients, Host response

## Abstract

**Background:**

Huanglongbing (HLB) is a highly destructive citrus disease which threatens citrus production worldwide and ‘*Candidatus* Liberibacter asiaticus’ (Las), a non-culturable phloem-limited bacterium, is an associated causal agent of the disease. To better understand the physiological and molecular processes involved in host responses to Las, 2-DE and mass spectrometry analyses, as well as ICP spectroscopy analysis were employed to elucidate the global protein expression profiles and nutrient concentrations in leaves of Las-infected grapefruit plants at pre-symptomatic or symptomatic stages for HLB.

**Results:**

This study identified 123 protein spots out of 191 spots that showed significant changes in the leaves of grapefruit plants in response to Las infection and all identified spots matched to 69 unique proteins/peptides. A down-regulation of 56 proteins including those associated with photosynthesis, protein synthesis, and metabolism was correlated with significant reductions in the concentrations of Ca, Mg, Fe, Zn, Mn, and Cu in leaves of grapefruit plants in response to Las infection, particularly in symptomatic plants. Oxygen-evolving enhancer (OEE) proteins, a PSI 9 kDa protein, and a Btf3-like protein were among a small group of proteins that were down-regulated in both pre-symptomatic and symptomatic plants in response to Las infection. Furthermore, a Las-mediated up-regulation of 13 grapefruit proteins was detected, which included Cu/Zn superoxide dismutase, chitinases, lectin-related proteins, miraculin-like proteins, peroxiredoxins and a CAP 160 protein. Interestingly, a Las-mediated up-regulation of granule-bound starch synthase was correlated with an increase in the K concentrations of pre-symptomatic and symptomatic plants.

**Conclusions:**

This study constitutes the first attempt to characterize the interrelationships between protein expression and nutritional status of Las-infected pre-symptomatic or symptomatic grapefruit plants and sheds light on the physiological and molecular mechanisms associated with HLB disease development.

## Background

Citrus Huanglongbing (HLB) or citrus greening disease is considered to be one of the most devastating diseases threatening citrus production worldwide, and all cultivated citrus species are susceptible or highly susceptible to the disease [1,2]. Orchard trees usually die in about 3–8 years after becoming symptomatic and yield losses of up to 65% have been reported [3]. Typically observed symptoms include asymmetric blotchy mottling and chlorosis of matured leaves, twig-dieback, small and misshapen fruits unsuitable for sale as fresh fruit or for juice; starch accumulation and phloem damage [1,2,4].

HLB was first reported in Asian countries in the 1870s [5]. Although Koch’s postulates are yet to be determined, the etiology of the disease has been associated with ‘*Candidatus* Liberibacter spp.’, a member of gram-negative, fastidious, phloem-limited α-proteobacteria. Taxonomically, there are three HLB-associated species namely, ‘*Candidatus* Liberibacter asiaticus’ (Las), ‘*Ca.* L. africanus’ and ‘*Ca.* L. americanus’ [1,2], which is based on their presumptive origins from the Asian, African and American continents, respectively, as well as distinctive 16S rDNA sequences. Among these three Liberibacter species, Las-associated HLB is the most prevalent and has been associated with increasing economic losses to citrus production worldwide [1,2]. Las is transmitted by and disseminated naturally by the Asian citrus psyllid (*Diaphorina citri*). In addition, there is a significantly extended latency period between times of infection and symptom development, which greatly complicates control strategies [2], making it crucial to develop fast, reliable and efficient methods for early detection of infected plants.

An important aspect of disease-associated plant-microbe interactions are the host responses induced at pre-symptomatic and symptomatic stages of disease development [6]. Identification of the host responses especially at the infection or pre-symptomatic stage can be critical towards understanding the initial processes involved in disease development and could be exploited in the formulation of efficient disease management practices [7-9]. High-throughput “omics” analyses provide fast, economical, efficient and holistic methods of understanding the molecular responses of biological systems to biotic and abiotic stress [10-12]. At least three separate but complementary transcriptomics studies using microarray technology have been performed to elucidate the effect of Las infection on the total mRNA expression levels in tissues of sweet orange (*Citrus sinensis*) plants [4,13,14]. However, differential gene expression at the transcriptional (mRNA) level do not necessarily correlate with differential gene expression at the translational (protein) level as posttranscriptional translational and/or posttranslational modifications; alternative splicing of mRNA transcripts; and mRNA stability and interference factors play important roles in regulating gene expression [15-18]. Proteins are the final products of gene expression and their expression levels directly correlate with cellular function. Thus, in order to fully understand the molecular mechanisms involved in the response of citrus plants to Las-infection, it is imperative to inquire beyond the transcriptional level and into the proteomic level of gene expression.

Furthermore, disease symptoms frequently reflect the altered nutritional status of plants and nutrient-disease interactions are well documented in plant systems [19]. A malfunctioning or blocked vascular system such as that implicated in HLB-disease development [4,13] can induce a systemic or localized nutrient sufficiency or deficiency. Physiological symptoms of HLB is suggested to resemble that of Zn-deficiency [20] and the productive life of diseased plants, including HLB-affected plants, has been shown to be extendable by fertilizer application [19,21]. Nutrient homeostasis forms part of a delicately balanced interdependent system with plant gene regulation; however, there is limited information on the relationships between the nutritional status and protein expression profiles of citrus plants during HLB development.

In this study we used a proteomic approach based on two-dimensional electrophoresis (2-DE) and mass spectrometry to characterize the comparative changes in the total leaf proteomes of Las-infected grapefruit plants that are pre-symptomatic or symptomatic for HLB. Inductively-coupled plasma (ICP) spectroscopy was also employed to resolve the nutrient concentrations in leaves of the same set of Las-infected leaf samples. Our results highlight molecular and physiological processes associated with HLB disease development.

## Results and discussion

### Effects of Las-infection on the leaf protein profile of pre-symptomatic and symptomatic grapefruit plants

**Figure 1:**
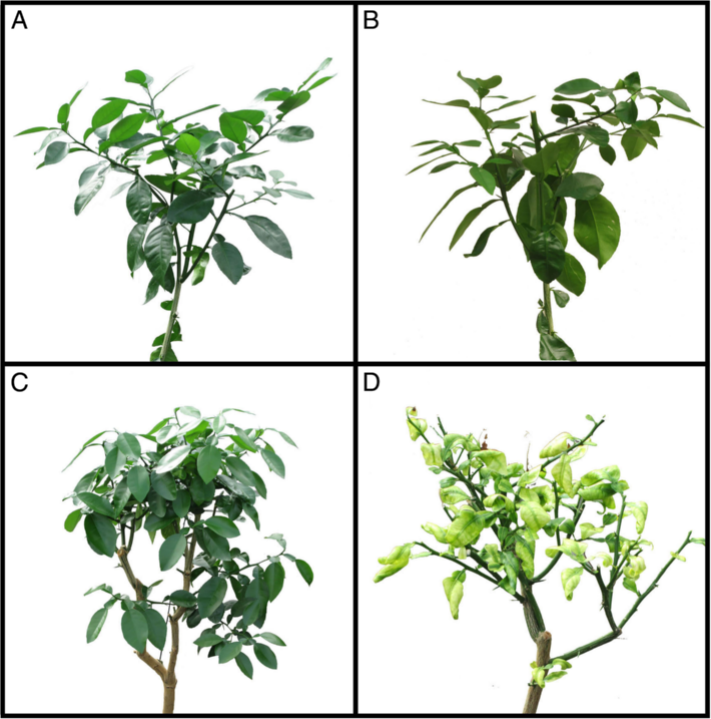
**Morphological characterization of grapefruit plants infected or uninfected with Las and pre-symptomatic or symptomatic for huanglongbing.** (**A**) Representative plant of uninfected control for pre-symptomatic (UP) plants; (**B**) Representative plant of infected pre-symptomatic (IP) plants; (**C**) Representative plant of uninfected control for symptomatic (US) plants; (**D**) Representative plant of infected symptomatic (IS) plants. Two-year old healthy plants were either graft-inoculated with side shoots from PCR-confirmed Las-infected bud sticks or uninoculated and leaf samples were analyzed at three months post-inoculation (for pre-symptomatic plants) or six months post-inoculation (for symptomatic plants).

**Figure 2:**
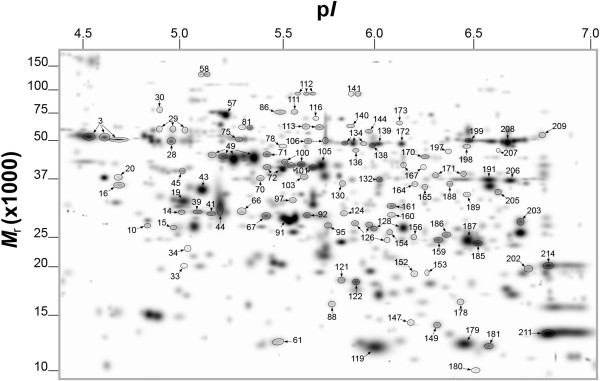
**PDQuest-generated master gel image showing the general pattern of matched protein spots from the total leaf proteome of grapefruit plants that were Las-infected or uninfected and pre-symptomatic or symptomatic for HLB.** Labeled protein spots were differentially produced in response to Las-infection and described in Tables 1, 2, and 3. Two-year old healthy plants were either graft-inoculated with side shoots from PCR-confirmed Las-infected bud sticks or uninoculated and leaf samples were analyzed at three months post-inoculation (for pre-symptomatic plants) or six months post-inoculation (for symptomatic plants). A total of 200 μg of protein was loaded on a pH 4–7 IpG strip and protein spots were visualized by staining with Coomassie Brilliant Blue (CBB). *M*_r_, relative molecular weight; p*I*, isoelectric point.

There was no visible difference in leaf morphology between the uninfected control for pre-symptomatic (UP) plants and the infected pre-symptomatic (IP) plants but the uninfected control for symptomatic (US) plants was visibly different from the infected symptomatic (IS) plants (Figure 1). A total protein yield of over 10 mg g^-1^ was extracted from leaves and there was no significant difference in the total protein yield across treatments (Additional file 1: Table S1). A high resolution 2-DE separation of total leaf proteins from grapefruit plants was visualized in a p*I* range of 4–7 and *M*_r_ range of 10,000 -150,000 (Additional file 2: Figure S1). Using PDQuest analysis software, over 700 spots per gel and over 440 reproducible spots within replicate gels were detected (Additional file 1: Table S1). Out of 191 differentially produced spots detected by PDQuest analysis, mass spectrometry analysis via MALDI-TOF- or LC-MS identified 123, which according to identical protein matches and spot proximity on the gel was summarized into 97 spots (Figure 2). A magnified view of the profiles of identified spots in representative gels from each treatment group is shown in Additional file 3: Figure S2.

In certain instances more than one spot was matched to a given protein, which as previously suggested, could be due to a combination of factors including multimerism/protein isoforms, maturation state, degradation and/or post-translational modifications [15,16]. Furthermore, although majority of the differential protein expression identified in this study was induced by Las infection, in certain instances, we observed a significant difference in protein expression between the uninfected control for pre-symptomatic (UP) plants and the uninfected control for symptomatic (US) plants (example see Table 1, spots 86 and 205). This could be due to developmental difference in leaf age between UP and US plants, which is supported by results from previous reports [22,23].

**Table 1:** Other identified proteins in citrus grapefruit leaves that were down-accumulated in response to Las-infection

**Spot**^**a**^	**ASV**^**b**^	**Protein function/name**^**c**^	**Accession #**^**c**^	**Theoretical**^**d**^		**S**^**e**^	**M**^**f**^	**E**^**g**^
	**UP IP US IS**			***M***_**r**_	**p*I***			
		***Energy/Metabolisms***						
121		Putative thioredoxin-dependent peroxidase	gi|119367465	17443	5.15	119	7	70
128		Ascorbate peroxidase 2	gi|221327589	27724	5.55	162	15	74
161		L-ascorbate peroxidase T, chloroplastic-like isoform 2	gi|359492510	42300	8.61	223	18	50
188		Coproporphyrinogen III oxidase, putative	gi|255554717	39324	7.66	162	17	47
189^h^		Isoflavone reductase related protein	gi|3243234	34281	5.92	54	4	19
205		Cinnamoyl-CoA reductase, putative	gi|255556687	35554	6.16	67	9	39
209		Catalase	gi|19070130	57669	6.64	110	13	35
		***Regulation/Protein synthesis***						
16		31 kDa ribonucleoprotein, chloroplastic	gi|225456840	38020	4.55	64	8	29
58		Elongation factor Ts	gi|357500731	82705	4.68	77	11	23
97		Proteasome subunit alpha type, putative	gi|255538698	29940	5.15	109	10	49
100		Glutamine synthetase plant, putative	gi|255551511	48172	6.29	183	21	68
105		Glutamine synthetase plant, putative	gi|255551511	48172	6.29	218	17	59
116		ATP-dependent zinc metalloprotease FTSH 2, chloroplastic-like	gi|225446693	75921	6.44	197	28	51
136		26S protease regulatory subunit 6b, putative	gi|255565346	44701	5.49	69	9	30
138		Mitochondrial processing peptidase alpha subunit, putative	gi|255546263	50379	5.91	163	20	58
149		Nucleoside diphosphate kinase 1	gi|19570344	16349	5.93	80	7	56
152^h^		Transcription factor homolog (Btf3-like) protein	gi|33945882	17821	5.93	61	3	35
165		Serine-type peptidase	gi|270342123	46267	8.24	128	8	30
170		S-adenosylmethionine synthetase, putative	gi|255548295	43620	5.65	217	26	71
178		Nucleoside diphosphate kinase, putative	gi|255540363	14819	6.92	68	4	35
185		DHAR class glutathione transferase DHAR2	gi|283135906	23962	6.18	110	8	45
198		Alanine aminotransferase 2 isoform 2	gi|359495900	54000	6.00	92	13	34
206		mRNA binding protein precursor	gi|350534514	43638	7.70	97	14	51
207		Alanine aminotransferase 2 isoform 2	gi|359495900	54000	6.00	188	24	57
		***Chaperones***						
10^h^		Putative FKBP-type peptidyl-prolyl cis-trans isomerase	gi|51471872	22889	4.78	46	6	35
30		Heat shock protein, putative	gi|255555659	73678	5.10	96	13	22
45		Peptidyl-prolyl cis-trans isomerase, putative	gi|255552604	48362	5.04	99	14	36
57		Chloroplast HSP70	gi|124245039	76759	5.31	168	22	35
81		Chaperonin-60alpha	gi|15226314	55491	4.86	210	22	60
86		Heat shock protein 70	gi|211906496	71346	5.10	118	22	47
111		70 kDa heat shock cognate protein 1	gi|45331281	71381	5.11	75	9	21
140		Chaperonin-60kD, ch60, putative	gi|255554262	56809	5.19	79	12	32
		***Unknown***						
144		Uncharacterized protein	gi|225443738	66158	8.50	78	7	20
197		Hypothetical protein VITISV_021486	gi|147834040	44041	5.98	68	7	24

^a^ The spot numbers correspond to the numbers given in Figure 2 and Additional file 3: Figure S2.

^b^ Average spot volume per treatment group; UP, uninfected reference for pre-symptomatic plants; IP, infected pre-symptomatic plants; US, uninfected reference for symptomatic plants; IS, infected symptomatic plants. Average spot volumes separated by letters to show significant difference are presented in Additional file 6: Appendix S1.

^c^ Protein function/name was determined by http://www.ncbi.nlm.nih.gov/BLAST/.

^d^ Theoretical relative molecular weight (*M*_r_) and isoelectric point (p*I*) were calculated by http://www.expasy.org/. Observed *M*_r_ and p*I* can be extrapolated from Figure 1.

^e^ Mascot score of protein hit.

^f^ Number of matched peptide masses. The sequences of PMF-matched peptides per spot are provided in Additional file 7: Appendix S2.

^g^ Percent sequence coverage of matched peptides.

^h^ Protein identification confirmed by MALDI-TOF-MS/MS. Sequencing information is provided in Additional file 7: Appendix S2.

^i^ Protein identification confirmed by LC-MS/MS.

**Figure 3:**
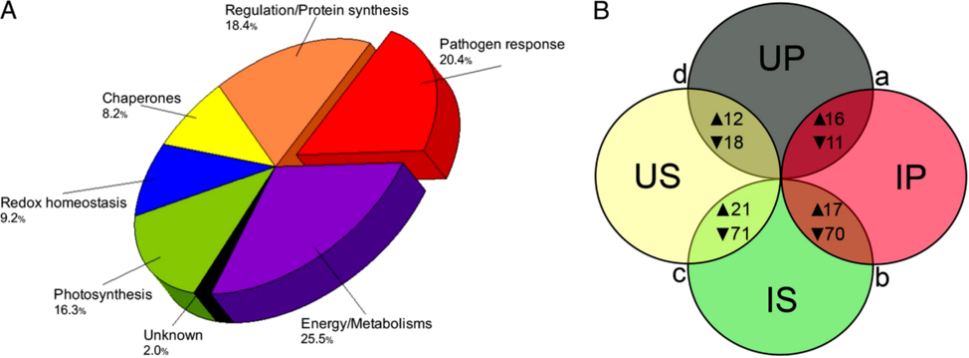
**Categorization of differentially produced proteins in grapefruit plants in response to Las-infection.** (**A**) Functional category distribution of all identified differentially produced protein spots from comparing 2-DE gel images of the total leaf proteome of grapefruit plants that were Las-infected or uninfected and symptomatic or pre-symptomatic for HLB. (**B**) Venn diagram with intersections a, b, c and d, showing the number of identified protein spots that were significantly up- (▲) or down- (▼) regulated in (**a**) infected pre-symptomatic (IP) plants compared to uninfected control for pre-symptomatic (UP) plants; (**b**) infected symptomatic (IS) plants compared to IP plants; (**c**) IS plants compared to uninfected control for symptomatic (US) plants; (**d**) US plants compared to UP plants.

**Figure 4:**
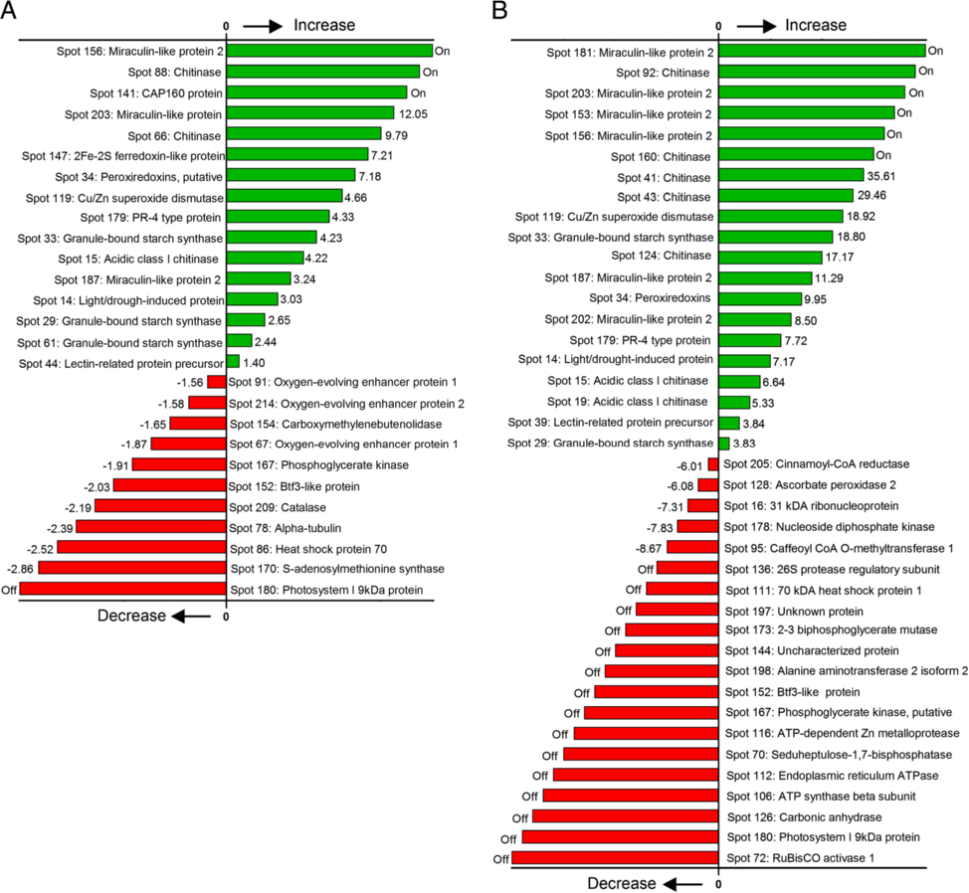
**A sorted categorization of differentially produced proteins in grapefruit plants in response to Las-infection.** (**A**) Sorting of the most increased or up-regulated proteins (green bars) to the most decreased or down-regulated proteins (red bars) in IP plants compared to UP plants. (**B**) Sorting of the 20 most increased proteins and 20 most decreased proteins in IS plants compared to US plants. The actual fold increase or decrease (−) is presented on the right or left side of green or red bars, respectively, and can also be extrapolated from Additional file 6 Appendix S1. Bars with “on” or “off” represent proteins that were “only detected” or “not detected”, respectively, in IP plants compared to UP plants or in IS plants compared to US plants. UP refer to uninfected control for pre-symptomatic plants. IP refers to infected pre-symptomatic plants. US refers to uninfected control for symptomatic plants. IS refers to infected symptomatic plants.

The differentially expressed 97 protein spots presented in Figure 2 matched to 69 proteins/peptides (Additional file 4: Table S2) and were broadly grouped into six categories according to putative physiological functions (Figure 3A) namely: (i) CO_2_ assimilation/photosynthesis-related (16.3%), (ii) redox homeostasis-related (9.2%), (iii) regulation/protein synthesis-related (18.4%), (iv) pathogen response-related (20.4%), (v) chaperones (8.2%), and (vi) energy/metabolisms-related (25.5%). Among the identified protein spots, the volumes of 27 spots significantly changed (16 up-accumulated and 11 down-accumulated) in infected pre-symptomatic (IP) plants compared to UP plants, while the volumes of 92 spots significantly changed (21 up-accumulated and 71 down-accumulated) in infected symptomatic (IS) plants compared to US plants (Figure 3B). Additionally, the volumes of 30 spots significantly changed (12 up-accumulated and 18 down-accumulated) in US plants compared to UP plants, while the volumes of 87 spots significantly changed (17 up-accumulated and 70 down-accumulated) in IS plants compared to IP plants (Figure 3B). The spots that were differentially produced according to treatment comparisons described in Figure 3B are presented in Additional file 5: Table S3. A ranking of the most increased or up-regulated to the most decreased or down-regulated protein in IP or IS plants compared to UP or US plants, respectively, shows that pathogen response-related proteins showed the most increase in fold change in response to Las infection while photosynthesis-related proteins showed the most decrease in response to Las infection (Figure 4). Average spot volumes separated by letters to show significant difference are presented in Additional file 6: Appendix S1, while the sequences of PMF-matched peptides per spot are provided in Additional file 7: Appendix S2.

### Effects of Las-infection on the nutrient status of pre-symptomatic and symptomatic grapefruit plants

**Figure 5:**
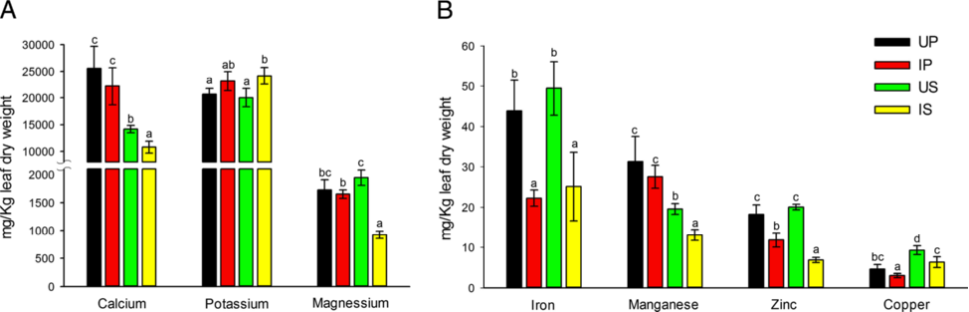
**The concentrations of macro- and micro-nutrients in leaves of grapefruit plants that were Las-infected or uninfected and pre-symptomatic or symptomatic for HLB.** (**A**) macronutrients calcium, potassium and magnesium; (**B**) micronutrients iron, manganese, zinc, and copper. UP, uninfected control for pre-symptomatic plants; IP, infected pre-symptomatic plants; US, uninfected control for symptomatic plants; IS, infected symptomatic plants. Two-year old healthy plants were either graft-inoculated with side shoots from PCR-confirmed Las-infected bud sticks or uninoculated and leaf samples were analyzed at three months post-inoculation (for pre-symptomatic plants) or six months post-inoculation (for symptomatic plants). Bars within an elemental group with the same lower case letter are not significantly different from each other (*P* > 0.05).

HLB symptoms are similar to those of Zn-deficiency [20] and the life expectancy of HLB-affected plants may be extendable by fertilizer application [21]. Additionally, plant nutrients are actively involved in gene regulation and several metabolically active proteins form co-enzymes with nutrients, which prompted our inquiry into the effects of HLB on the nutrient status of grapefruit plants in tandem with our proteomic analyses. In this study, Las infection was accompanied by a general reduction in the concentrations of Ca, Mg, Fe, Mn, Zn and Cu in leaves of grapefruit plants (Figure 5). However, the concentrations of Ca, Mg, and Mn were not significantly reduced in IP plants compared to UP plants but the concentrations of Fe, Zn and Cu showed significant 49%, 35% and 34% reductions, respectively, in IP plants compared to UP plants (Figure 5). This suggests an early inhibition of micronutrient availability in leaves of citrus plants during HLB disease development and is congruent with Zn-deficiency-like symptoms observed in HLB-affected plants [20]. Interestingly, the concentration of K was increased by 12% (*P* > 0.05) and 21% (*P* < 0.05) in IP and IS plants, respectively, compared to their corresponding control plants (Figure 5). Nutrient-disease interactions in plants have been well documented [19]. Low Ca in plant tissues was associated with susceptibility to macerating diseases caused by *Erwinia carotovora, Fusarium solani, Pythium myriotylum, Rhizoctonia solani, Sclerotinia minor, and Sclerotium rolfsii*[19]. Wheat tissues with higher Zn concentrations were shown to be less susceptible to spring blight [24]. Since the nutrients analyzed in this study are known to be directly involved in the regulation of plant metabolic processes, including protein expression, the potential consequences of Las-mediated effects on the nutrient status of grapefruit plants are further discussed below in relation with Las-mediated regulation of protein expression.

### CO_2_ Assimilation/photosynthesis

We observed a significant down-accumulation of ribulose-1, 5-bisphosphate carboxylase oxygenase (RuBisCO) (Table 2,spots 3, 28, 208 and 211), RuBisCO activase (Table 2, spots 49, 71, 72, and 101), carbonic anhydrase (Table 2, spots 126, 159), a photosystem (PS) II stability assembly factor HCF136 (Table 2, spot 164) in IS plants compared to US plants but not in IP plants compared to UP plants. This suggests that these proteins are not initially affected by Las-infection during early stage of HLB development. RuBisCO catalyzes the conversion of Ribulose-1, 5-bisphophate and inorganic CO_2_ to an unstable 6 carbon compound (3-keto-2-carboxyarabinitol-1, 5-bisphosphate) that disintegrates almost instantaneously into two molecules of glycerate-3-phosphate. Carbonic anhydrase catalyzes the rapid interconversion of carbon dioxide and water to bicarbonate and protons while RuBisCO activase catalyzes the rapid formation of the critical carbamate in the active site of RuBisCO.

**Table 2:** Photosynthesis- and Energy/Metabolisms-related citrus grapefruit leaf proteins that were down-accumulated in response to Las-infection

**Spot**^**a**^	**ASV**^**b**^	**Protein function/name**^**c**^	**Accession #**^**c**^	**Theoretical**^**d**^		**S**^**e**^	**M**^**f**^	**E**^**g**^
	**UP IP US IS**			***M***_**r**_	**p*I***			
		***CO***_***2 ***_***assimilation/Photosynthesis***						
3		Ribulose 1,5-bisphosphate carboxylase/oxygenase large subunit(chloroplast)	gi|114329664	53950	6.19	143	19	41
28		Ribulose 1,5-bisphosphate carboxylase/oxygenase large subunit(chloroplast)	gi|114329664	53950	6.19	94	14	36
49		Chloroplast ribulose-1,5-bisphosphate carboxylase/oxygenase activase large protein isoform	gi|115334977	41738	5.07	150	24	56
67		Oxygen evolving enhancer protein 1	gi|326467059	29262	5.32	133	14	67
71		Ribulose bisphosphate carboxylase/oxygenase activase 1, chloroplast precursor, putative	gi|255584538	51073	5.33	145	20	50
72		Ribulose bisphosphate carboxylase/oxygenase activase 1, chloroplast precursor, putative	gi|255584538	47200	5.94	144	21	55
91		Oxygen evolving enhancer protein 1	gi|326467059	29262	5.32	134	15	70
101		Chloroplast ribulose-1,5-bisphosphate carboxylase/oxygenase activase small protein isoform	gi|115334975	47200	5.94	148	26	63
113		Rubisco subunit binding-protein beta subunit, putative	gi|255564820	65086	5.85	211	25	50
126		Carbonic anhydrase, putative	gi|255568812	28499	5.51	135	13	56
159		Carbonic anhydrase 1	gi|30678350	28483	6.39	78	8	38
164		Photosystem II stability/assembly factor HCF136, chloroplast precursor, putative	gi|255559812	45094	8.46	147	10	38
180^h^		PSI 9 kDa protein	gi|224365649	9545	6.67	51	3	43
208		Ribulose 1,5-bisphosphate carboxylase/oxygenase large subunit (chloroplast)	gi|114329664	53950	6.19	106	17	41
211		Ribulose-1,5-bisphosphate carboxylase/oxygenase small subunit	gi|24940138	20521	9.16	92	8	41
214^h^		Oxygen-evolving enhancer protein 2, chloroplastic	gi|225446775	26777	8.63	48	6	32
		***Energy/Metabolisms***						
20		Protein grpE-like	gi|225439145	24749	4.61	67	8	47
70		Sedoheptulose-1,7-bisphosphatase, chloroplast, putative	gi|255579134	42768	5.82	67	15	31
75		Beta-tubulin	gi|223018283	50941	4.76	197	27	60
78		Alpha-tubulin	gi|134035496	42534	5.82	102	15	59
95		Caffeoyl CoA O-methyltransferase 1	gi|229368458	20972	5.39	74	8	57
103		Phosphoribulose kinase, putative	gi|255555933	45558	5.97	141	17	61
106		ATP synthase beta subunit, putative	gi|255582911	59862	6.06	152	22	51
112		Transitional endoplasmic reticulum ATPase, putative	gi|255556938	90244	5.14	265	27	47
122^h^		Bis(5'-adenosyl)-triphosphatase-like	gi|356539734	16962	6.07	48	4	40
130		Pyruvate dehydrogenase, putative	gi|255543140	39870	5.95	67	11	29
132		Alcohol dehydrogenase, putative	gi|255568816	41894	8.77	141	11	42
134		ATP synthase beta subunit, putative	gi|255582911	59862	6.06	278	27	63
139		2-phospho-D-glycerate hydrolase	gi|289600010	48059	5.54	142	15	53
154		Carboxymethylenebutenolidase, putative	gi|255567721	30236	7.10	74	7	37
167		Phosphoglycerate kinase, putative	gi|255544584	38338	9.37	108	10	38
171		Sorbitol dehydrogenase-like protein	gi|21553353	39923	6.33	119	11	36
172		2-phospho-D-glycerate hydrolase	gi|289600010	48059	5.54	235	20	64
173		Putative 2-3 biphosphoglycerate mutase	gi|239056191	61119	5.65	170	16	42
186		Triosphosphate isomerase-like protein type II	gi|262410515	27160	5.74	150	11	65
191		Malate dehydrogenase	gi|211906490	35859	6.10	196	19	66
199		Aldehyde dehydrogenase, putative	gi|255540719	52969	5.92	198	19	52

^a^ The spot numbers correspond to the numbers given in Figure 2 and Additional file 3: Figure S2.

^b^ Average spot volume per treatment group; UP, uninfected reference for pre-symptomatic plants; IP, infected pre-symptomatic plants; US, uninfected reference for symptomatic plants; IS, infected symptomatic plants. Average spot volumes separated by letters to show significant difference are presented in Additional file 6: Appendix S1.

^c^ Protein function/name and accession number was determined by http://www.ncbi.nlm.nih.gov/BLAST/.

^d^ Theoretical relative molecular weight (*M*_r_) and isoelectric point (p*I*) were calculated by http://www.expasy.org/. Observed *M*_r_ and p*I* can be extrapolated from Figure 1.

^e^ Mascot score of protein hit.

^f^ Number of matched peptide masses. The sequences of PMF-matched peptides per spot are provided in Additional file 7: Appendix S2.

^g^ Percent sequence coverage of matched peptides.

^h^ Protein identification confirmed by MALDI-TOF-MS/MS. Sequencing information is provided in Additional file 7: Appendix S2.

^i^ Protein identification confirmed by LC-MS/MS.

Our results agree with those of Albrecht and Bowman [13], which showed a significant Las-mediated reduction in the expression of photosynthesis-related genes transcripts such as chlorophyll A-B and photosystem II 5 kDa protein. Fan et al. [25] proposed that Las-induced inhibition of photosynthesis could be due to an accumulation of photosynthates such as sucrose and fructose, which might suppress photosynthetic activity via a negative feedback mechanism. Additionally, the general reduction in nutrient content due to Las infection can lead to a reduction in the production of “housekeeping” proteins as well as the cannibalization of proteins such as RuBisCO for nutrients [19]. During periods of biotic or abiotic stress the regulation of general protein production is usually skewed towards the production of stress-response related factors at the expense of “housekeeping” proteins [26]. The reducing energy generated in the light-dependent reactions of photosynthesis is also important in the reduction of sulfate and nitrate, which are necessary for protein biosynthesis and the Las-mediated inhibition of photosynthesis could play a role in the down-regulation of the expression levels of a diverse group of grapefruit leaf proteins (Table 2).

Furthermore, Las-infection was associated with a down-regulation of oxygen-evolving enhancer (OEE) proteins 1 and 2 (Table 2, spots 67, 91 and 214) as well as a PSI 9 kDa protein (Table 2, spot 180) in both IP and IS compared to the respective control plants. This suggests these proteins might be early targets during HLB disease development. OEE proteins 1 and 2 are subunits of the oxygen-evolving system of PSII and involved in stabilizing the Mn cluster [27]. However, unlike Fe, Zn and Cu, the elemental concentration of Mn was not significantly reduced in IP plants compared to UP plants (Figure 5), which suggests that a mechanism other than a canonical response to HLB-mediated reduction in nutrient availability might be associated with the early down-regulation of OEE proteins after Las-infection. Pathogens have been suggested to oxidize Mn from the reduced, plant available form, to the oxidized, non-available form as a virulence mechanism and isolates of *Gaeumannomyces graminis* and *Magnaporthe grisea* that cannot oxidize Mn have been shown to be avirulent [28]. HLB-affected trees generally show leaf yellowing (chlorosis) which is likely due to a reduction in chlorophyll biosynthesis [29,30] and Mg is important in chlorophyll biosynthesis. Thus, a Las-mediated reduction of the Mg content together with a reduction in Fe content of leaves of grapefruit plants (Figure 5) could play a role in HLB-associated chlorosis.

### Energy/metabolism

There was a general Las-mediated down-accumulation of energy production and metabolism-related proteins including ATP synthase beta subunit (Table 2, spots 106 and 134), sedoheptulose-1, 7-bisphosphatase (Table 2, spot 70), beta-tubulin (Table 2, spot 75), pyruvate dehydrogenase (Table 2, spot 130), alcohol dehydrogenase (Table 2, spot 132), and malate dehydrogenase (Table 2, spot 191) especially in IS plants compared to US plants. Interestingly, we observed a significant up-accumulation of granule-bound starch synthase (Table 3, spots 29, 33, 61) in IP and IS plants compared to the respective control plants. Several enzymes important in energy production and metabolism possess Fe-S clusters and the production of these proteins could be limited under reduced Fe availability as observed in this study (Figure 5). Additionally, Fe-S proteins act as Fe reservoirs in the cell and their degradation could be facilitated to release Fe [31].

**Table 3:** Citrus grapefruit leaf proteins that were up-accumulated in response to Las-infection

**Spot**^**a**^	**ASV**^**b**^	**Protein function/name**^**c**^	**Accession #**	**Theoretical**^**d**^		**S**^**e**^	**M**^**f**^	**E**^**g**^
	**UP IP US IS**			***M***_**r**_	**p*I***			
		***Energy/Metabolisms***						
29^i^		Granule-bound starch synthase	gi|223029784	67320	8.56			
33^i^		Granule-bound starch synthase	gi|223029784	67320	8.56			
61^i^		Granule-bound starch synthase	gi|223029784	67320	8.56			
		***Redox homeostasis***						
34^i^		Peroxiredoxins, prx-1, prx-2, prx-3, putative	gi|255578581	29299	8.38			
119^i^		Cu/Zn superoxide dismutase	gi|2274917	12784	5.82			
147^i^		2Fe-2S ferredoxin-like protein	gi|18397961	17602	7.75			
		***Pathogen response***						
14^i^		Chloroplastic light/drought-induced stress protein	gi|22261807	35216	5.24			
15^i^		Acidic class I chitinase	gi|23496445	34123	4.70			
19		Acidic class I chitinase	gi|23496445	36735	4.81	64	8	24
39		Lectin-related protein precursor	gi|11596188	29272	5.10	72	7	32
41^h^		Chitinase	gi|1220144	32459	5.06	53	5	20
43		Chitinase	gi|1220144	36735	4.81	73	11	41
44		Lectin-related protein precursor	gi|11596188	29272	5.10	85	7	32
66^i^		Chitinase	gi|1220144	31909	5.06			
88^i^		Chitinase	gi|1220144	31909	5.06			
92^i^		Chitinase	gi|1220144	31909	5.06			
124		Chitinase	gi|1220144	36735	4.81	110	11	45
141^i^		CAP160 protein	gi|22327778	65970	5.07			
153		Putative miraculin-like protein 2	gi|119367468	17806	6.74	105	7	49
156^i^		Miraculin-like protein 2	gi|11596180	25652	6.10			
160^i^		Chitinase	gi|1220144	31909	5.06			
179^i^		PR-4 type protein	gi|3511147	15227	5.50			
181^i^		Miraculin-like protein 2	gi|87299377	24120	5.61			
187^i^		Miraculin-like protein 2	gi|87299377	24120	5.61			
202		Putative miraculin-like protein 2	gi|119367468	23610	8.18	126	9	54
203^i^		Miraculin-like protein 2	gi|87299377	24120	5.61			

^a^ The spot numbers correspond to the numbers given in Figure 2 and Additional file 3: Figure S2.

^b^ Average spot volume per treatment group; UP, uninfected reference for pre-symptomatic plants; IP, infected pre-symptomatic plants; US, uninfected reference for symptomatic plants; IS, infected symptomatic plants. Average spot volumes separated by letters to show significant difference are presented in Additional file 6: Appendix S1.

^c^ Protein function/name was determined by http://www.ncbi.nlm.nih.gov/BLAST/.

^d^ Theoretical relative molecular weight (*M*_r_) and isoelectric point (p*I*) were calculated by http://www.expasy.org/. Observed *M*_r_ and p*I* can be extrapolated from Figure 1.

^e^ Mascot score of protein hit.

^f^ Number of matched peptide masses. The sequences of PMF-matched peptides per spot are provided in Additional file 7: Appendix S2.

^g^ Percent sequence coverage of matched peptides.

^h^ Protein identification confirmed by MALDI-TOF-MS/MS. Sequencing information is provided in Additional file 7: Appendix S2.

^i^ Protein identification confirmed by LC-MS/MS.

The accumulation of starch in plant tissues during HLB disease development has been previously demonstrated [25,32,33] and we earlier discussed our observation of a Las-mediated down-regulation of photosynthesis-related proteins. In plants, the surplus carbohydrates (sugars) produced during photosynthesis is stored as starch. Thus, an HLB-mediated inhibition of downstream metabolic pathways could contribute to starch accumulation in citrus plants and starch accumulation could result in an inhibition of photosynthesis via a negative feed-back mechanism. The transcriptomic studies by Albrecht and Bowman [13] and by Fan et al. [34] showed a similar Las-mediated inverse relationship between the expression of gene transcripts involved in starch anabolism with those associated with photosynthesis in citrus plants. However, a similar study by Kim et al. [4] only demonstrated a Las-mediated up-regulation of starch-anabolism-related gene transcripts and no significant effect on photosynthesis-related gene transcripts in HLB-affected sweet orange plants. Furthermore, a proteomic study by Fan et al. [35] failed to identify a Las-mediated effect on starch anabolism- or photosynthesis-related proteins in HLB-affected sweet orange plants. Thus, our present study is the first to simultaneously identify the proteomic mechanisms potentially involved in Las-mediated up-regulation of starch accumulation when accompanied by a down-regulation of photosynthesis in HLB-affected citrus plants.

Additionally, while the major HLB-induced starch anabolism-related gene transcript detected by Albrecht and Bowman [13] and Kim et al. [4] were those coding for the large subunit of ADP-glucose pyrophosphorylase (ADPase), the major HLB-induced starch anabolism-related protein detected in our present study was a granule-bound starch synthase. Starch is composed of two distinct polymers: amylopectin and amylose. Amylopectin consists of long chains of (1, 4)-linked α-D-glucopyranosyl units with extensive branching resulting from (1–6) linkages, while amylose is a relatively linear molecule of (1, 4)-linked α-D-glucopyranosyl units [36]. Starch biosynthesis is controlled by four major enzymes namely: ADPase, starch synthase, granule-bound starch synthase, and starch debranching enzyme. ADPase is the rate-limiting enzyme, and catalyzes the ATP-dependent interconversion of glucose-1-phosphate to ADP-glucose. ADP-glucose is then polymerized into amylopectin by multiple isoforms of starch synthase or to amylose by granule-bound starch synthase [37]. The extensive branching of amylopectin is the result of the balanced activities of starch-branching and -debranching enzymes. Thus, the result from our present study suggests that of the four starch biosynthesis-associated enzymes, granule-bound starch synthase is the most post-transcriptionally up-regulated protein during the early stage of Las infection in grapefruit.

Furthermore, granule-bound starch synthase requires K for activation [38] which might explain our observation of a 12% (*P* > 0.05) and 21% (*P* < 0.05) increase in IP and IS plants, respectively, compared to the respective control plants (Figure 5). Pathogen-mediated disruption of membrane permeability could lead to electrolyte leakage [19], which could be a virulence mechanism by Las to induce host K accumulation in order to sustain increased starch accumulation, thus providing a steady source of carbon and other nutrition for this phloem-limited pathogen while the host cells are deprived of these nutrients. This hypothesis is consistent with our observation of Las-mediated up-accumulation of lectin-like proteins (discussed later). The disruption of K balance could also lead to guard cell malfunction and arrest of transpiration limiting nutrient uptake as well as CO_2_ assimilation/photosynthesis [39]. To the best of our knowledge this study is the first to show a direct relationship between the accumulation of K nutrient and accumulation of granule-bound starch synthase in citrus leaves in response to Las infection, which might suggest a signature pathophysiological response of citrus plants to Las infection.

### Pathogen response

We showed that Las-infection resulted in an up-regulation of a PR-4 type protein (Table 3, spot 179), chitinases (Table 3, spots 41, 43, 66, 88, 92, 124, and 160), lectin-related proteins (Table 3, spots 39 and 44), miraculin-like proteins (Table 3, spots 153, 156, 181, 187, 202, and 203), and a chloroplastic light/drought-induced stress protein (Table 3, spot 14) in IP and IS plants compared to their respective control plants. We also identified a CAP 160 protein (Table 3, spot 141) which was up-accumulated in IP plants compared to UP plants but was undetected in US or IS plants. These observations are generally consistent with those from previous reports [14,35]. However, our identification of a Las-mediated up-regulation of a CAP 160 protein and a chloroplastic light/drought-induced stress protein, which are uncharacterized proteins, is novel.

The function of CAP160 proteins in plants is obscure but the protein has been associated with drought-, desiccation- and cold-stress tolerance [40]. Thus, our observation of an early Las-mediated up-regulation of a CAP 160 protein and a chloroplastic light/drought-induced stress protein suggest attempts by the host plant to mitigate a possible disruption of the water balance by Las via a disrupted transpiration stream as earlier postulated.

Pathogenesis-related (PR) proteins are plant proteins that are induced in response to pathogen attack. However, several studies suggest that these proteins can also be induced by a variety of abiotic stresses, such as wounding and exposure to chemicals or heavy metals [26,41-43]. The PR-4 family of PR proteins consists of class I and class II chitinases, which differ by the presence (class I) or absence (class II) of a conserved N-terminal cysteine-rich domain corresponding to the mature hevein, a small antifungal protein isolated from rubber tree (*Hevea brasiliensis*) latex [44].

Lectin-like proteins are involved in vascular tissue differentiation [45] and are associated with the plugging of phloem sieve plates in response to wounding and defense against pathogens and insects [46]. Accumulation of Phloem protein 2 (PP2), a lectin-like protein, at the sieve plates together with phloem necrosis and blockage of the translocation stream was demonstrated by Kim et al. [4] and Achor et al. [47] in HLB-affected citrus plants. Furthermore, the deposition of PP2 with callose at the sieve plates played a role in the recovery of apple trees from apple proliferation disease caused by the phloem-limited pathogen ‘*Candidatus* Phytoplasma mali’ [48]. Las, a phloem-limited bacterium, might induce the production of lectin-related proteins in host plants in order to inhibit phloem flow and accumulate photosynthates to nourish further bacterial growth as previously suggested. On the other hand host plants might induce the production of lectin-like proteins as a defensive attempt to prevent the spread of Las by sealing off the sieve tubes. Additionally studies have demonstrated that lectin-like proteins are able to interact with RNA molecules, are involved in the long-distance trafficking of macromolecules and may play a role in long-distance signaling in response to infection by plant pathogens [49,50].

In agreement with our results, a proteomics study by Fan et al. [35] also showed a Las-mediated up-regulation of miraculin-like proteins and gene transcripts in sweet orange plants. Recently, two distinct miraculin-like proteins, RlemMLP1 and RlemMLP2, were characterized in rough lemon (*Citrus jambhiri* Lush), and shown to have protease inhibitor activities as well as being involved in defense against pathogens [51]. During the development of citrus sudden death (CSD) disease, a miraculin-like protein was suppressed in susceptible plants but not in tolerant plants [52]. Increased levels of PR proteins as well as miraculin-like proteins was observed in leaves of *C. clementina* plants after infestation by the spider mite *Tetranychus urticae* or exposure to methyl jasmonate [53].

Taken together, 20.4% of the differentially expressed protein spots identified in this study matched to pathogen response-related proteins (Figure 3A), which were all up-regulated in grapefruit plants in response to Las infection (Table 3). However, the concerted up-regulation of these pathogen response-related proteins was evidently not sufficient to hinder HLB development. Thus, the development/engineering of citrus plants that constitutively express these stress-response related proteins could help reduce susceptibility to Las infection [14]. Additionally, the production of these proteins in pre-symptomatic plants could be exploited to develop novel/improved serological diagnostic methods for early identification of HLB-affected plants.

### Redox homeostasis

Redox-homeostasis-related proteins are usually involved in the prevention of oxidative stress, which is induced by reactive oxygen species (ROS). ROS are by-products of electron transport and redox reactions from metabolic processes such as photosynthesis and respiration. The production of ROS is markedly increased under conditions of biotic or abiotic stress [54,55]. We observed that Las-infection up-regulated the production of peroxiredoxins (Table 3, spot 34) and Cu/Zn superoxide dismutase (Table 3, spot 119) in IP and IS plants compared to their respective control plants. Additionally, we observed a Las-mediated up-regulation of a 2Fe-2S ferredoxin-like protein particularly in IP plants compared to UP plants.

Antioxidants, such as superoxide dismutase (SOD), are among the most potent in nature in protecting living systems against oxidative stress. While the role of Cu/Zn SOD in HLB disease development in citrus plants has been previously demonstrated [14,35], this study provides novel evidence for the potential involvement of two other redox homeostasis-related proteins: peroxiredoxins and an uncharacterized 2Fe-2S ferredoxin-like protein. ROS are produced sequentially: superoxide (O_2_ ¯) is the first reduction product of ground state oxygen and it can undergo spontaneous or SOD-catalyzed dismutation to H_2_O_2_, which is the second reactive product. Although, H_2_O_2_ is less reactive than superoxide, it is very diffusible and directly inactivates key cellular processes. Peroxiredoxins are a family of thiol-based peroxidases which catalyze the detoxification of H_2_O_2_ and other peroxides within living systems [56,57]. Interestingly, ascorbate peroxidase (Table 1, spots 128 and 161) and catalase (Table 1, spot 209), which are very important components of H_2_O_2_ detoxification [58], were down-regulated in response to Las-infection suggesting that peroxiredoxins might be preferentially recruited to help dissipate oxidative stress during HLB disease development.

Besides ascorbate peroxidase and catalase, we also observed that Las-infection resulted in a significant down-regulation of other redox homeostasis-related proteins such as a putative thioredoxin (Table 1, spot 121), coproporphyrinogen III oxidase (Table 1, spot 188), isoflavone reductase (Table 1, spot 189), and cinnamoyl-CoA reductase (Table 1, spot 205) particularly in IS plants compared to US plants. The reduction in antioxidative protein production could be a consequence of Las-mediated reduction in the plant nutrient content (Figure 5). Rellán-Álvarez et al. [59] showed a significant down-regulation in the production of antioxidants in *Beta vulgaris* due to Fe-deficiency. Albrecht and Bowman [14] suggested that their observed Las-mediated up-regulation of several gene transcripts for a Zn transporter 5 precursor (ZIP5) in ‘Cleopatra’ mandarin leaves was likely an attempt of the host to increase the uptake of Zn to potentially support an up-regulation of Cu/Zn SOD in response to HLB-induced deficiency. Furthermore, Albrecht and Bowman [14] identified 326 genes which were significantly up-regulated in a Las-susceptible citrus genotype compared to 17 genes which were significantly up-regulated in a Las-tolerant citrus hybrid. They also showed a significant over-expression of 559 transcripts including several with stress-response related functions such as oxidoreductase, Cu/Zn SOD and isoflavone reductase in the leaves of the Las-tolerant citrus hybrid compared with leaves of the Las-susceptible citrus genotype irrespective of Las-infection. We therefore suggest that the susceptibility of grapefruit plants to HLB might be associated with the inability of the plants to constitutively express their repertoire of antioxidants to mitigate the debilitating effects of Las-mediated ROS production.

### Regulation/Protein synthesis

Considering the general physiological decline that accompanies HLB development, it is not surprising that proteins associated with regulation/protein synthesis including such as a 31 kDa ribonucleoprotein (Table 1, spot 16), EF-Tu (Table 1, spot 58), glutamine synthetase (Table 1, spots 100 and 105), an ATP-dependent zinc metalloprotease (Table 1, spot 116), a serine-type peptidase (Table 1, spot 165), nucleoside diphosphate kinase 1 (Table 1, spot 149), and alanine aminotransferase 2 (Table 1, spot 198), were markedly repressed by Las especially in IS plants compared to US plants. Interestingly, we observed a significant Las-mediated down-regulation of a transcription factor homolog (Btf3-like) protein (Table 1, spot 152) and S-adenolsyl-L-methionine synthetase (Table 1, spot 170) in IP and IS plants compared to control plants, suggesting that these proteins might be early targets of Las pathogenesis or an early susceptibility response by grapefruit during HLB development.

The RNA polymerase B transcriptional factor 3 (Btf3) was demonstrated to be associated with apoptosis in mammalian cells [44,60] but its actual function in plants is not well understood. However, recently, Huh et al. [61] showed that silencing of Btf3 protein expression in *Capsicum annuum* and *Nicotiana benthamiana* plants led to reduced hypersensitive response (HR) cell death and decreased expression of some HR-associated genes. HR cell death upon pathogen infection has been described as a strategy devised by plants for inhibiting pathogen spread and obtaining systemic acquired resistance against further infection [62,63]. Thus, an early Las-mediated reduction in the production of a Btf3-like protein in grapefruit plants would have facilitated the spread of the bacterium within the host.

S-adenosylmethionine synthetase (SAMS) catalyzes the formation of S-adenosylmethionine (AdoMet) from methionine and ATP. AdoMet is an important methyl group donor utilized in most transmethylation reactions, which play vital roles in the synthesis of lipids, nucleic acids, proteins, and other products of secondary metabolism. Significant reductions in SAMS abundance in plants has been associated with abiotic stress particularly salt stress [64,65], and Hua et al. [66] showed that the expression of a *Glycine soja* SAMS gene in alfalfa (*Medicago sativa* L) plants enhanced alfalfa’s tolerance to salt stress. Thus, the Las-mediated increase in the concentration of K in IP plants compared to UP plants (Figure 5) could induce osmotic stress similar to that caused by salt stress and ultimately resulting in an early Las-mediated down-regulation of SAMS.

### Chaperones

Molecular chaperones [e.g. heat shock proteins (HSPs), chaperonins and peptidyl-prolyl cis-trans isomerases] are proteins involved in protein folding, refolding, assembly, re-assembly, degradation and translocation [67-70]. It is, therefore, not surprising that the broad Las-mediated down-regulation of proteins associated with regulation/protein synthesis was accompanied by a corresponding down-regulation in the expression levels of chaperones including peptidyl-prolyl cis-trans isomerase (Table 1, spot 45), heat shock proteins (Table 1, spots 30, 86, 111) and chaperonin-60 (Table 1, spots 81 and 140) especially in IS plants compared to US plants [71].

## Conclusion

**Figure 6:**
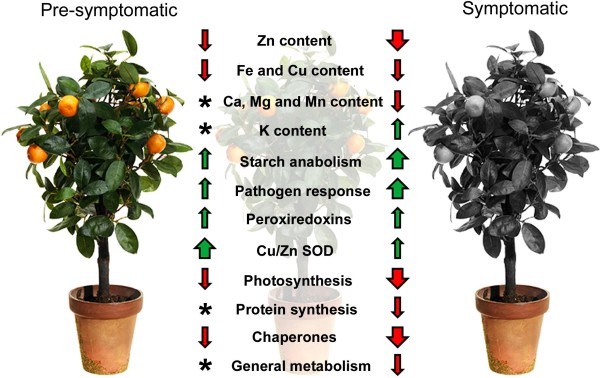
**A schematic summary of observed effects of Las-infection on the nutrient status and protein expression levels in leaves of grapefruit plants that were pre-symptomatic or symptomatic for HLB.** Upward or downward arrows indicate increase or decrease, respectively, while arrow width indicates relative magnitude. Two-year old healthy plants were either graft-inoculated with side shoots from PCR-confirmed Las-infected bud sticks or uninoculated and leaf samples were analyzed at three months post-inoculation (for pre-symptomatic plants) or six months post-inoculation (for symptomatic plants). Asterisks (*) denotes an insignificant effect of Las-infection in pre-symptomatic plants in the accumulation or reduction of Ca, Mg, Mn, and K content as well as the accumulation or reduction of proteins associated with regulation/protein synthesis and general metabolism.

HLB is currently one of the most destructive diseases of citrus and Las has been associated with the disease in many citrus growing regions of the world. Management of HLB remains elusive largely because the physiological and molecular processes involved in HLB-disease development are unresolved. The major findings from our study is summarized in Figure 6 and highlights the potential interrelationships between the protein expression profiles and nutrient status of pre-symptomatic and symptomatic leaves of Las-infected grapefruit plants. We identified 69 proteins that were differentially expressed (13 up-regulated and 56 down-regulated) in response to Las infection. Additionally, we showed a general decrease in nutrient concentrations due to Las-infection particularly those of Fe, Zn, and Cu but an increase in K levels. We propose that the physiological and molecular processes associated with the response of grapefruit plants to Las infection involves: 1) a general decrease in nutrient concentration resulting in the reduced production of proteins associated with photosynthesis, energy production, regulation and protein synthesis/transport; 2) an increase in K concentration to support the activity of an increased production of starch anabolism-related proteins; and 3) an increase in the production of peroxiredoxins, Cu/Zn SOD and pathogen response-related proteins, such as chitinase, miraculin- and lectin-like proteins, which although insufficient to mitigate bacterial spread could, in combination with testing for granule-bound starch synthase and K content, be useful in the development of host-based diagnostic techniques for early detection of HLB-affected citrus plants.

## Methods

### Growth conditions and treatments

Plant growth was performed under controlled conditions in an insect-proof greenhouse at the U.S. Horticulture Research laboratory, U.S. Department of Agriculture, Fort Pierce, Florida. Two-year old grapefruit (*Citrus paradisi* cv. ‘Duncan’) plants from the same progeny were either uninoculated or inoculated by side-grafting with 3–4 cm long bud sticks from PCR-confirmed HLB-affected (showing blotchy mottle and yellow shoots) lemon plants. Each HLB-affected side-graft was protected by covering with plastic tape for 3 weeks [72]. The absence or presence of HLB-associated Las in plants, pre- or post-inoculation, respectively, was confirmed by quantitative real-time PCR using forward primers 5-TCGAGCGTATGCAATACG-3 and reverse primers 5-CGCAATAGGGCATCTTTTTCCATC-3 [73].

Plants were arranged randomly on the greenhouse bench and kept under natural light conditions at a temperature of 23–30°C. Plants were irrigated as needed and fertilized every three weeks using a water-soluble fertilizer mix, 20 N-10P-20 K (Peters Professional, The Scotts Company, Marysville, OH). Micronutrients (Micro Key Palm and Ornamental Formulation, Brandt Consolidated, Springfield, IL) and additional iron (Sequestrene 138 Fe, Becker Underwood, Ames, IA) were applied. Plants were pruned immediately after graft-inoculation to promote new leaf growth and HLB disease development.

Three months post-inoculation, 10–15 fully expanded leaves were collected from three individual plants each from the uninoculated or inoculated group. At this stage the infected plants were pre-symptomatic (no blotchy mottle, yellow shoots or symptoms of nutrient deficiency) but were PCR-positive for Las. Leaf samples from uninoculated or inoculated plants were grouped, respectively, as uninfected control for pre-symptomatic (UP) plants or infected pre-symptomatic (IP) plants. Six months post-inoculation, another set of 10–15 fully expanded leaves was collected from three individual plants each from the uninoculated or inoculated group. At this stage all of the inoculated plants were symptomatic for HLB and PCR-positive for Las. Leaf samples from uninoculated or inoculated plants were grouped, respectively, as uninfected control for symptomatic (US) plants or infected symptomatic (IS) plants. As the plants used for pre-symptomatic stage analysis were different from those used for symptomatic stage analysis, two different groups of control plants, UP and US plants, were used for IP and IS plants, respectively. Harvested leaves were immediately frozen in liquid nitrogen and stored at −80°C until further analysis.

### Protein extraction and quantification

The method used for total leaf protein analysis was modified after Nwugo and Huerta [26]. Leaves from individual plants were pooled and ground to a fine powder in liquid nitrogen using a freezer mill (6850 Freezer/Mill, Wolf Laboratories Ltd., UK). Approximately 0.4 g of leaf powder was transferred to sterile 5 mL polyallomer centrifuge tubes (Beckman Instruments Inc., USA) and suspended in 4.5 mL of chilled solution A [90% (v/v) acetone, 9.9993% (v/v) trichloroacetic acid (TCA), 0.0007% (v/v) Beta-mercaptoethanol]. The mixture was incubated overnight at −80°C followed by centrifugation at 4°C for 20 min at 36,000 *g* (Optima L-70 K Ultracentrifuge, Beckman Coulter Inc., USA). The supernatant was decanted, and the pellet was washed at least three times until the supernatant was clear (not greenish) by resuspension in 4.5 mL of chilled solution B [98.53% (v/v) acetone, 1 mM polymethylsulphonylfluoride (PMSF), 2 mM EDTA, 0.0007% (v/v) Beta-mercaptoethanol], incubation for 1 h at −80°C followed by centrifugation at 4°C for 20 min at 36,000 *g*. The whitish pellet or crude protein extract was then transferred into sterile eppendorf tubes and vacuum-dried (Vacufuge™, Eppendorf, Germany). The dry pellet, which could be stored indefinitely at −80°C, was suspended in 0.5 mL of rehydration/isoelectric focusing (IEF) buffer [8 M Urea, 50 mM DTT, 4% (w/v) CHAPS, 0.2% (v/v) 3/10 ampholytes, 0.002% (w/v) bromophenol blue] and incubated at room temperature (RT) for 30 min to solubilize proteins. Insoluble material was removed by centrifugation at RT at 14,000 *g* for 15 min and 5μL of the supernatant was prepared using the Compat-Able™ Protein Assay Preparation Reagent Set (Pierce, Rockford, IL, USA) for total protein quantification via bicinchoninic acid (BCA) assay (Pierce, Rockford, IL, USA). Total protein extraction and quantification process was repeated three times generating three analytical replicates per plant.

### 2-DE separation and image analysis

For first dimension electrophoresis or IEF, 11-cm long pH 4–7 ReadyStrip IPG strips (Bio-Rad, Hercules, CA, USA) were passively rehydrated overnight at RT with 0.2 mL of IEF buffer containing 1 mg/mL of total solubilized proteins. Rehydrated strips were placed in a PROTEAN IEF cell (Bio-Rad) and IEF was performed at a current limit of 50 μA/per IpG strip at 10°C, in the following steps: active rehydration at 250 V for 9 h; 250 V (linear) for 15 min; 8 kV (linear) for 3 h; and 10 kV (rapid) until a total 60 kVh for a combined total of approximately 70 kVh. Each focused IPG strip was equilibrated by soaking, with mild stirring, in 4 ml of equilibration base buffer 1 (EBB1) [8 M urea, 2% (w/v) sodium dodecyl sulphate (SDS), 50 mM Tris–HCl (pH 8.8), 20% (v/v) glycerol, 1% (w/v) DTT] for 10 min, followed by soaking in 4 ml of EBB2 [same content as EBB1 except DTT was replaced with 2.5% (w/v) iodoacetamide (IAA)]. Second dimension electrophoresis was performed in 8-16% gradient SDS-polyacrylamide Tris–HCl gels (Criterion precast gels, Bio-Rad) in a twelve-gel cell system (Criterion Dodeca Cell, Bio-Rad). Protein spots were visualized by staining with Biosafe Coomassie. Stained gels were scanned (ScanMaker 9800XL, Microtek, USA) under identical conditions and stored in 0.02% NaN_3_ at 4°C.

Gel images were analyzed using the PDQuest software package (version 8.0, Bio-Rad, USA). A total of 36 gels were analyzed representing three analytical replicates per plant and three replicate plants per treatment. The gels were sorted into four into four groups namely: uninfected control for pre-symptomatic (UP) plants, infected pre-symptomatic (IP) plants, uninfected control for symptomatic (US) plants, or infected symptomatic (IS) plants. Gel spots were detected and matched so that a given spot had the same number across all gels. A master gel image containing matched spots across all gels was auto-generated. Extensive analysis using the “Landmark” tool was used to resolve missed matches and spot volumes were normalized according to the total gel image density as suggested by the PDQuest software package. An average spot volume was determined for each spot per group and pair-wise quantitative as well as statistical analysis sets were generated by comparing the average volume of a given spot across all treatments. Only spots that had ≥10-fold increase over background and present in at least six of the nine gels per treatment as well as showed 1.5 fold change (*P* < 0.05) compared to at least one other treatment group were considered to be differentially produced and further analyzed.

### Trypsin digestion and mass spectrometry

Protein spots were manually excised (OneTouch Plus Spotpicker, The Gel company, USA), reduced with DTT, alkylated with IAA, and digested with mass spectrometry grade trypsin in the presence of ProteaseMAX™ Surfactant according to the manufacturer’s protocol (Promega, USA). Acetonitrile extraction was used to enhance peptide recovery. Tryptic-digests were generally analyzed by MALDI-TOF- or LC-MS/MS.

For MALDI-TOF-MS or MS/MS analysis (QSTAR XL Hybrid Quadrupole TOF LC/MS/MS System, Applied Biosystems, USA), the target plate was spotted with 2 μL of a 1:1 (v/v) mixture of tryptic-digest and matrix solution [10 mg/mL α-cyano-4-hydroxycinnamic acid (CHCA) in 50% ACN/ 0.1% TFA]. Mass spectra were acquired in positive TOF MS mode over the mass range of 800 – 4000 Da using 300 one-second cycles with MCA on. A mixture of Des-Arg1-Bradykinin (904.47), Angiotensin I (1296.68), Neurotensin (1672.92) and ACTH (2093.09, 2465.20, 3657.92) monoisotopic [M + H]^+^ mass standards (Anaspec, USA) were used for external calibration. Monoisotopic peaks with S/N >5 were selected as the peptide mass fingerprint (PMF) per spot. Parent ion spectra (MS/MS) was acquired over a mass range of 50 – 4000 Da using 300 one-second cycles with MCA on.

For LC-MS/MS analysis (Ultimate 3000 RLSCnano System linked to Velos LTQ Orbitrap, Thermo Fisher), peptides were solubilized in 0.1% TFA and loaded on to a self-made fused silica trap-column of 100 μm × 2 cm packed with Magic C18 AQ (5 um bead size, 200 Å pore size Michrom Bioresources, Inc.) and washed with 0.2% formic acid at a flow-rate of 10 μL/min for 5 min. The retained peptides were separated on a fused silica column of 75 μm × 50 cm self-packed with Magic C18 AQ (3 um bead size, 200 Å pore size, Michrom Bioresources, Inc.) using a linear gradient from 4 to 45% B (A: 0.1% formic acid, B: 0.08% formic acid, 80% ACN) in 30 min at a flow-rate of 300 nL/min. For each cycle, one full MS was scanned in the Orbitrap with resolution of 60000 from 300–2000 m/z followed by CID fragmentation of 20 most intense peaks. Data dependent acquisition was set for repeat count of 2 and exclusion of 60 sec.

### Protein identification via database queries

Prior to database queries, the Peak Erazor software (v 2.01: Lighthouse data, Odense, Denmark) was used to process peptide mass fingerprints (PMFs) generated from MALDI-TOF-MS analysis as previously described (Nwugo and Huerta, 2011). The MASCOT search engine (Matrix Science, London, UK) was used to find matches of the PMF and MS/MS fragmentation spectra against a custom database containing entries for citrus (*Citrus sinensis* and *Citrus clementina*) available at http://www.citrusgenomedb.org/ and entries for grape (*Vitis vinifera*) available in the NCBI nonreduntant database. The PAC nos. for citrus or Accession nos. for grape entries that matched to our protein/peptide queries at the moment of Mascot search was recorded. Fixed and variable modifications (Cys carbamidomethylation and Met oxidation, respectively) and one missed cleavage were considered. PMF database search was conducted using a maximum mass tolerance of ±100 ppm, while MS/MS ions search were conducted with a mass tolerance of ± 0.6 Da on the parent and 0.3-0.8 Da on fragments; in all cases the peptide charge was +1. Decoy search was done automatically by Mascot on randomized database of equal composition and size. For PMF analysis, the peptide mixtures that produced the highest statistically significant (*P* < 0.05) match scores and accounted for the majority of the peaks present in the mass spectra, were assumed to be positively identified proteins.

LC-MS/MS spectra were also searched via MASCOT against a custom citrus database using the following parameters: precursor mass tolerance 10 ppm, fragment mass tolerance: 0.6 Dalton, fixed modification of carbamidomethylaion on cysteine and variable modification of methionine oxidation. The peptide identification results were filtered using a False-Detection-Rate (FDR) of 1% and only the top match was reported. To gain functional information on identified proteins from MALDI-TOF and LC-MS/MS analysis, homology searches using BLAST_P_ (http: http://www.ncbi.nlm.nih.gov/BLAST) was employed.

### Nutrient status analysis

The macro- and micro-nutrient status of uninfected controls and Las-infected plants was assessed by assaying the concentrations of Ca, K, Mg, Fe, Cu, Mn, and Zn via Inductively-Coupled Plasma Optical Emission Spectroscopy (ICP-OES) in leaf tissues as previously described [74]. Briefly, the same ground leaf tissues used for proteomic analysis was oven-dried and 0.5 g was ashed at 510°C for 9 hrs, allowed to cool, and digested in 10 mL of 1 N HNO_3_ for 1 h. The filtered supernatant was brought to volume (25 mL) and the intensities of atomic emissions at 396.847 nm for Ca, 766.491 nm for K, 279.553 nm for Mg, 238.204 nm for Fe, 327.395 nm for Cu, 257.610 nm for Mn, and 213.857 nm for Zn was measured on an ICP-OES System (Varian Vista Pro CCD Simultaneous ICP-OES attached to Varian SPS 5 Sampler Preparation System, Agilent, USA). Samples were diluted 1:100 in 1 N HNO_3_ prior to Ca, K, and Mg analysis. All containers used for ICP Spectroscopy analysis were acid-washed by soaking overnight in 1 N HNO_3_ before use.

### Statistical analysis

The nutrient concentration data were subjected to analysis of variance (ANOVA) using SigmaPlot software Version 11 (Systat Software, Inc., Point Richmond, California, USA) and means were separated using the Fischer’s Least Significant Difference (FLSD) test at 95% confidence interval (*P* < 0.05). Pair-wise comparisons to determine significant differences in spot volumes between treatments were performed on standardized log_10_ values of protein spot volumes using the Student’s *t*-test analysis at 95% confidence interval (*P* < 0.05) as provided by the PDQuest software.

## Competing interests

The authors declare that they have no competing interests.

## Authors’ contributions

CCN, HL and YPD conceived and designed the experiments and collected samples. CCN conducted experiment and analyzed the data. CCN, HL and ELC wrote the paper. All authors read and approved the final manuscript.

## Supplementary Material

Additional file 1: Table S1 Protein extraction and 2-DE separation parameters of total leaf proteins of Las-infected or uninfected grapefruit plants. Click here for file

Additional file 2: Figure S1 Two-dimensional electrophoresis (2-DE) gel maps of total leaf proteome of grapefruit plants that were infected or uninfected with Las and pre-symptomatic or symptomatic for huanglongbing. (**A**) Representative gel of uninfected control for pre-symptomatic (UP) plants; (**B**) Representative gel of infected pre-symptomatic (IP) plants; (**C**) Representative gel of uninfected control for symptomatic (US) plants; (**D**) Representative gel of infected symptomatic (IS) plants. Two-year old healthy plants were either graft-inoculated with side shoots from PCR-confirmed Las-infected bud sticks or uninoculated and leaf samples were analyzed at three months post-inoculation (for pre-symptomatic plants) or six months post-inoculation (for symptomatic plants). A total of 200 μg of protein was loaded on a pH 4–7 IpG strip and protein spots were visualized by staining with Coomassie Brilliant Blue (CBB). *M*_r_, relative molecular weight; p*I*, isoelectric point. Click here for file

Additional file 3: Figure S2 Panels A-T show magnified views of protein spots that were differentially expressed in leaves of grapefruit plants that were uninfected or infected with Las and pre-symptomatic or symptomatic for HLB. UP, uninfected control for pre-symptomatic plants; IP, infected pre-symptomatic plants; US, uninfected control for symptomatic plants; IS, infected symptomatic plants. Two-year old healthy plants were either graft-inoculated with side shoots from PCR-confirmed Las-infected bud sticks or uninoculated and leaf samples were analyzed at three months post-inoculation (for pre-symptomatic plants) or six months post-inoculation (for symptomatic plants). A total of 200 μg of protein was loaded on a pH 4–7 IpG strip and protein spots were visualized by staining with Coomassie Brilliant Blue (CBB). Click here for file

Additional file 4: Table S2 List of all identified proteins grouped according to multiple spot matches. Click here for file

Additional file 5: Table S3 Protein spots that were differentially produced according to treatment comparisons described in Figure 3. Click here for file

Additional file 6: Appendix S1 Histograms of protein spot volumes highlighting significant differences. Click here for file

Additional file 7: Appendix S2 Mascot match results. Click here for file
