# Supplementary material for: The effect of ‘Candidatus Liberibacter asiaticus’ infection on the proteomic profiles and nutritional status of pre-symptomatic and symptomatic grapefruit (Citrus paradisi) plants
Source: BMC Plant Biol. 2013 Apr 11;13:59. doi: 10.1186/1471-2229-13-59 (PMC3668195; doi:10.1186/1471-2229-13-59)
Supplement: Additional file 1: Table S1 — Protein extraction and 2-DE separation parameters of total leaf proteins of Las-infected or uninfected grapefruit plants. [file 1471-2229-13-59-S1.doc]

**Table S1.** Protein extraction and 2-DE separation parameters of total leaf proteins of Las-infected or uninfected citrus grapefruit plants. Data represents Means ± SD.

| Parameters | Treatments | | | |
| --- | --- | --- | --- | --- |
|  | UP^a^ | IP^b^ | US^c^ | IS^d^ |
| Protein yield^e^ (mg g^-1^) | 10.4 ± 2.5 | 13.3 ± 1.5 | 13.6 ± 1.9 | 12.9 ± 3.1 |
| Number of detected spots | 782 ± 26 | 758 ± 19 | 763 ± 31 | 733 ± 43 |
| Number of matched  spots in replicate gels | 508 ± 12 | 453 ± 31 | 450 ± 52 | 471 ± 85 |
| Number of matched  spots in all gels | 128 | 128 | 128 | 128 |

^a^Uninfected control for pre-symptomatic (UP) plants.

^b^Infected pre-symptomatic (IP) plants.

^c^Uninfected control for symptomatic (US) plants.

^d^Infected symptomatic (IS) plants.

^e^Protein was extracted from 0.4g of fresh leaf tissue per treatment.
