# Supplementary figures and images for: The effect of ‘Candidatus Liberibacter asiaticus’ infection on the proteomic profiles and nutritional status of pre-symptomatic and symptomatic grapefruit (Citrus paradisi) plants
Source: BMC Plant Biol. 2013 Apr 11;13:59. doi: 10.1186/1471-2229-13-59 (PMC3668195; doi:10.1186/1471-2229-13-59)

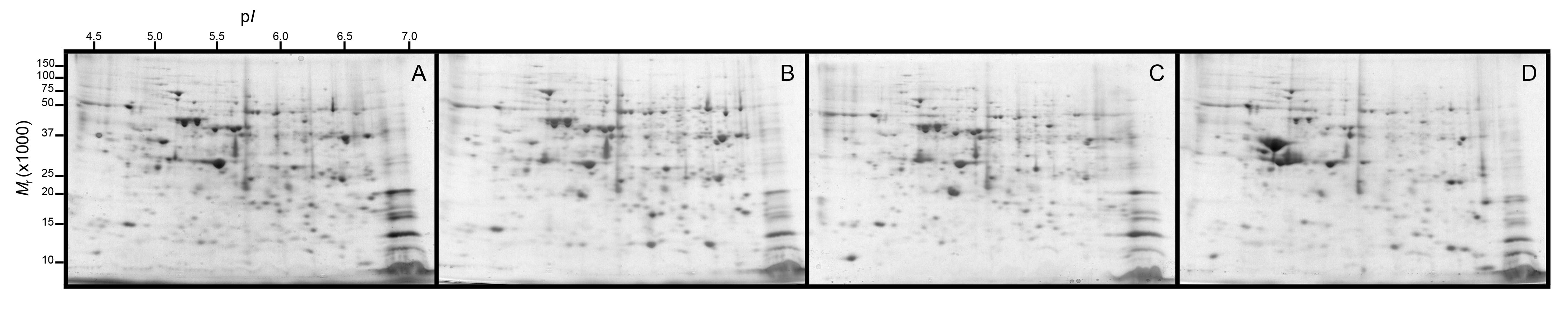

Supplement: Additional file 2: Figure S1 — Two-dimensional electrophoresis (2-DE) gel maps of total leaf proteome of grapefruit plants that were infected or uninfected with Las and pre-symptomatic or symptomatic for huanglongbing. (A) Representative gel of uninfected control for pre-symptomatic (UP) plants; (B) Representative gel of infected pre-symptomatic (IP) plants; (C) Representative gel of uninfected control for symptomatic (US) plants; (D) Representative gel of infected symptomatic (IS) plants. Two-year old healthy plants were either graft-inoculated with side shoots from PCR-confirmed Las-infected bud sticks or uninoculated and leaf samples were analyzed at three months post-inoculation (for pre-symptomatic plants) or six months post-inoculation (for symptomatic plants). A total of 200 μg of protein was loaded on a pH 4–7 IpG strip and protein spots were visualized by staining with Coomassie Brilliant Blue (CBB). Mr, relative molecular weight; pI, isoelectric point. [file 1471-2229-13-59-S2.jpeg]

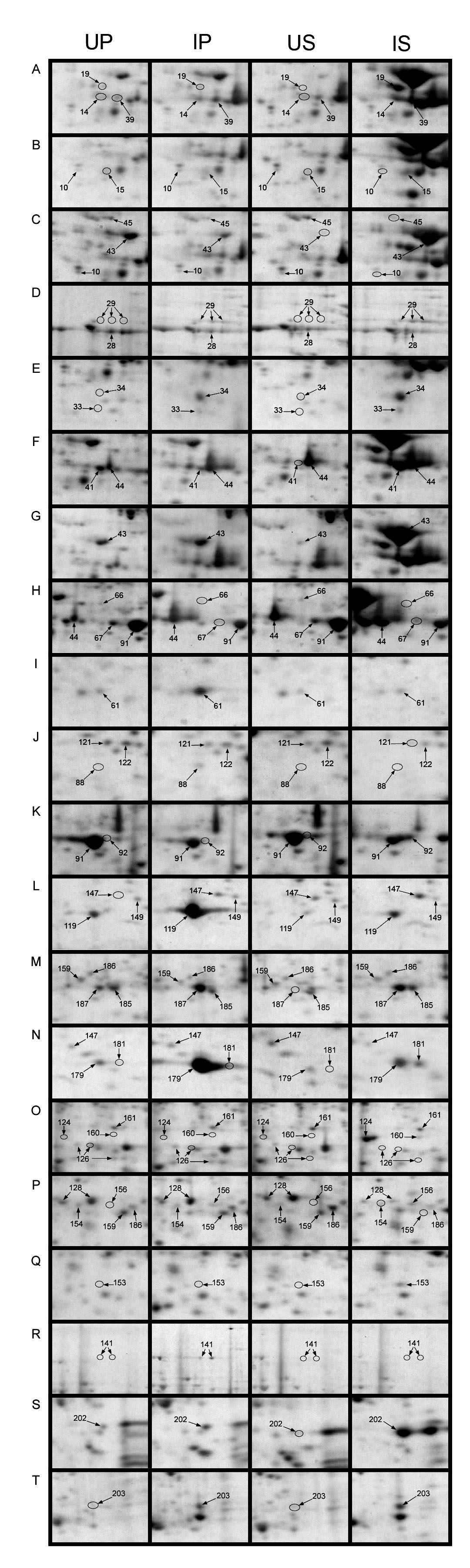

Supplement: Additional file 3: Figure S2 — Panels A-T show magnified views of protein spots that were differentially expressed in leaves of grapefruit plants that were uninfected or infected with Las and pre-symptomatic or symptomatic for HLB. UP, uninfected control for pre-symptomatic plants; IP, infected pre-symptomatic plants; US, uninfected control for symptomatic plants; IS, infected symptomatic plants. Two-year old healthy plants were either graft-inoculated with side shoots from PCR-confirmed Las-infected bud sticks or uninoculated and leaf samples were analyzed at three months post-inoculation (for pre-symptomatic plants) or six months post-inoculation (for symptomatic plants). A total of 200 μg of protein was loaded on a pH 4–7 IpG strip and protein spots were visualized by staining with Coomassie Brilliant Blue (CBB). [file 1471-2229-13-59-S3.jpeg]
