# Supplementary material for: The effect of ‘Candidatus Liberibacter asiaticus’ infection on the proteomic profiles and nutritional status of pre-symptomatic and symptomatic grapefruit (Citrus paradisi) plants
Source: BMC Plant Biol. 2013 Apr 11;13:59. doi: 10.1186/1471-2229-13-59 (PMC3668195; doi:10.1186/1471-2229-13-59)
Supplement: Additional file 4: Table S2 — List of all identified proteins grouped according to multiple spot matches. [file 1471-2229-13-59-S4.doc]

**Table S2.** List of all differentially produced protein spots presented in Figure 1. Spots matching to the same protein are grouped within horizontal lines.

| Spot^a^ | ASV^b^  UP IP US IS | Protein function/name^c^ | NCBI  Accession # | Theoretical^d^ | | S^e^ | M^f^ | E^g^ |
| --- | --- | --- | --- | --- | --- | --- | --- | --- |
|  |  |  |  | *M*_r_ | p*I* |  |  |  |
|  |  | ***CO_2_ assimilation/Photosynthesis*** |  |  |  |  |  |  |
| 3 |  | Ribulose 1,5-bisphosphate carboxylase/  oxygenase large subunit(chloroplast) | gi|114329664 | 53950 | 6.19 | 143 | 19 | 41 |
| 28 |  | Ribulose 1,5-bisphosphate carboxylase/  oxygenase large subunit(chloroplast) | gi|114329664 | 53950 | 6.19 | 94 | 14 | 36 |
| 208 |  | Ribulose 1,5-bisphosphate carboxylase/  oxygenase large subunit (chloroplast) | gi|114329664 | 53950 | 6.19 | 106 | 17 | 41 |
| 211 |  | Ribulose-1,5-bisphosphate carboxylase/oxygenase small subunit | gi|24940138 | 20521 | 9.16 | 92 | 8 | 41 |
| 49 |  | Chloroplast ribulose-1,5-bisphosphate carboxylase/oxygenase activase large protein isoform | gi|115334977 | 41738 | 5.07 | 150 | 24 | 56 |
| 71 |  | Ribulose bisphosphate carboxylase/oxygenase activase 1, chloroplast precursor, putative | gi|255584538 | 51073 | 5.33 | 145 | 20 | 50 |
| 72 |  | Ribulose bisphosphate carboxylase/oxygenase activase 1, chloroplast precursor, putative | gi|255584538 | 47200 | 5.94 | 144 | 21 | 55 |
| 101 |  | Chloroplast ribulose-1,5-bisphosphate carboxylase/oxygenase activase small protein isoform | gi|115334975 | 47200 | 5.94 | 148 | 26 | 63 |
| 67 |  | Oxygen evolving enhancer protein 1 | gi|326467059 | 29262 | 5.32 | 133 | 14 | 67 |
| 91 |  | Oxygen evolving enhancer protein 1 | gi|326467059 | 29262 | 5.32 | 134 | 15 | 70 |
| 113 |  | Rubisco subunit binding-protein beta subunit, putative | gi|255564820 | 65086 | 5.85 | 211 | 25 | 50 |
| 126 |  | Carbonic anhydrase, putative | gi|255568812 | 28499 | 5.51 | 135 | 13 | 56 |
| 159 |  | Carbonic anhydrase 1 | gi|30678350 | 28483 | 6.39 | 78 | 8 | 38 |
| 164 |  | Photosystem II stability/assembly  factor HCF136, chloroplast precursor, putative | gi|255559812 | 45094 | 8.46 | 147 | 10 | 38 |
| 180^h^ |  | PSI 9 kDa protein | gi|224365649 | 9545 | 6.67 | 51 | 3 | 43 |
|  |  |  |  |  |  |  |  |  |
|  |  |  |  |  |  |  |  |  |
| 214^h^ |  | Oxygen-evolving enhancer protein 2, chloroplastic | gi|225446775 | 26777 | 8.63 | 48 | 6 | 32 |
|  |  | ***Redox homeostasis*** |  |  |  |  |  |  |
| 34^i^ |  | Peroxiredoxins, prx-1, prx-2, prx-3, putative | gi|255578581 | 29299 | 8.38 |  |  |  |
| 119^i^ |  | Cu/Zn superoxide dismutase | gi|2274917 | 12784 | 5.82 |  |  |  |
| 121 |  | Putative thioredoxin-dependent peroxidase | gi|119367465 | 17443 | 5.15 | 119 | 7 | 70 |
| 128 |  | Ascorbate peroxidase 2 | gi|221327589 | 27724 | 5.55 | 162 | 15 | 74 |
| 161 |  | L-ascorbate peroxidase T,  chloroplastic-like isoform 2 | gi|359492510 | 42300 | 8.61 | 223 | 18 | 50 |
| 147^i^ |  | 2Fe-2S ferredoxin-like protein | gi|18397961 | 17602 | 7.75 |  |  |  |
| 188 |  | Coproporphyrinogen III oxidase,  putative | gi|255554717 | 39324 | 7.66 | 162 | 17 | 47 |
| 189^h^ |  | Isoflavone reductase related protein | gi|3243234 | 34281 | 5.92 | 54 | 4 | 19 |
| 205 |  | Cinnamoyl-CoA reductase, putative | gi|255556687 | 35554 | 6.16 | 67 | 9 | 39 |
| 209 |  | Catalase | gi|19070130 | 57669 | 6.64 | 110 | 13 | 35 |
|  |  | ***Pathogen response*** |  |  |  |  |  |  |
| 14^i^ |  | Chloroplastic light/drought-induced stress protein | gi|22261807 | 35216 | 5.24 |  |  |  |
| 15^i^ |  | Acidic class I chitinase | gi|23496445 | 34123 | 4.70 |  |  |  |
| 19 |  | Acidic class I chitinase | gi|23496445 | 36735 | 4.81 | 64 | 8 | 24 |
| 39 |  | Lectin-related protein precursor | gi|11596188 | 29272 | 5.10 | 72 | 7 | 32 |
| 44 |  | Lectin-related protein precursor | gi|11596188 | 29272 | 5.10 | 85 | 7 | 32 |
| 41^h^ |  | Chitinase | gi|1220144 | 32459 | 5.06 | 53 | 5 | 20 |
| 43 |  | Chitinase | gi|1220144 | 36735 | 4.81 | 73 | 11 | 41 |
| 66^i^ |  | Chitinase | gi|1220144 | 31909 | 5.06 |  |  |  |
| 88^i^ |  | Chitinase | gi|1220144 | 31909 | 5.06 |  |  |  |
| 92^i^ |  | Chitinase | gi|1220144 | 31909 | 5.06 |  |  |  |
| 124 |  | Chitinase | gi|1220144 | 36735 | 4.81 | 110 | 11 | 45 |
| 160^i^ |  | Chitinase | gi|1220144 | 31909 | 5.06 |  |  |  |
| 141^i^ |  | CAP160 protein | gi|22327778 | 65970 | 5.07 |  |  |  |
| 179^i^ |  | PR-4 type protein | gi|3511147 | 15227 | 5.50 |  |  |  |
| 153 |  | Putative miraculin-like protein 2 | gi|119367468 | 17806 | 6.74 | 105 | 7 | 49 |
| 156^i^ |  | Miraculin-like protein 2 | gi|11596180 | 25652 | 6.10 |  |  |  |
| 181^i^ |  | Miraculin-like protein 2 | gi|87299377 | 24120 | 5.61 |  |  |  |
| 187^i^ |  | Miraculin-like protein 2 | gi|87299377 | 24120 | 5.61 |  |  |  |
| 202 |  | Putative miraculin-like protein 2 | gi|119367468 | 23610 | 8.18 | 126 | 9 | 54 |
| 203^i^ |  | Miraculin-like protein 2 | gi|87299377 | 24120 | 5.61 |  |  |  |
|  |  | ***Regulation/Protein synthesis*** |  |  |  |  |  |  |
| 16 |  | 31 kDa ribonucleoprotein, chloroplastic | gi|225456840 | 38020 | 4.55 | 64 | 8 | 29 |
| 58 |  | Elongation factor Ts | gi|357500731 | 82705 | 4.68 | 77 | 11 | 23 |
| 97 |  | Proteasome subunit alpha type, putative | gi|255538698 | 29940 | 5.15 | 109 | 10 | 49 |
| 100 |  | Glutamine synthetase plant, putative | gi|255551511 | 48172 | 6.29 | 183 | 21 | 68 |
| 105 |  | Glutamine synthetase plant, putative | gi|255551511 | 48172 | 6.29 | 218 | 17 | 59 |
| 116 |  | ATP-dependent zinc metalloprotease FTSH 2, chloroplastic-like | gi|225446693 | 75921 | 6.44 | 197 | 28 | 51 |
| 136 |  | 26S protease regulatory subunit 6b, putative | gi|255565346 | 44701 | 5.49 | 69 | 9 | 30 |
| 138 |  | Mitochondrial processing peptidase  alpha  subunit, putative | gi|255546263 | 50379 | 5.91 | 163 | 20 | 58 |
| 149 |  | Nucleoside diphosphate kinase 1 | gi|19570344 | 16349 | 5.93 | 80 | 7 | 56 |
| 152^h^ |  | Transcription factor homolog  (Btf3-like) protein | gi|33945882 | 17821 | 5.93 | 61 | 3 | 35 |
| 165 |  | Serine-type peptidase | gi|270342123 | 46267 | 8.24 | 128 | 8 | 30 |
| 170 |  | S-adenosylmethionine synthetase, putative | gi|255548295 | 43620 | 5.65 | 217 | 26 | 71 |
| 178 |  | Nucleoside diphosphate kinase,  putative | gi|255540363 | 14819 | 6.92 | 68 | 4 | 35 |
| 185 |  | DHAR class glutathione transferase DHAR2 | gi|283135906 | 23962 | 6.18 | 110 | 8 | 45 |
| 198 |  | Alanine aminotransferase 2 isoform 2 | gi|359495900 | 54000 | 6.00 | 92 | 13 | 34 |
| 206 |  | mRNA binding protein precursor | gi|350534514 | 43638 | 7.70 | 97 | 14 | 51 |
| 207 |  | Alanine aminotransferase 2 isoform 2 | gi|359495900 | 54000 | 6.00 | 188 | 24 | 57 |
|  |  | ***Chaperones*** |  |  |  |  |  |  |
| 10^h^ |  | Putative FKBP-type peptidyl-prolyl  cis-trans isomerase | gi|51471872 | 22889 | 4.78 | 46 | 6 | 35 |
| 45 |  | Peptidyl-prolyl cis-trans isomerase, putative | gi|255552604 | 48362 | 5.04 | 99 | 14 | 36 |
| 57 |  | Chloroplast HSP70 | gi|124245039 | 76759 | 5.31 | 168 | 22 | 35 |
| 81 |  | Chaperonin-60alpha | gi|15226314 | 55491 | 4.86 | 210 | 22 | 60 |
| 140 |  | Chaperonin-60kD, ch60, putative | gi|255554262 | 56809 | 5.19 | 79 | 12 | 32 |
| 30 |  | Heat shock protein, putative | gi|255555659 | 73678 | 5.10 | 96 | 13 | 22 |
| 86 |  | Heat shock protein 70 | gi|211906496 | 71346 | 5.10 | 118 | 22 | 47 |
| 111 |  | 70 kDa heat shock cognate protein 1 | gi|45331281 | 71381 | 5.11 | 75 | 9 | 21 |
|  |  | ***Energy/Metabolisms*** |  |  |  |  |  |  |
| 20 |  | Protein grpE-like | gi|225439145 | 24749 | 4.61 | 67 | 8 | 47 |
| 29^i^ |  | Granule-bound starch synthase | gi|223029784 | 67320 | 8.56 |  |  |  |
| 33^i^ |  | Granule-bound starch synthase | gi|223029784 | 67320 | 8.56 |  |  |  |
| 61^i^ |  | Granule-bound starch synthase | gi|223029784 | 67320 | 8.56 |  |  |  |
| 70 |  | Sedoheptulose-1,7-bisphosphatase, chloroplast, putative | gi|255579134 | 42768 | 5.82 | 67 | 15 | 31 |
| 75 |  | Beta-tubulin | gi|223018283 | 50941 | 4.76 | 197 | 27 | 60 |
| 78 |  | Alpha-tubulin | gi|134035496 | 42534 | 5.82 | 102 | 15 | 59 |
| 95 |  | Caffeoyl CoA O-methyltransferase 1 | gi|229368458 | 20972 | 5.39 | 74 | 8 | 57 |
| 103 |  | Phosphoribulose kinase, putative | gi|255555933 | 45558 | 5.97 | 141 | 17 | 61 |
| 106 |  | ATP synthase beta subunit, putative | gi|255582911 | 59862 | 6.06 | 152 | 22 | 51 |
| 134 |  | ATP synthase beta subunit, putative | gi|255582911 | 59862 | 6.06 | 278 | 27 | 63 |
| 112 |  | Transitional endoplasmic reticulum ATPase, putative | gi|255556938 | 90244 | 5.14 | 265 | 27 | 47 |
| 122^h^ |  | Bis(5'-adenosyl)-triphosphatase-like | gi|356539734 | 16962 | 6.07 | 48 | 4 | 40 |
| 130 |  | Pyruvate dehydrogenase, putative | gi|255543140 | 39870 | 5.95 | 67 | 11 | 29 |
| 132 |  | Alcohol dehydrogenase, putative | gi|255568816 | 41894 | 8.77 | 141 | 11 | 42 |
| 139 |  | 2-phospho-D-glycerate hydrolase | gi|289600010 | 48059 | 5.54 | 142 | 15 | 53 |
| 154 |  | Carboxymethylenebutenolidase,  putative | gi|255567721 | 30236 | 7.10 | 74 | 7 | 37 |
| 167 |  | Phosphoglycerate kinase, putative | gi|255544584 | 38338 | 9.37 | 108 | 10 | 38 |
| 171 |  | Sorbitol dehydrogenase-like protein | gi|21553353 | 39923 | 6.33 | 119 | 11 | 36 |
| 172 |  | 2-phospho-D-glycerate hydrolase | gi|289600010 | 48059 | 5.54 | 235 | 20 | 64 |
| 173 |  | Putative 2-3 biphosphoglycerate  mutase | gi|239056191 | 61119 | 5.65 | 170 | 16 | 42 |
| 186 |  | Triosphosphate isomerase-like protein type II | gi|262410515 | 27160 | 5.74 | 150 | 11 | 65 |
| 191 |  | Malate dehydrogenase | gi|211906490 | 35859 | 6.10 | 196 | 19 | 66 |
| 199 |  | Aldehyde dehydrogenase, putative | gi|255540719 | 52969 | 5.92 | 198 | 19 | 52 |
|  |  | ***Unknown*** |  |  |  |  |  |  |
| 144 |  | Uncharacterized protein | gi|225443738 | 66158 | 8.50 | 78 | 7 | 20 |
| 197 |  | Hypothetical protein VITISV_021486 | gi|147834040 | 44041 | 5.98 | 68 | 7 | 24 |

^a^ The spot numbers correspond to the numbers given in Figure 2 and Figure S2.

^b^ Average spot volume per treatment group; UP, uninfected reference for pre-symptomatic plants; IP, infected pre-symptomatic plants; US, uninfected reference for symptomatic plants; IS, infected symptomatic plants. Average spot volumes separated by letters to show significant difference are presented in Appendix S1.

^c^ Protein function/name was determined by http:/www.ncbi.nlm.nih.gov/BLAST/.

^d^ Theoretical nominal mass (*M*_r_) and isoelectric point (p*I*) were calculated by http:/www.expasy.org/. Observed *M*_r_ and p*I* can be extrapolated from Figure 1.

^e^ Mascot score of protein hit.

^f^ Number of matched peptide masses. The sequences of PMF-matched peptides per spot are provided in Appendix S2.

^g^ Percent sequence coverage of matched peptides.

^h^ Protein identification confirmed by MALDI-TOF-MS/MS. Sequencing information is provided in Appendix S2.

^i^ Protein identification confirmed by LC-MS/MS. Sequencing information is provided in Appendix S3.
