# Supplementary material for: The effect of ‘Candidatus Liberibacter asiaticus’ infection on the proteomic profiles and nutritional status of pre-symptomatic and symptomatic grapefruit (Citrus paradisi) plants
Source: BMC Plant Biol. 2013 Apr 11;13:59. doi: 10.1186/1471-2229-13-59 (PMC3668195; doi:10.1186/1471-2229-13-59)
Supplement: Additional file 5: Table S3 — Protein spots that were differentially produced according to treatment comparisons described in Figure 3. [file 1471-2229-13-59-S5.doc]

**Table S3.** Assigned spot numbers of identified protein spots whose volumes changed according to treatment comparisons described in Fig. 3B. The spots matched to proteins described in Tables 1 and 2.

| Treatment comparisons | Up-accumulated | Down-accumulated |
| --- | --- | --- |
| UP *vs*. IP* | 14, 15, 29, 33, 34, 44, 61, 66, 88, 119, 141, 147, 156, 179, 187, 203 | 67, 78, 86, 91, 152, 154, 167, 170, 180, 209, 214 |
| IP *vs*. IS* | 14, 15, 19, 29, 33, 39, 41, 43, 44, 66, 92, 124, 147, 153, 160, 181, 202 | 3, 10, 16, 20, 28, 30, 49, 57, 58, 61, 67, 70, 71, 72, 75, 81, 86, 88, 91, 95, 97, 100, 101, 103, 105, 106, 111, 112, 113, 116, 119, 121, 122, 126, 128, 130, 132, 134, 136, 138, 139, 140, 141, 144, 149, 152, 154, 159, 161, 164, 165, 167, 171, 172, 173, 178, 185, 186, 188, 189, 191, 197, 198, 199, 205, 206, 207, 208, 209, 211 |
| US *vs*. IS* | 14, 15, 19, 29, 33, 34, 39, 41, 43, 66, 92, 119, 124, 153, 156, 160, 179, 181, 187, 202, 203 | 3, 10, 16, 20, 28, 30, 45, 49, 57, 58, 67, 70, 71, 72, 75, 78, 81, 86, 91, 95, 97, 100, 101, 103, 105, 106, 111, 112, 113, 116, 121, 122, 126, 128, 130, 132, 134, 136, 138, 139, 140, 144, 149, 152, 154, 159, 161, 164, 165, 167, 170, 171, 172, 173, 178, 180, 185, 186, 188, 189, 191, 197, 198, 199, 205, 206, 207, 208, 209, 211, 214 |
| UP *vs*. US* | 16, 30, 44, 45, 58, 66, 71, 72, 95, 147, 178, 205 | 20, 41, 43, 61, 67, 86, 106, 119, 136, 153, 154, 167, 179, 185, 187, 202, 203, 209 |

UP, uninfected control for pre-symptomatic plants; IP, infected pre-symptomatic plants; US, uninfected control for symptomatic plants; IS, infected symptomatic plants.*, reference for comparison.
