# Supplementary material for: The effect of ‘Candidatus Liberibacter asiaticus’ infection on the proteomic profiles and nutritional status of pre-symptomatic and symptomatic grapefruit (Citrus paradisi) plants
Source: BMC Plant Biol. 2013 Apr 11;13:59. doi: 10.1186/1471-2229-13-59 (PMC3668195; doi:10.1186/1471-2229-13-59)
Supplement: Additional file 6: Appendix S1 — Histograms of protein spot volumes highlighting significant differences. [file 1471-2229-13-59-S6.pdf]

Supplemental document 1. Histograms showing average spot volumes of 97 identified protein spots that were differentially produced in leaves of Las-infected citrus grapefruit plants that are pre-symptomatic or symptomatic for HLB. UP, uninfected reference for pre-symptomatic plants; IP, infected pre-symptomatic plants; US, uninfected reference for symptomatic plants; IS, infected symptomatic plants. Bars with the same letter are not significantly different at $P < 0.05$.

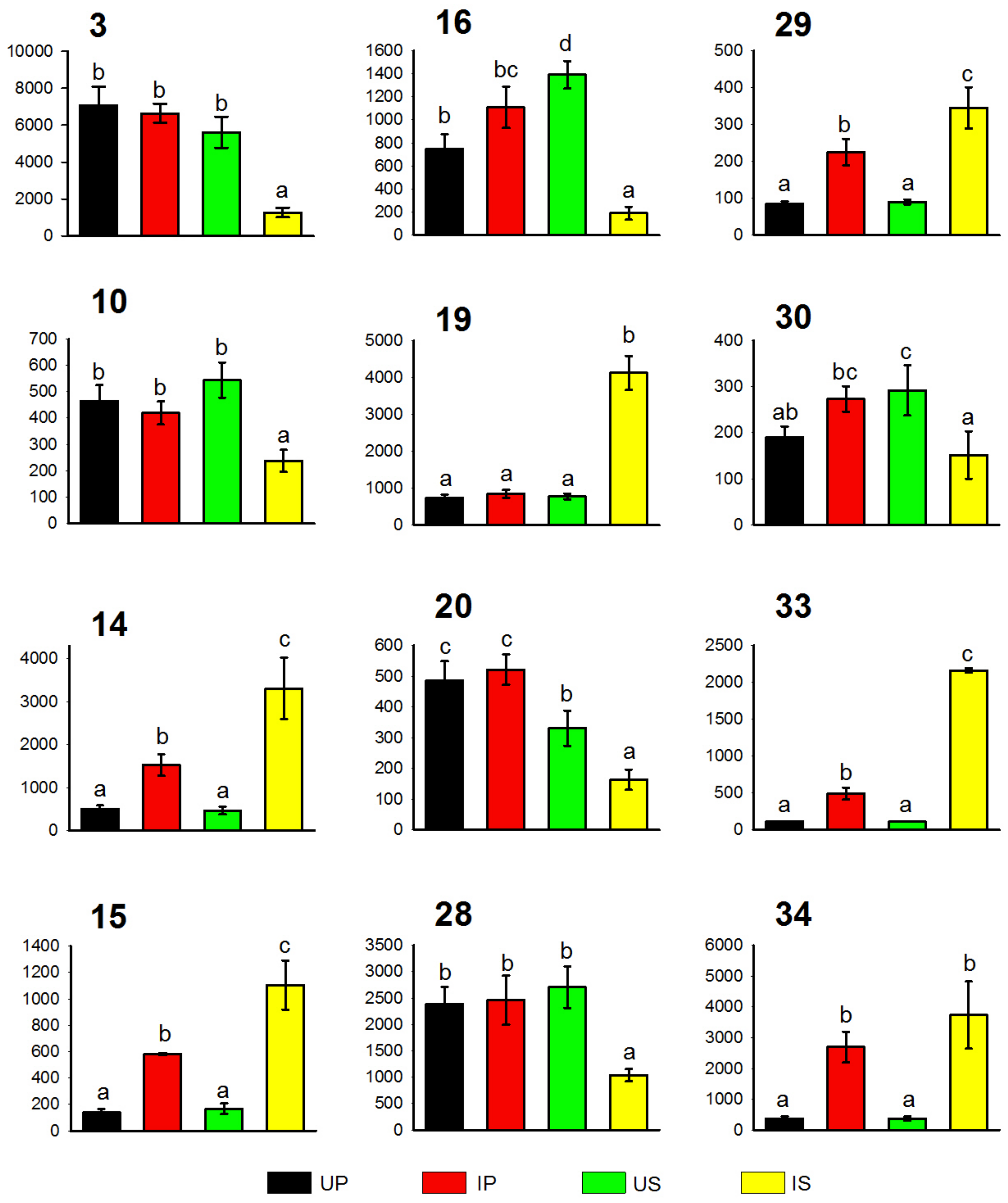
3
10000
8000
6000
4000
2000
0
b
b
b
a
16
1600
1400
1200
1000
800
600
400
200
0
b
bc
d
a
29
500
400
300
200
100
0
a
b
a
c
10
700
600
500
400
300
200
100
0
b
b
b
a
19
5000
4000
3000
2000
1000
0
a
a
a
b
30
400
300
200
100
0
ab
bc
c
a
14
4000
3000
2000
1000
0
a
b
a
c
20
600
500
400
300
200
100
0
c
c
b
a
33
2500
2000
1500
1000
500
0
a
b
a
c
15
1400
1200
1000
800
600
400
200
0
a
b
a
c
28
3500
3000
2500
2000
1500
1000
500
0
b
b
b
a
34
6000
5000
4000
3000
2000
1000
0
a
b
a
b
UP
IP
US
IS

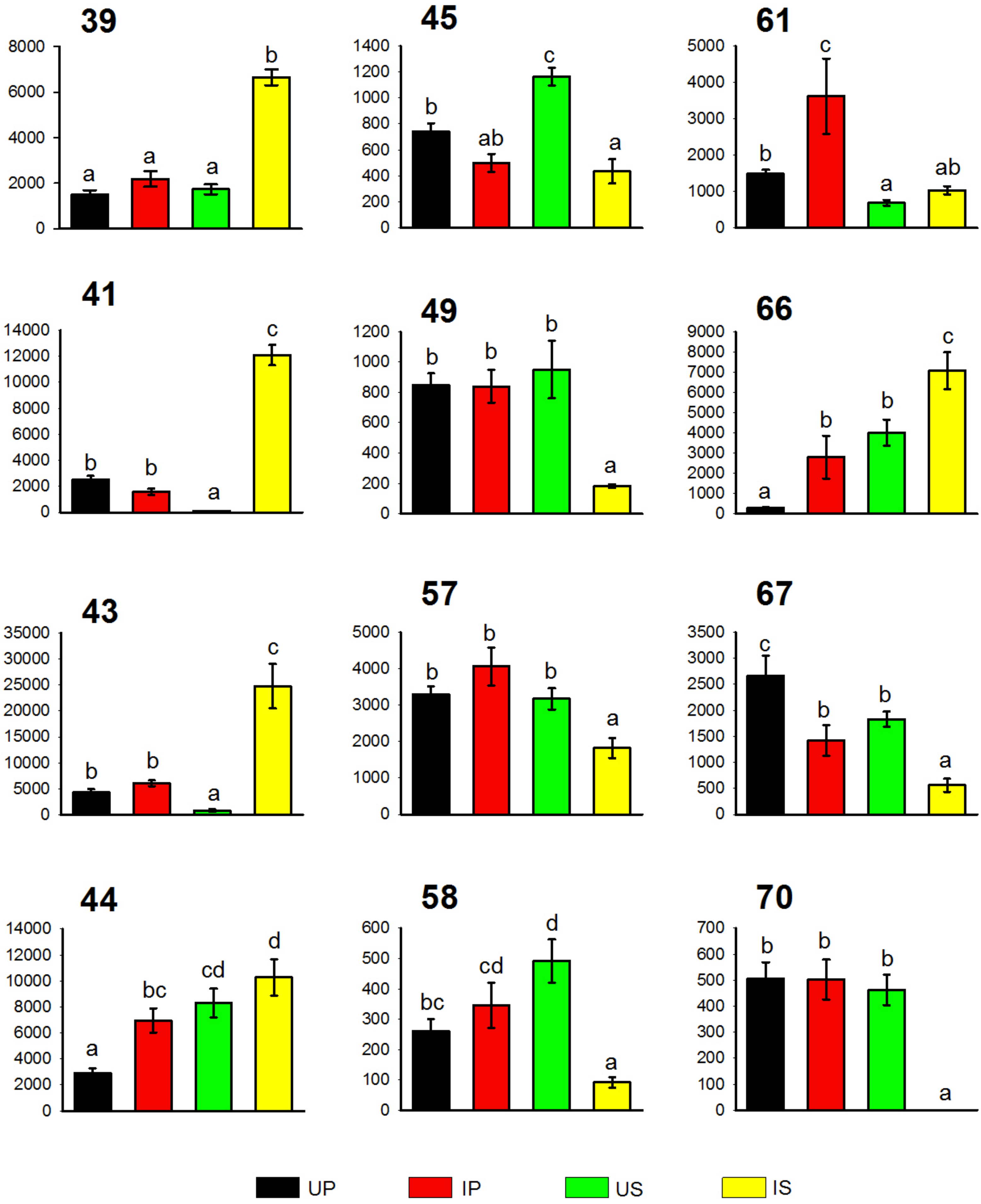
39
8000
6000
4000
2000
0
a
a
a
b
45
1400
1200
1000
800
600
400
200
0
b
ab
c
a
61
5000
4000
3000
2000
1000
0
b
c
a
ab
41
14000
12000
10000
8000
6000
4000
2000
0
b
b
a
c
49
1200
1000
800
600
400
200
0
b
b
b
a
66
9000
8000
7000
6000
5000
4000
3000
2000
1000
0
a
b
b
c
43
35000
30000
25000
20000
15000
10000
5000
0
b
b
a
c
57
5000
4000
3000
2000
1000
0
b
b
b
a
67
3500
3000
2500
2000
1500
1000
500
0
c
b
b
a
44
14000
12000
10000
8000
6000
4000
2000
0
a
bc
cd
d
58
600
500
400
300
200
100
0
bc
cd
d
a
70
700
600
500
400
300
200
100
0
b
b
b
a
UP
IP
US
IS

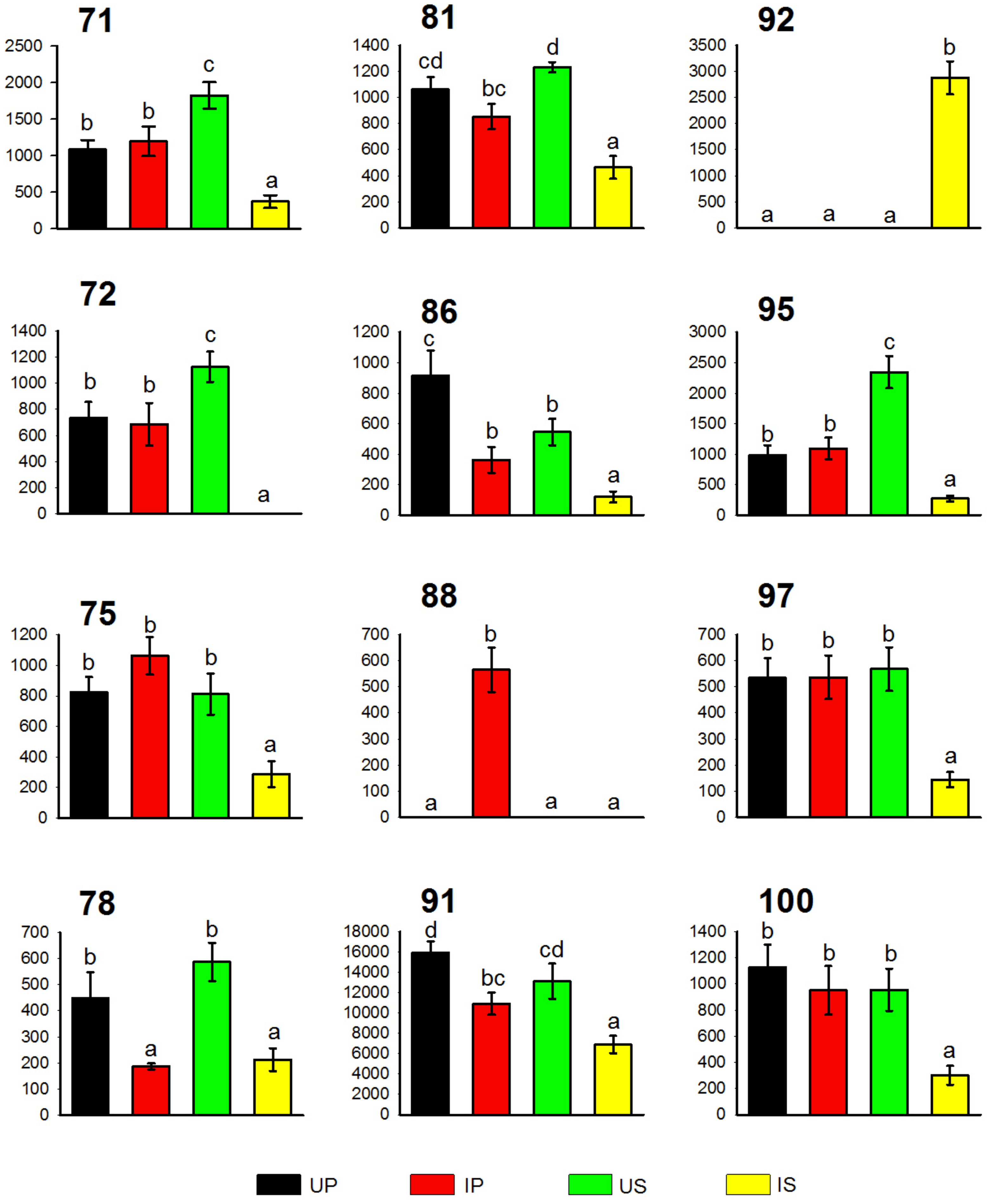
71
2500
2000
1500
1000
500
0
b
b
c
a
81
1400
1200
1000
800
600
400
200
0
cd
bc
d
a
92
3500
3000
2500
2000
1500
1000
500
0
a
a
a
b
72
1400
1200
1000
800
600
400
200
0
b
b
c
a
86
1200
1000
800
600
400
200
0
c
b
b
a
95
3000
2500
2000
1500
1000
500
0
b
b
c
a
75
1200
1000
800
600
400
200
0
b
b
b
a
88
700
600
500
400
300
200
100
0
a
b
a
a
97
700
600
500
400
300
200
100
0
b
b
b
a
78
700
600
500
400
300
200
100
0
b
a
b
a
91
18000
16000
14000
12000
10000
8000
6000
4000
2000
0
d
bc
cd
a
100
1400
1200
1000
800
600
400
200
0
b
b
b
a
UP
IP
US
IS

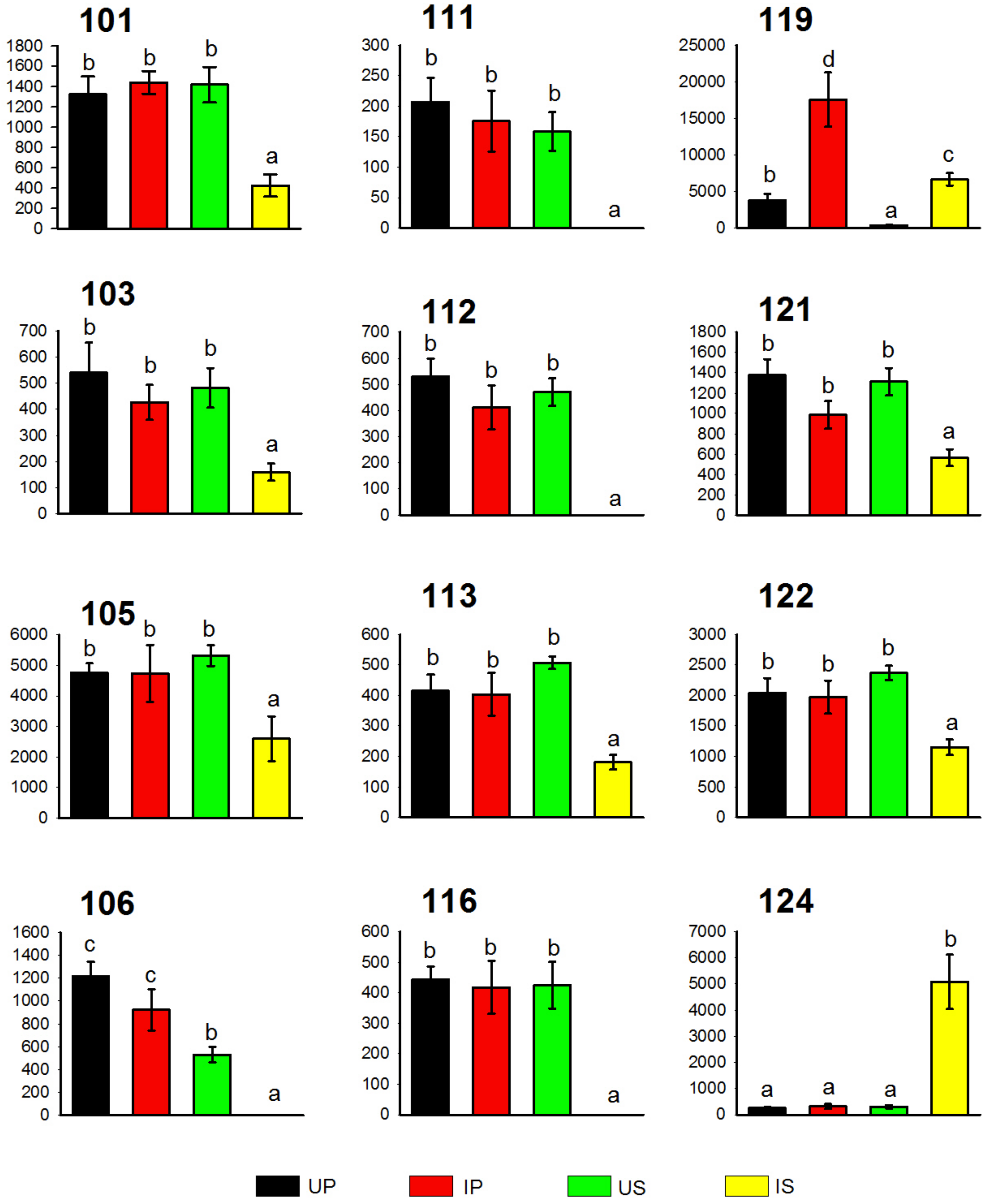

101
111
119
103
112
121
105
113
122
106
116
124
UP
IP
US
IS

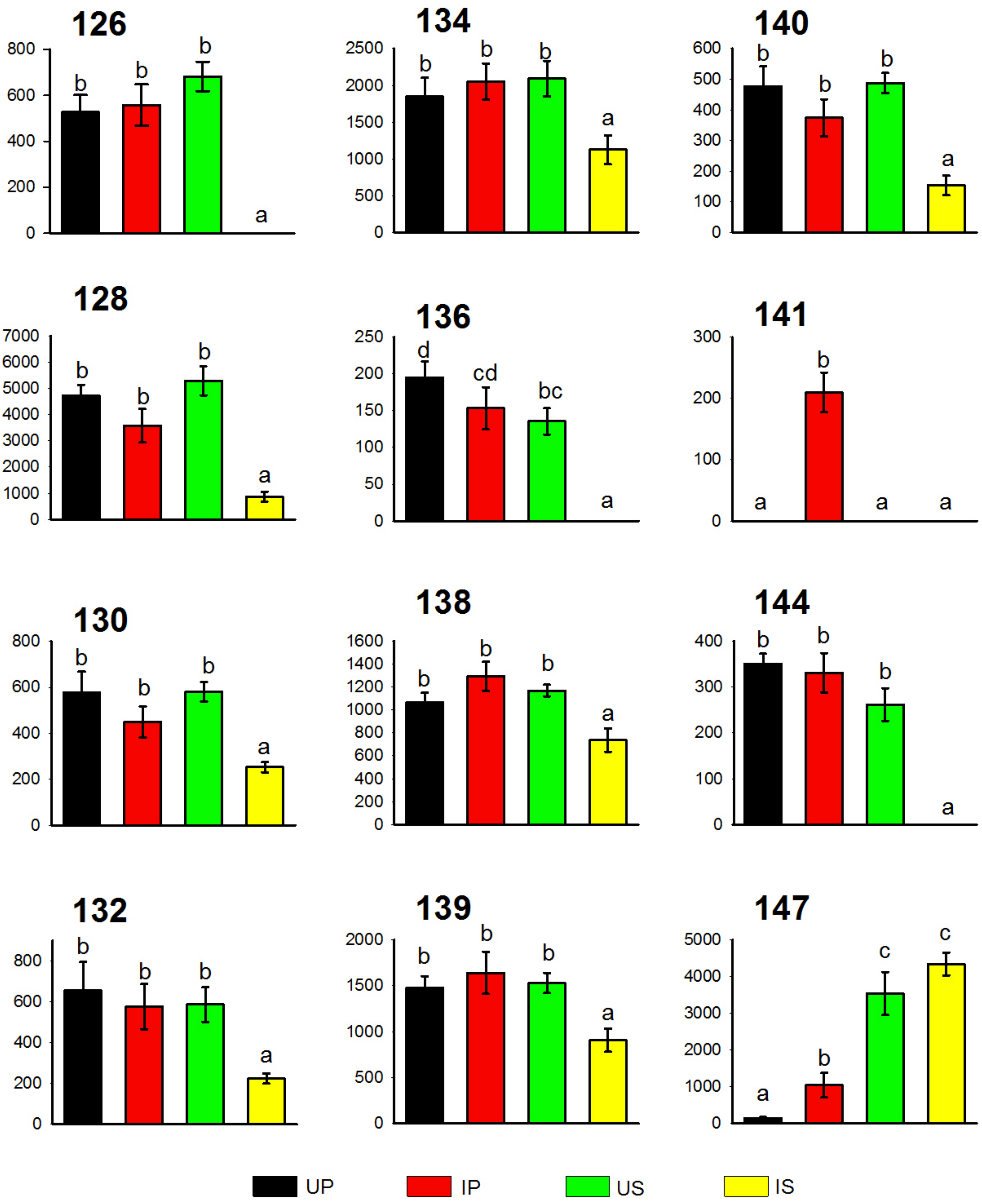

126
800
600
400
200
0
b
b
b
a
134
2500
2000
1500
1000
500
0
b
b
b
a
140
600
500
400
300
200
100
0
b
b
b
a
128
7000
6000
5000
4000
3000
2000
1000
0
b
b
b
a
136
250
200
150
100
50
0
d
cd
bc
a
141
300
200
100
0
a
b
a
a
130
800
600
400
200
0
b
b
b
a
138
1600
1400
1200
1000
800
600
400
200
0
b
b
b
a
144
400
300
200
100
0
b
b
b
a
132
800
600
400
200
0
b
b
b
a
139
2000
1500
1000
500
0
b
b
b
a
147
5000
4000
3000
2000
1000
0
a
b
c
c
UP
IP
US
IS

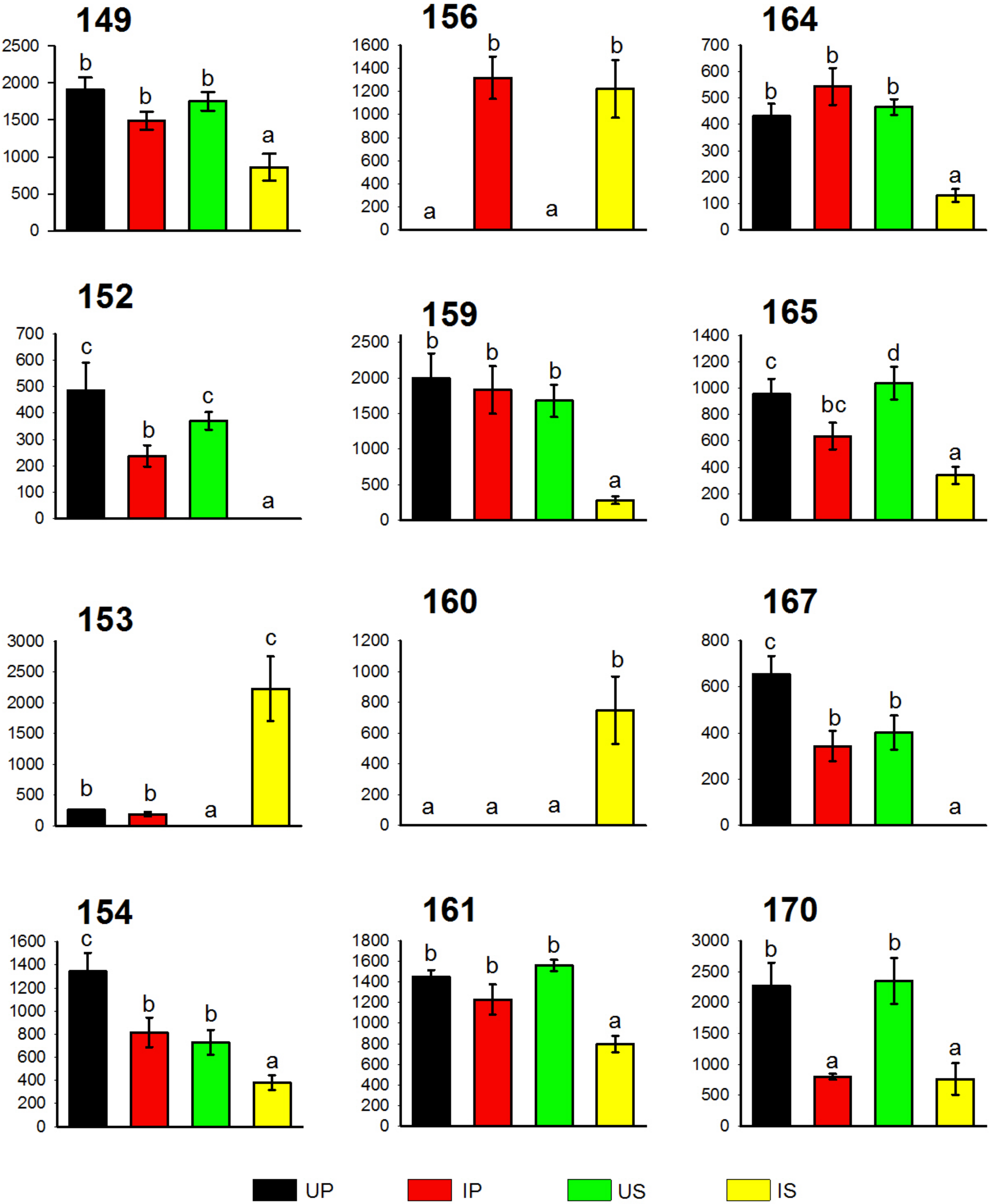
149
2500
2000
1500
1000
500
0
b
b
b
a
156
1600
1400
1200
1000
800
600
400
200
0
a
b
a
b
164
700
600
500
400
300
200
100
0
b
b
b
a
152
700
600
500
400
300
200
100
0
c
b
c
a
159
2500
2000
1500
1000
500
0
b
b
b
a
165
1400
1200
1000
800
600
400
200
0
c
bc
d
a
153
3000
2500
2000
1500
1000
500
0
b
b
a
c
160
1200
1000
800
600
400
200
0
a
a
a
b
167
800
600
400
200
0
c
b
b
a
154
1600
1400
1200
1000
800
600
400
200
0
c
b
b
a
161
1800
1600
1400
1200
1000
800
600
400
200
0
b
b
b
a
170
3000
2500
2000
1500
1000
500
0
b
a
b
a
UP
IP
US
IS

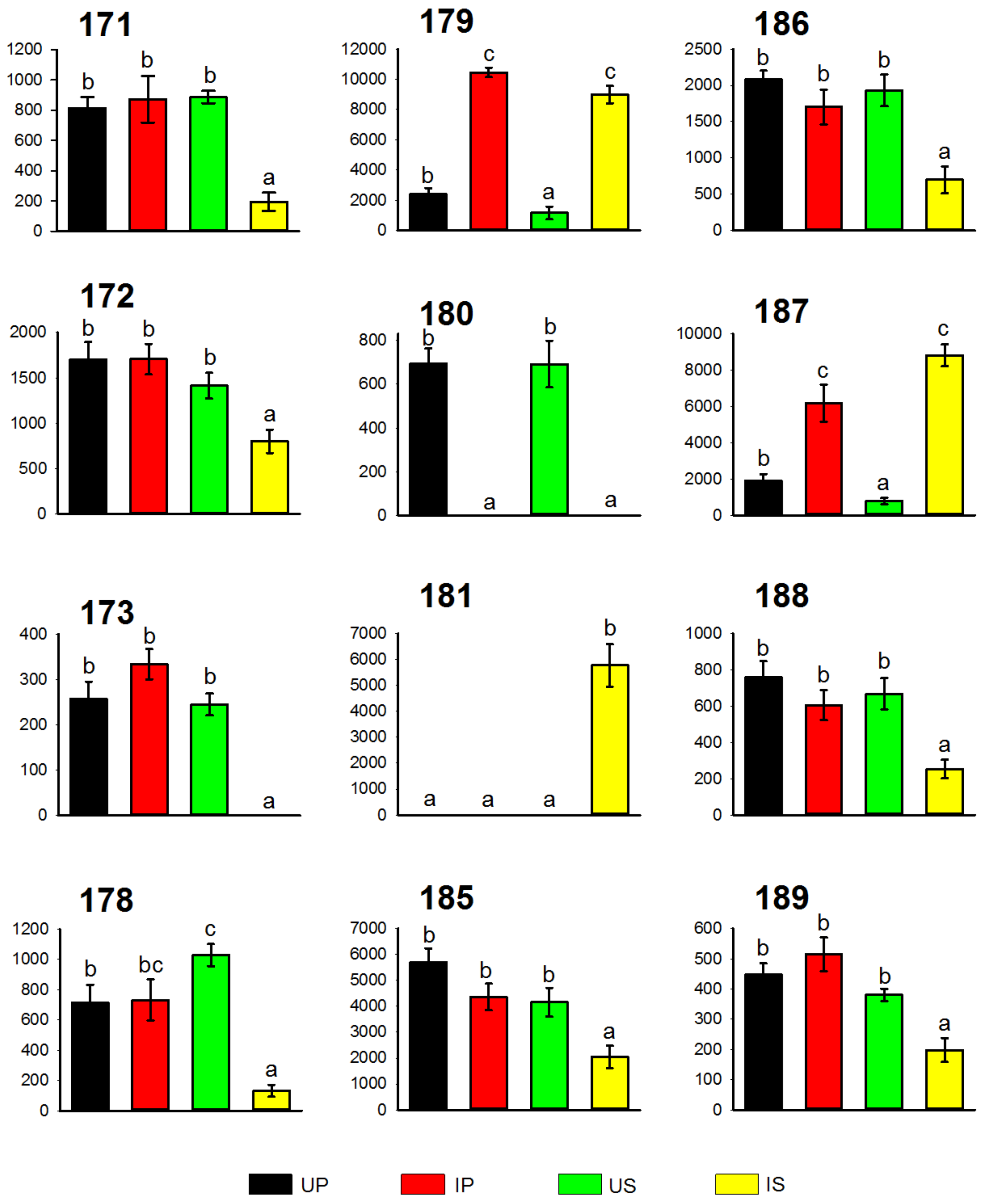

171
1200
1000
800
600
400
200
0
b b b a
179
12000
10000
8000
6000
4000
2000
0
b c a c
186
2500
2000
1500
1000
500
0
b b b a
172
2000
1500
1000
500
0
b b b a
180
800
600
400
200
0
b a b a
187
10000
8000
6000
4000
2000
0
b c a c
173
400
300
200
100
0
b b b a
181
7000
6000
5000
4000
3000
2000
1000
0
a a a b
188
1000
800
600
400
200
0
b b b a
178
1200
1000
800
600
400
200
0
b bc c a
185
7000
6000
5000
4000
3000
2000
1000
0
b b b a
189
600
500
400
300
200
100
0
b b b a
UP
IP
US
IS

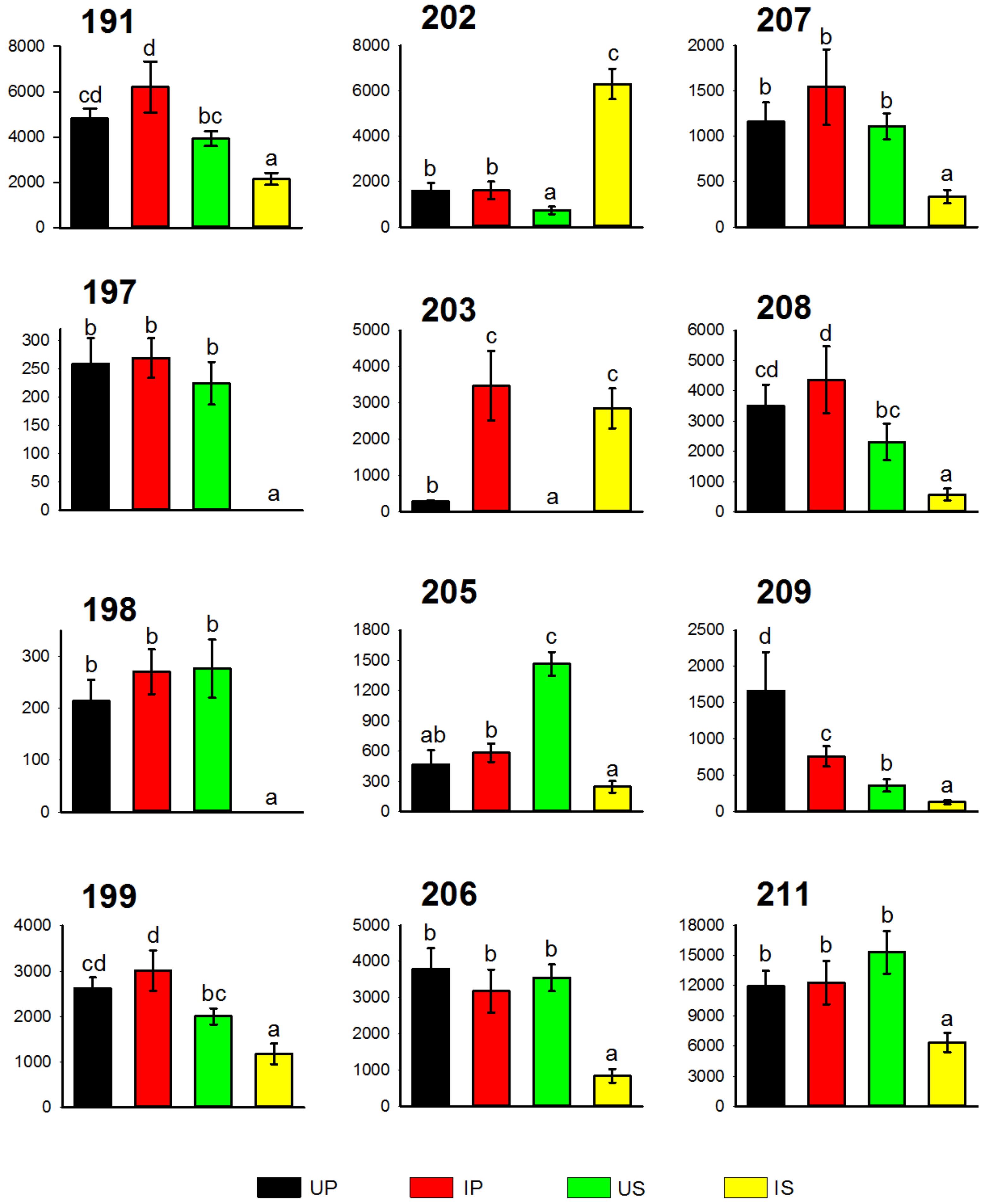

191
202
207
197
203
208
198
205
209
199
206
211
UP
IP
US
IS

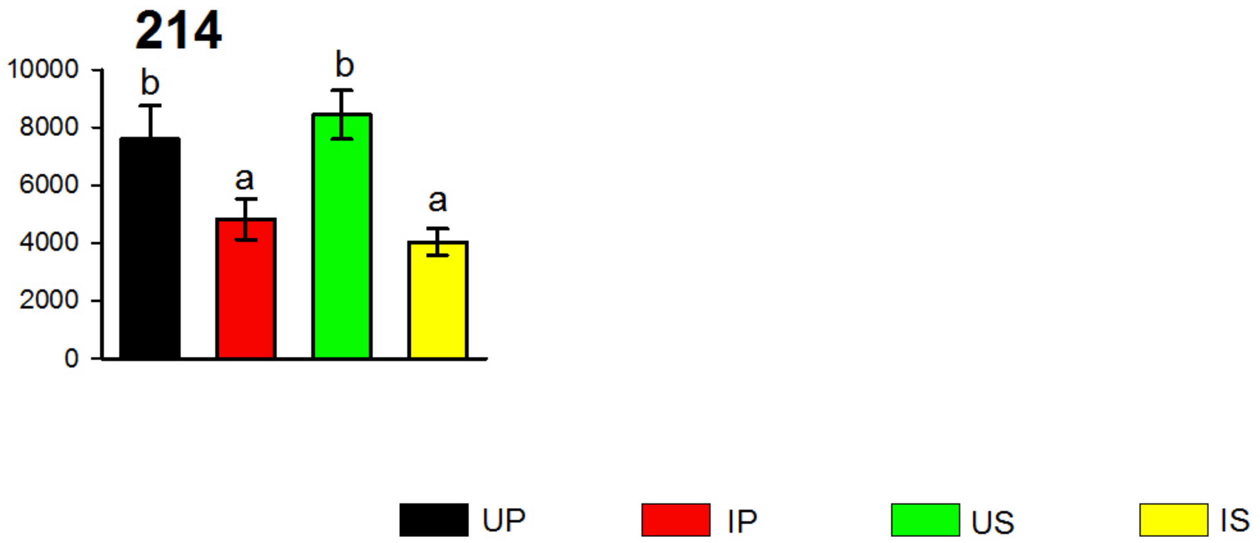
214
10000
8000
6000
4000
2000
0
b
a
b
a
UP
IP
US
IS
