# Supplementary material for: The effect of ‘Candidatus Liberibacter asiaticus’ infection on the proteomic profiles and nutritional status of pre-symptomatic and symptomatic grapefruit (Citrus paradisi) plants
Source: BMC Plant Biol. 2013 Apr 11;13:59. doi: 10.1186/1471-2229-13-59 (PMC3668195; doi:10.1186/1471-2229-13-59)
Supplement: Additional file 7: Appendix S2 — Mascot match results. [file 1471-2229-13-59-S7.pdf]

Supplemental document 1. Mascot match results and the sequences of matched peptides of citrus grapefruit leaf proteins that were differentially produced in response to Las-infection and identified via PMF or MS/MS. The number at the beginning of each paragraph represents the assigned protein spot # as given in Figure 1.

3.
Match to: **clementine0.9_008884m|PAC:19278695** Score: **143** Expect: **6.7e-010**
Nominal mass (M_r): **53950**; Calculated pI value: **6.19**
NCBI BLAST search of clementine0.9_008884m|PAC:19278695 against nr
Unformatted sequence string for pasting into other applications

Fixed modifications: Carbamidomethyl (C)
Variable modifications: Oxidation (M)
Cleavage by Trypsin: cuts C-term side of KR unless next residue is P
Number of mass values matched: **19**
Sequence Coverage: **41%**

Matched peptides shown in **Bold Red**

```
  1 MSCREGLMSP QTETKASVGF KAGVKDYKLT YDTPDYVTKD TDILAAFRVT
 51 PQPGVPPEEA GAAVAAESST GTWTAVWTDG LTSLDRYKGR CYNIEPVAGE
101 ENQYICYVAY PLDLFEEGSV TNMFTSIVGN VFGFKALRAL RLEDLRIPPA
151 YTKTFQGPPH GIQVERDKLN KYGRPLLGCT IKPKLGLFAK NYGRAVYECL
201 RGGLDFTKDD ENVNSQPFMR WRDRFLFCAE ALYKAQAETG EIKGHYLNAT
251 AGTCEEMLKR AVFARELGVP IVMHDYLTGG FTANTTLAHY CRDNGLLLHI
301 HRAMHAVIDR QKNHGMHFRV LAKALRLSGG DHIHAGTVIG KLEGERDITL
351 GFVDLLRDDF VEKDRSRGIY FTQDWVSIPG VIPVAFGGIH VWHMPALTEI
401 FGDDSVLQFG GGTLGHPWGN ALGAVANRVS LEACVQARNE GRDLAREGNE
451 IIREASKWSP ELAAACEVWK SIKFEFAAMD TL
```

Start - End	Observed	Mr(expt)	Mr(calc)	ppm	Miss	Sequence
5 - 21	1825.8200	1824.8127	1824.8928	-44	1	R.EGLMSPQTETKASVGFK.A Oxidation (M)
40 - 48	1021.5310	1020.5237	1020.5240	-0	0	K.DTDILAAFR.V
49 - 86	3824.8520	3823.8447	3823.8541	-2	0	R.VTPQPGVPPEEAGAAVAAESSTGTWTAVWTDGLTSLDR.Y
154 - 166	1465.7580	1464.7507	1464.7474	2	0	K.TFQGPPHGIQVER.D
172 - 184	1502.8520	1501.8447	1501.8439	1	0	K.YGRPLLGCTIKPK.L
195 - 201	910.4460	909.4387	909.4378	1	0	R.AVYECLR.G
202 - 220	2170.0090	2169.0017	2168.9797	10	1	R.GGLDFTKDDENVNSQPFMR.W
202 - 220	2185.9830	2184.9757	2184.9746	1	1	R.GGLDFTKDDENVNSQPFMR.W Oxidation (M)
209 - 220	1451.6350	1450.6277	1450.6147	9	0	K.DDENVNSQPFMR.W
244 - 259	1810.8260	1809.8187	1809.8026	9	0	K.GHYLNATAGTCEEMLK.R Oxidation (M)
244 - 260	1950.9210	1949.9137	1949.9087	3	1	K.GHYLNATAGTCEEMLKR.A
244 - 260	1966.9140	1965.9067	1965.9037	2	1	K.GHYLNATAGTCEEMLKR.A Oxidation (M)
266 - 292	3036.4700	3035.4627	3035.4634	-0	0	R.ELGVPIVMHDYLTGGFTANTTLAHYCR.D
266 - 292	3052.4570	3051.4497	3051.4583	-3	0	R.ELGVPIVMHDYLTGGFTANTTLAHYCR.D Oxidation (M)
293 - 302	1187.6650	1186.6577	1186.6571	1	0	R.DNGLLLHIHR.A
313 - 319	898.4130	897.4057	897.4028	3	0	K.NHGMHFR.V
313 - 319	914.4220	913.4147	913.3977	19	0	K.NHGMHFR.V Oxidation (M)
347 - 357	1261.7170	1260.7097	1260.7078	2	0	R.DITLGFVDLLR.D
458 - 470	1546.7400	1545.7327	1545.7286	3	0	K.WSPELAAACEVWK.S

10.
Match to: **clementine0.9_022034m|PAC:19283330** Score: **46** Expect: **3.8**
Nominal mass (M_r): **22889**; Calculated pI value: **4.78**
NCBI BLAST search of clementine0.9_022034m|PAC:19283330 against nr
Unformatted sequence string for pasting into other applications

Fixed modifications: Carbamidomethyl (C)
Variable modifications: Oxidation (M)
Cleavage by Trypsin: cuts C-term side of KR unless next residue is P
Number of mass values matched: **6**
Sequence Coverage: **35%**

Matched peptides shown in **Bold Red**

```
  1 MEEVDEEPKS QAPGEVGESE IVTEDAAFVR GEPPQDGDGP PKVDSEVEVL
 51 HDKVTKQIIK EGHGQKPSKY STCFLHYRAW AESTRHKFDD TWLEQQPLEM
101 VLGKEKKETT GLAIGVSSMK AGEHALLHVG WELGYGKEGS FSFPNVPPMA
151 DLVYEVVLIG FDETKEGKAR SDMTVEERIG AADRRKMDGN ALFKEEKLEE
201 AMQQP
```

Start - End	Observed	Mr(expt)	Mr(calc)	ppm	Miss	Sequence
86 - 104	2314.1530	2313.1457	2313.1463	-0	1	R.HKFDDTWLEQQPLEMVLGK.E
88 - 104	2065.0160	2064.0087	2063.9874	10	0	K.FDDTWLEQQPLEMVLGK.E Oxidation (M)
88 - 106	2306.1360	2305.1287	2305.1300	-1	1	K.FDDTWLEQQPLEMVLGKEK.K
107 - 120	1437.6920	1436.6847	1436.7545	-49	1	K.KETTGLAIGVSSMK.A Oxidation (M)
138 - 165	3116.5600	3115.5527	3115.5100	14	0	K.EGSFSFPNVPPMADLVYEVVLIGFDETK.E Oxidation (M)
169 - 178	1193.5990	1192.5917	1192.5506	34	1	K.ARSDMTVEER.I

16.
Match to: **clementine0.9_015021m|PAC:19276732** Score: **64** Expect: **0.055**
Nominal mass (M_r): **38020**; Calculated pI value: **4.55**
NCBI BLAST search of clementine0.9_015021m|PAC:19276732 against nr
Unformatted sequence string for pasting into other applications

Fixed modifications: Carbamidomethyl (C)
Variable modifications: Oxidation (M)
Cleavage by Trypsin: cuts C-term side of KR unless next residue is P
Number of mass values matched: **8**
Sequence Coverage: **29%**

Matched peptides shown in **Bold Red**

```
  1 MSSSTASVLK PLSSMADSTS LLSPPSIFAR NPYFSIHPRP RPIKLHLSDS
 51 SLSSKGFSFK LKKTTHFSSF TTFVAQTSDW ADQEEDKDNT TITLEQEQEE
101 NGEEEPNWEN QGADETEGNL SDWGEPEGED TVVEAGERQE ESGEEGVFEE
```

```
151 EEFVEPPEDA KLFVGNLPYD VDSEKLAMLF EKAGTVEIAE VIYNRETDRS
201 RGFGFVTMST VEEAEKAVEM FHRYDIDGRL LTVNKAAPRG TQPERAPRVF
251 EPGFRIYVGN LPWEVDNARL EQVFSEHGKV VNARVVYDRE TGRSRGFGFV
301 TMSSETELND AIAALDGQNL DGRAIRVNVA EDRQRRSSF
```

Start - End	Observed	Mr(expt)	Mr(calc)	ppm	Miss	Sequence	
**162 - 175**	**1595.7820**	**1594.7747**	**1594.7879**	**-8**	**0**	**K.LFVGNLPYDVDSEK.L**	
**183 - 195**	**1434.7520**	**1433.7447**	**1433.7514**	**-5**	**0**	**K.AGTVEIAEVIYNR.E**	
**202 - 216**	**1631.7530**	**1630.7457**	**1630.7549**	**-6**	**0**	**R.GFGFVTMSTVEEAEK.A**	
**249 - 255**	**851.4380**	**850.4307**	**850.4337**	**-4**	**0**	**R.VFEPGFR.I**	
**256 - 269**	**1645.8220**	**1644.8147**	**1644.8260**	**-7**	**0**	**R.IYVGNLPWEVDNAR.L**	
**270 - 279**	**1173.5790**	**1172.5717**	**1172.5826**	**-9**	**0**	**R.LEQVFSEHGK.V**	
**296 - 323**	**2928.3370**	**2927.3297**	**2927.3607**	**-11**	**0**	**R.GFGFVTMSSETELNDAIAALDGQNLDGR.A**	
**296 - 323**	**2944.3370**	**2943.3297**	**2943.3556**	**-9**	**0**	**R.GFGFVTMSSETELNDAIAALDGQNLDGR.A**	Oxidation (M)

19.
Match to: **clementine0.9_015646m|PAC:19271726** Score: **64** Expect: **0.05**
Nominal mass (M_r): **36735**; Calculated pI value: **4.81**
NCBI BLAST search of clementine0.9_015646m|PAC:19271726 against nr
Unformatted sequence string for pasting into other applications

Fixed modifications: Carbamidomethyl (C)
Variable modifications: Oxidation (M)
Cleavage by Trypsin: cuts C-term side of KR unless next residue is P

Number of mass values matched: **8**
Sequence Coverage: **24%**

Matched peptides shown in **Bold Red**

```
  1 MRLIGSLLIF SLVLSFVLGG SAGNCGSGVV CPGGECCSRF GWCGLTTDYC
 51 CEGCQSNCNQ VVCGECDPDD GTAGDGGELG KIISRKMFED LLEYRNDKRC
101 PARCFYTYDA FIEAAKAFPA FGNSGNETMR KREIAAFFAQ TGHETTGGWP
151 DAPGGEYAWG YCFNREVGAA SSDYCDPNYP CRGKYYGRGP IQLSWNYNYL
201 RCGEGLGLGE ELLNNPDLLA TDPVLSFKSA IWFWMTAQPP KPSCHEVIID
251 EWKPSANDVN AGRLPGYGLT TNIINGGIEC GYVGNDAVRN RIGFFTTFCG
301 KFGIQPGDNL DCSNQRPYGL NLMAQSM
```

Start - End	Observed	Mr(expt)	Mr(calc)	ppm	Miss	Sequence	
**86 - 95**	**1343.7170**	**1342.7097**	**1342.6591**	**38**	**1**	**R.KMFEDLLEYR.N**	
**86 - 95**	**1359.7110**	**1358.7037**	**1358.6540**	**37**	**1**	**R.KMFEDLLEYR.N**	Oxidation (M)
**87 - 95**	**1215.6190**	**1214.6117**	**1214.5641**	**39**	**0**	**K.MFEDLLEYR.N**	
**87 - 95**	**1231.6200**	**1230.6127**	**1230.5591**	**44**	**0**	**K.MFEDLLEYR.N**	Oxidation (M)
**104 - 116**	**1598.7790**	**1597.7717**	**1597.7123**	**37**	**0**	**R.CFYTYDAFIEAAK.A**	
**166 - 182**	**1960.8450**	**1959.8377**	**1959.7727**	**33**	**0**	**R.EVGAASSDYCDPNYPCR.G**	
**189 - 201**	**1623.8820**	**1622.8747**	**1622.8205**	**33**	**0**	**R.GPIQLSWNYNYLR.C**	
**202 - 228**	**2871.4970**	**2870.4897**	**2870.4372**	**18**	**0**	**R.CGEGLGLGEELLNNPDLLATDPVLSFK.S**	

20.
Match to: **orange1.1g027595m|PAC:18106494** Score: **67** Expect: **0.027**
Nominal mass (M_r): **24749**; Calculated pI value: **4.61**
NCBI BLAST search of orange1.1g027595m|PAC:18106494 against nr
Unformatted sequence string for pasting into other applications

Fixed modifications: Carbamidomethyl (C)
Variable modifications: Oxidation (M)
Cleavage by Trypsin: cuts C-term side of KR unless next residue is P
Number of mass values matched: **8**
Sequence Coverage: **47%**

Matched peptides shown in **Bold Red**

1 METLQSYKEA LASNDDTKAA EIEALLK**SFE DEKIDLER**KV **VNLSEELSAE**
51 **R**ARILR**ISAD FDNFR**KRTEK ER**LSLVTNAQ GEVMERLLQV LDNFER**AKTQ
101 IKVQTEGEEK INNSYQSIYK **QLVEILGSLG VVPVETVGNP FDPLLHEAIM**
151 **REDSTEFDEG VIIEEFRK**GF KLGDRLLRPS MVKVSAGPGP AKPKEEQPSE
201 GEAAVVETAD SSTEEVEAES S

Start - End	Observed	Mr(expt)	Mr(calc)	ppm	Miss	Sequence
28 - 38	1380.6550	1379.6477	1379.6568	-7	1	K.SFEDEKIDLER.K
40 - 51	1345.6740	1344.6667	1344.6885	-16	0	K.VVNLSEELSAER.A
57 - 65	1084.4920	1083.4847	1083.4985	-13	0	R.ISADFDNFR.K
73 - 86	1562.7620	1561.7547	1561.7770	-14	0	R.LSLVTNAQGEVMER.L Oxidation (M)
87 - 96	1246.6670	1245.6597	1245.6717	-10	0	R.LLQVLDNFER.A
121 - 151	3358.7200	3357.7127	3357.8007	-26	0	K.QLVEILGSLGVVPVETVGNPFDPLLHEAIMR.E Oxidation (M)
152 - 167	1914.8470	1913.8397	1913.8531	-7	0	R.EDSTEFDEGVIIEEFR.K
152 - 168	2042.9410	2041.9337	2041.9480	-7	1	R.EDSTEFDEGVIIEEFRK.G

28.
Match to: **clementine0.9_008884m|PAC:19278695** Score: **94** Expect: **5.2e-005**
Nominal mass (M_r): **53950**; Calculated pI value: **6.19**
NCBI BLAST search of clementine0.9_008884m|PAC:19278695 against nr
Unformatted sequence string for pasting into other applications

Fixed modifications: Carbamidomethyl (C)
Variable modifications: Oxidation (M)
Cleavage by Trypsin: cuts C-term side of KR unless next residue is P
Number of mass values matched: **14**
Sequence Coverage: **36%**

Matched peptides shown in **Bold Red**

1 MSCREGLMSP QTETKASVGF KAGVKDYKLT YDTPDYVTK**D TDILAAFRVT**
51 **PQPGVPPEEA GAAVAAESST GTWTAVWTDG LTSLDR**YKGR CYNIEPVAGE
101 ENQYICYVAY PLDLFEEGSV TNMFTSIVGN VFGFKALRAL RLEDLRIPPA
151 YTK**TFQGPPH GIQVER**DKLN K**YGRPLLGCT IKPK**LGLFAK NYGR**AVYECL**
201 **RGGLDFTKDD ENVNSQPFMR** WRDRFLFCAE ALYKAQAETG EIK**GHYLNAT**
251 **AGTCEEMLKR** AVFAR**ELGVP IVMHDYLTGG FTANTTLAHY CRDNGLLLHI**
301 **HR**AMHAVIDR QKNHGMHFRV LAKALRLSGG DHIHAGTVIG KLEGER**DITL**
351 **GFVDLLR**DDF VEKDRSRGIY FTQDWVSIPG VIPVAFGGIH VWHMPALTEI
401 FGDDSVLQFG GGTLGHPWGN ALGAVANRVS LEACVQARNE GRDLAREGNE
451 IIREASK**WSP ELAAACEVWK** SIKFEFAAMD TL

Start - End	Observed	Mr(expt)	Mr(calc)	ppm	Miss	Sequence	
40 - 48	1021.5120	1020.5047	1020.5240	-19	0	K.DTDILAAFR.V	
49 - 86	3824.8040	3823.7967	3823.8541	-15	0	R.VTPQPGVPPEEAGAAVAAESSTGTWTAVWTDGLTSLDR.Y	
154 - 166	1465.7410	1464.7337	1464.7474	-9	0	K.TFQGPPHGIQVER.D	
172 - 184	1502.8300	1501.8227	1501.8439	-14	0	K.YGRPLLGCTIKPK.L	
195 - 201	910.4400	909.4327	909.4378	-6	0	R.AVYECLR.G	
202 - 220	2169.9900	2168.9827	2168.9797	1	1	R.GGLDFTKDDENVNSQPFMR.W	
202 - 220	2185.9820	2184.9747	2184.9746	0	1	R.GGLDFTKDDENVNSQPFMR.W	Oxidation (M)
209 - 220	1451.6500	1450.6427	1450.6147	19	0	K.DDENVNSQPFMR.W	
244 - 260	1966.8950	1965.8877	1965.9037	-8	1	K.GHYLNATAGTCEEMLKR.A	Oxidation (M)
266 - 292	3036.4110	3035.4037	3035.4634	-20	0	R.ELGVPIVMHDYLTGGFTANTTLAHYCR.D	
266 - 292	3052.4460	3051.4387	3051.4583	-6	0	R.ELGVPIVMHDYLTGGFTANTTLAHYCR.D	Oxidation (M)
293 - 302	1187.6450	1186.6377	1186.6571	-16	0	R.DNGLLLHIHR.A	
347 - 357	1261.7020	1260.6947	1260.7078	-10	0	R.DITLGFVDLLR.D	
458 - 470	1546.7260	1545.7187	1545.7286	-6	0	K.WSPELAAACEVWK.S	

30.
Match to: **clementine0.9_004316m|PAC:19277375** Score: **96** Expect: **3.8e-005**
Nominal mass (M_r): **73678**; Calculated pI value: **5.10**
NCBI BLAST search of clementine0.9_004316m|PAC:19277375 against nr
Unformatted sequence string for pasting into other applications

Fixed modifications: Carbamidomethyl (C)
Variable modifications: Oxidation (M)
Cleavage by Trypsin: cuts C-term side of KR unless next residue is P
Number of mass values matched: **13**
Sequence Coverage: **22%**

Matched peptides shown in **Bold Red**

1 MAGSWRARGS LVVLAIVFFG GLFAISIAKE EATKLGTVIG IDLGTTYSCV
51 GVYK**NGHVEI IANDQGNRIT PSWVAFTDSE R**LIGEAAKNQ AAVNPDRTIF
101 DVKRLIGRKF EDKEVQRDMK LAPYKIVNR**D GKPYIQVQIR** DGETKVFSPE
151 EISAMILTKM KETAEAFLGK K**IKDAVVTVP AYFNDAQR**QA TKDAGIIAGL

201 NVARIINEPT AAAIAYGLDK KGGEKNILVF DLGGGTFDVS ILTIDNGVFE
251 VLSTNGDTHL GGEDFDQRVM EYFIKLIKKK HGKDISKDKR AIGKLRREAE
301 RAKRALSSQH QVR**VEIESLF DGIDFSEPLT RARFEELNND LFR**KTMGPVK
351 KAMEDAGLEK **NQIDEIVLVG GSTR**IPKVQQ LLKDYFDGKE PNKGVNPDEA
401 VAYGAAVQGG ILSGEGGDET KDILLLDVAP LTLGIETVGG VMTKLIPRNT
451 VIPTKK**SQVF TTYQDQQTTV SIQVFEGER**S LTKDCRLLGK **FDLSGIPPAP**
501 **R**GTPQIEVTF EVDANGILNV KAEDKGTGKS EKITITNDKG RLSQEEIERM
551 VREAEEFAEE DKKVKEKIDA RNSLETYVYN MKNQINDKDK LADKLESDEK
601 DKIETAVKEA LEWLDDNQSA EKEDYEEK**LK EVEAVCNPII TAVYQR**SGGA
651 PGAGTESSDD DDSHDEL

Start - End	Observed	Mr(expt)	Mr(calc)	ppm	Miss	Sequence
55 - 68	1536.7230	1535.7157	1535.7440	-18	0	K.NGHVEIIANDQGNR.I
69 - 81	1508.7260	1507.7187	1507.7307	-8	0	R.ITPSWVAFTDSER.L
130 - 140	1316.7150	1315.7077	1315.7248	-13	0	R.DGKPYIQVQIR.D
172 - 188	1906.9610	1905.9537	1905.9949	-22	1	K.IKDAVVTVPAYFNDAQR.Q
174 - 188	1665.7930	1664.7857	1664.8158	-18	0	K.DAVVTVPAYFNDAQR.Q
314 - 331	2067.0030	2065.9957	2066.0208	-12	0	R.VEIESLFDGIDFSEPLTR.A
332 - 343	1523.7500	1522.7427	1522.7528	-7	1	R.ARFEELNNDLFR.K
334 - 343	1296.6100	1295.6027	1295.6146	-9	0	R.FEELNNDLFR.K
361 - 374	1500.7860	1499.7787	1499.7944	-10	0	K.NQIDEIVLVGGSTR.I
457 - 479	2691.2660	2690.2587	2690.2824	-9	0	K.SQVFTTYQDQQTTVSIQVFEGER.S
491 - 501	1169.6270	1168.6197	1168.6241	-4	0	K.FDLSGIPPAPR.G
629 - 646	2103.0840	2102.0767	2102.1194	-20	1	K.LKEVEAVCNPIITAVYQR.S
631 - 646	1861.9240	1860.9167	1860.9404	-13	0	K.EVEAVCNPIITAVYQR.S

39.
Match to: **clementine0.9_018696m|PAC:19273944** Score: **72** Expect: **0.0076**

Nominal mass (M_r): **29272**; Calculated pI value: **5.10**
NCBI BLAST search of clementine0.9_018696m|PAC:19273944 against nr
Unformatted sequence string for pasting into other applications

Fixed modifications: Carbamidomethyl (C)
Variable modifications: Oxidation (M)
Cleavage by Trypsin: cuts C-term side of KR unless next residue is P
Number of mass values matched: **7**
Sequence Coverage: **32%**

Matched peptides shown in **Bold Red**

1 MIFLLLLSVF LRGASSSILS EDTPISFSFP SFAKDSCDNK **TLICYGAIES**
51 **SGALSITPGP PPNLPIR**KVG R**VLYGKPLSL QR**SFIDTTIT IKISRHQNYT
101 DR**AGDGMTFI FASDKNGPSA KGVGEYLGLQ SSPGDKFPPL AVELDTCLNK**
151 NLNDPDDNHI GIDINGIESN PVNSLLDVDL KSGRAIQVRI YYNPDFGQLS
201 VYAAYSGETL VKVVEKPINL SDIIPTPVYV GFTAATGDFL ESHEVINWTF

251 NSFPVPPSLK EKNLVMPI

Start - End	Observed	Mr(expt)	Mr(calc)	ppm	Miss	Sequence
**41 - 67**	**2794.4570**	**2793.4497**	**2793.4735**	**-9**	**0**	**K.TLICYGAIESSGALSITPGPPPNLPIR.K**
**72 - 82**	**1273.7510**	**1272.7437**	**1272.7554**	**-9**	**0**	**R.VLYGKPLSLQR.S**
**103 - 115**	**1375.6120**	**1374.6047**	**1374.6126**	**-6**	**0**	**R.AGDGMTFIFASDK.N** Oxidation (M)
**103 - 121**	**1929.8700**	**1928.8627**	**1928.8938**	**-16**	**1**	**R.AGDGMTFIFASDKNGPSAK.G** Oxidation (M)
**122 - 136**	**1506.7250**	**1505.7177**	**1505.7362**	**-12**	**0**	**K.GVGEYLGLQSSPGDK.F**
**122 - 150**	**3104.5200**	**3103.5127**	**3103.5536**	**-13**	**1**	**K.GVGEYLGLQSSPGDKFPPLAVELDTCLNK.N**
**137 - 150**	**1616.8070**	**1615.7997**	**1615.8280**	**-17**	**0**	**K.FPPLAVELDTCLNK.N**

41.
Match to: **clementine0.9_017467m|PAC:19271755** Score: 53 Expect: **11**

Nominal mass (M_r): **32459**; Calculated pI value: **5.06**
NCBI BLAST search of clementine0.9_017467m|PAC:19271755 against nr
Unformatted sequence string for pasting into other applications

Fixed modifications: Carbamidomethyl (C)
Variable modifications: Oxidation (M)
Cleavage by Trypsin: cuts C-term side of KR unless next residue is P
Number of mass values matched: **5**
Sequence Coverage: **20%**

Matched peptides shown in **Bold Red**

1 MRLIGSLLIF SLVLSFVLGG SAQNCGSGVV YGGRDTGHGT HGGELGKIIS
51 R**EMFDDLLEY R**NDERCPARG FYTYDAFIEA AQAFPGFGNS GNETMRKREI
101 AAFFAQTGHE TTGGWPDAPG GEYAWGYCFI SEVSPPSDYC DPNYPCRGKY
151 YGR**GPIQLSW NYNYLRCGEG LGLGEELLNN PDLLATDPVL SFK**SAIWFWM
201 TAQPPKPSCH EVIIDEWKPS ANDVNAGRLP GYGLTTNIIN GGIECGQGGN
251 AAVRNR**IGFF TTFCGK**FGIQ PGDSLDCYNQ RPYGLNLMAQ SM

Start - End	Observed	Mr(expt)	Mr(calc)	ppm	Miss	Sequence
**52 - 61**	**1330.5880**	**1329.5807**	**1329.5911**	**-8**	**0**	**R.EMFDDLLEYR.N**
**52 - 61**	**1346.5880**	**1345.5807**	**1345.5860**	**-4**	**0**	**R.EMFDDLLEYR.N** Oxidation (M)
**154 - 166**	**1623.8150**	**1622.8077**	**1622.8205**	**-8**	**0**	**R.GPIQLSWNYNYLR.C**
**167 - 193**	**2871.4060**	**2870.3987**	**2870.4372**	**-13**	**0**	**R.CGEGLGLGEELLNNPDLLATDPVLSFK.S**
**257 - 266**	**1177.5580**	**1176.5507**	**1176.5638**	**-11**	**0**	**R.IGFFTTFCGK.F**

43.
Match to: **clementine0.9_015646m|PAC:19271726** Score: **73** Expect: **0.0064**

Nominal mass (M_r): **36735**; Calculated pI value: **4.81**
NCBI BLAST search of clementine0.9_015646m|PAC:19271726 against nr
Unformatted sequence string for pasting into other applications

Fixed modifications: Carbamidomethyl (C)
Variable modifications: Oxidation (M)
Cleavage by Trypsin: cuts C-term side of KR unless next residue is P
Number of mass values matched: **11**
Sequence Coverage: **41%**

Matched peptides shown in **Bold Red**

```
  1 MRLIGSLLIF SLVLSFVLGG SAGNCGSGVV CPGGECCSRF GWCGLTTDYC
 51 CEGCQSNCNQ VVCGECDPDD GTAGDGGELG KIISRKMFED LLEYRNDKRC
101 PARCFYTYDA FIEAAKAFPA FGNSGNETMR KREIAAFFAQ TGHETTGGWP
151 DAPGGEYAWG YCFNREVGAA SSDYCDPNYP CRGKYYGRGP IQLSWNYNYL
201 RCGEGLGLGE ELLNNPDLLA TDPVLSFKSA IWFWMTAQPP KPSCHEVIID
251 EWKPSANDVN AGRLPGYGLT TNIINGGIEC GYVGNDAVRN RIGFFTTFCG
301 KFGIQPGDNL DCSNQRPYGL NLMAQSM
```

Start - End	Observed	Mr(expt)	Mr(calc)	ppm	Miss	Sequence
86 - 95	1343.6510	1342.6437	1342.6591	-11	1	R.KMFEDLLEYR.N
86 - 95	1359.6460	1358.6387	1358.6540	-11	1	R.KMFEDLLEYR.N Oxidation (M)
87 - 95	1215.5620	1214.5547	1214.5641	-8	0	K.MFEDLLEYR.N
87 - 95	1231.5610	1230.5537	1230.5591	-4	0	K.MFEDLLEYR.N Oxidation (M)
104 - 116	1598.7050	1597.6977	1597.7123	-9	0	R.CFYTYDAFIEAAK.A
117 - 130	1498.6510	1497.6437	1497.6671	-16	0	K.AFPAFGNSGNETMR.K
133 - 165	3663.5590	3662.5517	3662.5950	-12	0	R.EIAAFFAQTGHETTGGWPDAPGGEYAWGYCFNR.E
166 - 182	1960.7670	1959.7597	1959.7727	-7	0	R.EVGAASSDYCDPNYPCR.G
189 - 201	1623.8150	1622.8077	1622.8205	-8	0	R.GPIQLSWNYNYLR.C
202 - 228	2871.3940	2870.3867	2870.4372	-18	0	R.CGEGLGLGEELLNNPDLLATDPVLSFK.S
292 - 301	1177.5520	1176.5447	1176.5638	-16	0	R.IGFFTTFCGK.F

44.
Match to: **orange1.1g024380m|PAC:18096978** Score: **85** Expect: **0.00047**
Nominal mass (M_r): **29300**; Calculated pI value: **5.10**
NCBI BLAST search of orange1.1g024380m|PAC:18096978 against nr
Unformatted sequence string for pasting into other applications

Fixed modifications: Carbamidomethyl (C)
Variable modifications: Oxidation (M)

Cleavage by Trypsin: cuts C-term side of KR unless next residue is P
Number of mass values matched: **7**
Sequence Coverage: **38%**

Matched peptides shown in **Bold Red**

```
  1 MIFLLLLSVF LRGASSSILS EDTPISFSFP SFAKDSCDNK TLICYGAIES
 51 SGALSITPGP PPNLPIRKVG RVLYGKPLSL QRSFIDTTIT IKISRHQNYT
101 DRAGDGMTFI FASDKNGPSA KGVGEYLGLQ SSPGDKFPPL AVELDTCLNK
151 NLNDPDDNHI GIDINGIESN PVNSLLDVDL KSGRAIQVRI YYNPDFGQLS
201 IYAAYSGETL VKVIEKPINL SDIIPTPVYV GFTAATGDFL ESHEVINWTF
251 NSFPVPPSLK EKNLVMPI
```

Start - End	Observed	Mr(expt)	Mr(calc)	ppm	Miss	Sequence
41 - 67	2794.4560	2793.4487	2793.4735	-9	0	K.TLICYGAIESSGALSITPGPPPNLPIR.K
72 - 82	1273.7470	1272.7397	1272.7554	-12	0	R.VLYGKPLSLQR.S
103 - 115	1375.6170	1374.6097	1374.6126	-2	0	R.AGDGMTFIFASDK.N Oxidation (M)
122 - 136	1506.7270	1505.7197	1505.7362	-11	0	K.GVGEYLGLQSSPGDK.F
122 - 150	3104.5220	3103.5147	3103.5536	-13	1	K.GVGEYLGLQSSPGDKFPPLAVELDTCLNK.N
137 - 150	1616.8160	1615.8087	1615.8280	-12	0	K.FPPLAVELDTCLNK.N
190 - 212	2612.2600	2611.2527	2611.2846	-12	0	R.IYYNPDFGQLSIYAAYSGETLVK.V

45.
Match to: **clementine0.9_010542m|PAC:19255809** Score: **99** Expect: **1.6e-005**
Nominal mass (M_r): **48362**; Calculated pI value: **5.04**
NCBI BLAST search of clementine0.9_010542m|PAC:19255809 against nr
Unformatted sequence string for pasting into other applications

Fixed modifications: Carbamidomethyl (C)
Variable modifications: Oxidation (M)
Cleavage by Trypsin: cuts C-term side of KR unless next residue is P
Number of mass values matched: **14**
Sequence Coverage: **36%**

Matched peptides shown in **Bold Red**

```
  1 MAAIISCNFV TPRLPTNRIQ TTHYKCNNNN GRVLSRRLLP KCCVKNHNHN
 51 PFQKLKECAI SIALAAGLIT GVPAIADANI NANINMAMPD VSVLISGPPI
101 KDPGALLRYA LPIDNKAVRE VQKPLEDITD SLKIAGVKAL DPVERNVRQA
151 SRTLKQGKSL IVEGLAESKK EHGMELLQKL EAGMDELQQI VEDRDRDAVA
201 PKQKELLNYV GGVEEDMVDG FPYEVPEEYQ SMPLLKGRAT VDMKVKVKDN
251 PNVDECVFRI VLDGYNAPVT AGNFVDLVQR HFYDGMEIQR ADGFVVQTGD
301 PEGPAEGFID PSTEKTRTIP LEIMVEGEKS PFYGATLEEL GLYKAQTKLP
351 FNAFGTMAMA RDEFEDNSGS SQVFWLLKES ELTPSNANIL DGRYAVFGYV
401 TENEGLLADV KVGDVIQSIQ VVSGLENLVN PSYKIAA
```

Start - End	Observed	Mr(expt)	Mr(calc)	ppm	Miss	Sequence
**180 - 194**	**1745.8210**	**1744.8137**	**1744.8301**	**-9**	**0**	**K.LEAGMDELQQIVEDR.D**
**180 - 194**	**1761.8150**	**1760.8077**	**1760.8251**	**-10**	**0**	**K.LEAGMDELQQIVEDR.D** Oxidation (M)
**247 - 259**	**1591.7360**	**1590.7287**	**1590.7460**	**-11**	**1**	**K.VKDNPNVDECVFR.I**
**249 - 259**	**1364.6110**	**1363.6037**	**1363.5827**	**15**	**0**	**K.DNPNVDECVFR.I**
**281 - 290**	**1295.5820**	**1294.5747**	**1294.5764**	**-1**	**0**	**R.HFYDGMEIQR.A**
**281 - 290**	**1311.5660**	**1310.5587**	**1310.5714**	**-10**	**0**	**R.HFYDGMEIQR.A** Oxidation (M)
**291 - 315**	**2563.1480**	**2562.1407**	**2562.1762**	**-14**	**0**	**R.ADGFVVQTGDPEGPAEGFIDPSTEK.T**
**318 - 344**	**3027.4490**	**3026.4417**	**3026.5562**	**-38**	**1**	**R.TIPLEIMVEGEKSPFYGATLEELGLYK.A**
**330 - 344**	**1687.8340**	**1686.8267**	**1686.8505**	**-14**	**0**	**K.SPFYGATLEELGLYK.A**
**349 - 361**	**1442.6700**	**1441.6627**	**1441.6846**	**-15**	**0**	**K.LPFNAFGTMAMAR.D** Oxidation (M)
**349 - 361**	**1458.6750**	**1457.6677**	**1457.6795**	**-8**	**0**	**K.LPFNAFGTMAMAR.D** 2 Oxidation (M)
**379 - 393**	**1615.7840**	**1614.7767**	**1614.7849**	**-5**	**0**	**K.ESELTPSNANILDGR.Y**
**394 - 411**	**1987.9960**	**1986.9887**	**1986.9939**	**-3**	**0**	**R.YAVFGYVTENEGLLADVK.V**
**412 - 434**	**2458.2630**	**2457.2557**	**2457.3115**	**-23**	**0**	**K.VGDVIQSIQVVSGLENLVNPSYK.I**

49.
Match to: **orange1.1g012383m|PAC:18138110** Score: **150** Expect: **1.3e-010**
Nominal mass (M_r): **51251**; Calculated pI value: **5.33**
NCBI BLAST search of orange1.1g012383m|PAC:18138110 against nr
Unformatted sequence string for pasting into other applications

Fixed modifications: Carbamidomethyl (C)
Variable modifications: Oxidation (M)
Cleavage by Trypsin: cuts C-term side of KR unless next residue is P
Number of mass values matched: **24**
Sequence Coverage: **56%**

Matched peptides shown in **Bold Red**

```
  1 MAAAVPLSFN GSGAATSVPS SSFFGTSLKK VSSRIPPSKV PSASFKITAE
 51 VDENKQTKKD RWKGLAYDES DDQQDITRGK GAVDSLFQAP MGTGTHYAVM
101 SSYDYISQGL RTYNLDNTID GLYIAPAFMD KLVVHITKNF MSLPNIKVPL
151 ILGIWGGKGQ GKSFQCELVF AKMGINPIMM SAGELESGNA GEPAKLIRQR
201 YREAADIIKK GKMCCLMIND LDAGAGRMGG TTQYTVNNQM VNATLMNIAD
251 NPTCVQLPGM YNKEENPRVP IIVTGNDFST LYAPLIRDGR MEKFYWAPTR
301 EDRIGVCKGI FRNDNVADDD IVKLVDTFPG QSIDFFGALR ARVYDDEVRK
351 WISGVGVGSI GKSLVNSKEA APTFEQPRMT MEKLLEYGNM IVQEQENVKR
401 VQLADKYLSE AALGEANEDA IQSGNFYGKA AQQMNVPVPE GCTDPTAENF
451 DPTARSDDGS CQYTL
```

Start - End	Observed	Mr(expt)	Mr(calc)	ppm	Miss	Sequence
**64 - 78**	**1725.7450**	**1724.7377**	**1724.7489**	**-6**	**0**	**K.GLAYDESDDQQDITR.G**
**81 - 111**	**3322.5280**	**3321.5207**	**3321.5435**	**-7**	**0**	**K.GAVDSLFQAPMGTGTHYAVMSSYDYISQGLR.T**
**81 - 111**	**3338.5590**	**3337.5517**	**3337.5384**	**4**	**0**	**K.GAVDSLFQAPMGTGTHYAVMSSYDYISQGLR.T** Oxidation (M)
**81 - 111**	**3354.5120**	**3353.5047**	**3353.5333**	**-9**	**0**	**K.GAVDSLFQAPMGTGTHYAVMSSYDYISQGLR.T** 2 Oxidation (M)

**112 - 131**	**2275.0710**	**2274.0637**	**2274.0878**	**-11**	**0**	**R.TYNLDNTIDGLYIAPAFMDK.L**	
**112 - 131**	**2291.0510**	**2290.0437**	**2290.0827**	**-17**	**0**	**R.TYNLDNTIDGLYIAPAFMDK.L**	Oxidation (M)
**148 - 158**	**1152.6990**	**1151.6917**	**1151.7067**	**-13**	**0**	**K.VPLILGIWGGK.G**	
**163 - 172**	**1228.5930**	**1227.5857**	**1227.5958**	**-8**	**0**	**K.SFQCELVFAK.M**	
**173 - 195**	**2304.0570**	**2303.0497**	**2303.0595**	**-4**	**0**	**K.MGINPIMMSAGELESGNAGEPAK.L**	
**173 - 195**	**2320.0510**	**2319.0437**	**2319.0545**	**-5**	**0**	**K.MGINPIMMSAGELESGNAGEPAK.L**	Oxidation (M)
**173 - 195**	**2336.0370**	**2335.0297**	**2335.0494**	**-8**	**0**	**K.MGINPIMMSAGELESGNAGEPAK.L**	2 Oxidation (M)
**173 - 195**	**2352.0420**	**2351.0347**	**2351.0443**	**-4**	**0**	**K.MGINPIMMSAGELESGNAGEPAK.L**	3 Oxidation (M)
**213 - 227**	**1696.7130**	**1695.7057**	**1695.7201**	**-8**	**0**	**K.MCCLMINDLDAGAGR.M**	
**213 - 227**	**1712.7090**	**1711.7017**	**1711.7150**	**-8**	**0**	**K.MCCLMINDLDAGAGR.M**	Oxidation (M)
**228 - 263**	**3989.7320**	**3988.7247**	**3988.8250**	**-25**	**0**	**R.MGGTTQYTVNNQMVNATLMNIADNPTCVQLPGMYNK.E**	
**269 - 287**	**2089.1570**	**2088.1497**	**2088.1619**	**-6**	**0**	**R.VPIIVTGNDFSTLYAPLIR.D**	
**294 - 300**	**940.4620**	**939.4547**	**939.4603**	**-6**	**0**	**K.FYWAPTR.E**	
**324 - 340**	**1882.9580**	**1881.9507**	**1881.9625**	**-6**	**0**	**K.LVDTFPGQSIDFFGALR.A**	
**343 - 349**	**895.4100**	**894.4027**	**894.4083**	**-6**	**0**	**R.VYDDEVR.K**	
**369 - 378**	**1145.5510**	**1144.5437**	**1144.5513**	**-7**	**0**	**K.EAAPTFEQPR.M**	
**384 - 400**	**2063.0380**	**2062.0307**	**2062.0517**	**-10**	**1**	**K.LLEYGNMIVQEQENVKR.V**	
**384 - 400**	**2079.0320**	**2078.0247**	**2078.0466**	**-11**	**1**	**K.LLEYGNMIVQEQENVKR.V**	Oxidation (M)
**430 - 455**	**2816.2420**	**2815.2347**	**2815.2542**	**-7**	**0**	**K.AAQQMNVPVPEGCTDPTAENFDPTAR.S**	
**430 - 455**	**2832.2380**	**2831.2307**	**2831.2491**	**-6**	**0**	**K.AAQQMNVPVPEGCTDPTAENFDPTAR.S**	Oxidation (M)

57.
Match to: **clementine0.9_003662m|PAC:19267826** Score: **168** Expect: **2.1e-012**
Nominal mass (M_r): **76759**; Calculated pI value: **5.31**
NCBI BLAST search of clementine0.9_003662m|PAC:19267826 against nr
Unformatted sequence string for pasting into other applications

Fixed modifications: Carbamidomethyl (C)
Variable modifications: Oxidation (M)
Cleavage by Trypsin: cuts C-term side of KR unless next residue is P
Number of mass values matched: **22**
Sequence Coverage: **35%**

Matched peptides shown in **Bold Red**

1 **MACSSAAQIH FLGNISFSSR K**NSKTGRENT AGSRNLFFGQ RVGGNRFGSV
51 PSAAFLRLKS ENRGRRYVGP VRVVNEK**VVG IDLGTTNSAV AAMEGGKPTI**
101 **VTNAEGQR**TT PSVVAYTKNG DRLVGQIAKR **QAVVNPENTF FSVKR**FIGRK
151 MVEVDEESKQ VSYRVVRDEN GNVKLECPAI GK**QFAAEEIS AQVLR**KLVDD
201 ASKFLNDSVT K**AVVTVPAYF NDSQR**TATKD AGR**IAGLDVL RIINEPTAAS**
251 **LAYGFEK**KNN ETILVFDLGG GTFDVSVLEV GDGVFEVLST SGDTHLGGDD
301 FDKR**IVDWLA SNFK**RDEGID LLKDKQALQR LTETAEKAK**M ELSSLTQTNI**
351 **SLPFITATAD GPK**HIDTTLT R**VKFEELCSD LLDR**LKTPVE TSLRDAKLSF
401 K**DLDEVILVG GSTR**IPAVQE LVKKMTGREP NVTVNPDEVV ALGAAVQAGV
451 LAGDVSDIVL LDVTPLSLGL ETLGGVMTKI IPRNTTLPTS K**SEVFSTAAD**

501 **GQTSVEINVL** **QGER**EFVRDN KSLGSFR**LDG** **IPPAPR**GVPQ IEVKFDIDAN
551 GILSVSAVDK GTGK**KQDITI** **TGASTLPNDE** **VQRMVQEAER** FAKEDKEKR**D**
601 **AIDTKNQADS** **VVYQTEK**QLK ELGDKVPAEV KGKVEGKLKE LKDAIAEGST
651 QAMKDTMSAL NQEVMQLGQS LYNQPGAGAA PGAGPTPGAE AGASDSSNKG
701 QDGDVIDADF TDSK

Start - End	Observed	Mr(expt)	Mr(calc)	ppm	Miss	Sequence
1 - 21	2328.1120	2327.1047	2327.1151	-4	1	-.MACSSAAQIHFLGNISFSSRK.N Oxidation (M)
78 - 108	3056.5270	3055.5197	3055.5608	-13	0	K.VVGIDLGTTNSAVAAMEGGKPTIVTNAEGQR.T
78 - 108	3072.5290	3071.5217	3071.5557	-11	0	K.VVGIDLGTTNSAVAAMEGGKPTIVTNAEGQR.T Oxidation (M)
131 - 144	1579.7910	1578.7837	1578.8042	-13	0	R.QAVVNPENTFFSVK.R
131 - 145	1735.8550	1734.8477	1734.9053	-33	1	R.QAVVNPENTFFSVKR.F
183 - 195	1461.7600	1460.7527	1460.7623	-7	0	K.QFAAEEISAQVLR.K
212 - 225	1566.7790	1565.7717	1565.7838	-8	0	K.AVVTVPAYFNDSQR.T
234 - 241	856.5120	855.5047	855.5178	-15	0	R.IAGLDVLR.I
242 - 257	1723.8730	1722.8657	1722.8828	-10	0	R.IINEPTAASLAYGFEK.K
305 - 314	1192.6180	1191.6107	1191.6288	-15	0	R.IVDWLASNFK.R
340 - 363	2535.2440	2534.2367	2534.2938	-23	0	K.MELSSLTQTNISLPFITATADGPK.H
340 - 363	2551.2590	2550.2517	2550.2887	-15	0	K.MELSSLTQTNISLPFITATADGPK.H Oxidation (M)
372 - 384	1623.7910	1622.7837	1622.7974	-8	1	R.VKFEELCSDLLDR.L
374 - 384	1396.6370	1395.6297	1395.6340	-3	0	K.FEELCSDLLDR.L
402 - 414	1373.7180	1372.7107	1372.7198	-7	0	K.DLDEVILVGGSTR.I
492 - 514	2437.1650	2436.1577	2436.1769	-8	0	K.SEVFSTAADGQTSVEINVLQGER.E
528 - 536	935.5230	934.5157	934.5236	-8	0	R.LDGIPPAPR.G
565 - 583	2086.0580	2085.0507	2085.0702	-9	1	K.KQDITITGASTLPNDEVQR.M
566 - 583	1957.9510	1956.9437	1956.9753	-16	0	K.QDITITGASTLPNDEVQR.M
584 - 590	878.4230	877.4157	877.3963	22	0	R.MVQEAER.F Oxidation (M)
600 - 617	2024.9530	2023.9457	2023.9698	-12	1	R.DAIDTKNQADSVVYQTEK.Q
606 - 617	1381.6520	1380.6447	1380.6521	-5	0	K.NQADSVVYQTEK.Q

58.
Match to: **clementine0.9_003177m|PAC:19266047** Score: **77** Expect: **0.0026**
Nominal mass (M_r): **82705**; Calculated pI value: **4.68**
NCBI BLAST search of clementine0.9_003177m|PAC:19266047 against nr
Unformatted sequence string for pasting into other applications

Fixed modifications: Carbamidomethyl (C)
Variable modifications: Oxidation (M)
Cleavage by Trypsin: cuts C-term side of KR unless next residue is P
Number of mass values matched: **11**
Sequence Coverage: **23%**

Matched peptides shown in **Bold Red**

1 MWGRTCSSPK GLSMLQQTPL CWPFGATRIF LIFDERDKSA TAAK**KSEKPT**

```
 51 PIEIGGEVSQ MEAGSSIPKV QDQPTSSDDG MASVPSAVGE TVEDHEAPSK
101 EKGELADIAN RNDDPQNVMS GSSETLDGAL QTIEKETEET TLNQTIEETP
151 STDVSGELAE QALSTDGPKA GEFTESQTED TIAKDEVQIL TPATEEKETK
201 DTTEALAPEG SVSTEKQIIG EAASTNLSGE IAEQVSVSDS PKDEEVVQNQ
251 TDDVIAKDEE QIQTPTTESE IPSAGSLKEK ESGPIPDKNG SITSSGEEPD
301 VSSSQKTKAT VSPALVKQLR EETGAGMMDC KKALAETGGD IVKAQEFLRK
351 KGLASAEKKA SRATAEGRIG SYIHDSRIGV MVEVNCETDF VSRGDIFKEL
401 VDDLAMQVAA CPQVKYLVTE DVPEEIVNKE KEIEMQKEDL LSKPEQIRSK
451 IVEGRIRKRL EELALLEQPY IKNDKMVVKD WVKQTIATIG ENIKVKRFVR
501 YNLGEGLEKK SQDFAAEVAA QTAAKPIAKE QPAPAETKET VEKPPAVAVS
551 AALVKQLREE TGAGMMDCKK ALSETGGDLE KAQEYLRKKG LSSADKKSGR
601 LAAEGRIGSY IHDSRIGVLI EVNCETDFVG RSEKFKELVD DLAMQAVACP
651 QVQFVSIEDI PEDIINKEKE IEMQREDLIS KPENIRERIV EGRITKRLGE
701 LALSEQPFIK DDSVLVKDLV KQTVAAIGEN IKVRRFVRFT LGETNEETQT
751 ETEA
```

Start - End	Observed	Mr(expt)	Mr(calc)	ppm	Miss	Sequence
45 - 69	2615.2900	2614.2827	2614.3160	-13	1	K.KSEKPTPIEIGGEVSQMEAGSSIPK.V Oxidation (M)
136 - 169	3620.6840	3619.6767	3619.6748	1	0	K.ETEETTLNQTIEETPSTDVSGELAEQALSTDGPK.A
378 - 393	1854.8590	1853.8517	1853.8652	-7	0	R.IGVMVEVNCETDFVSR.G
378 - 393	1870.8590	1869.8517	1869.8601	-4	0	R.IGVMVEVNCETDFVSR.G Oxidation (M)
416 - 429	1647.8100	1646.8027	1646.8403	-23	0	K.YLVTEDVPEEIVNK.E
438 - 448	1327.6960	1326.6887	1326.7143	-19	0	K.EDLLSKPEQIR.S
459 - 472	1714.9460	1713.9387	1713.9665	-16	1	K.RLEELALLEQPYIK.N
616 - 631	1820.8920	1819.8847	1819.9138	-16	0	R.IGVLIEVNCETDFVGR.S
637 - 667	3515.6540	3514.6467	3514.7211	-21	0	K.ELVDDLAMQAVACPQVQFVSIEDIPEDIINK.E Oxidation (M)
637 - 669	3772.8190	3771.8117	3771.8587	-12	1	K.ELVDDLAMQAVACPQVQFVSIEDIPEDIINKEK.E Oxidation (M)
676 - 686	1313.7040	1312.6967	1312.6986	-1	0	R.EDLISKPENIR.E

67.
Match to: **orange1.1g024163m|PAC:18124685** Score: **133** Expect: **6.7e-009**
Nominal mass (M_r): **29262**; Calculated pI value: **5.32**
NCBI BLAST search of orange1.1g024163m|PAC:18124685 against nr
Unformatted sequence string for pasting into other applications

Fixed modifications: Carbamidomethyl (C)
Variable modifications: Oxidation (M)
Cleavage by Trypsin: cuts C-term side of KR unless next residue is P
Number of mass values matched: **14**
Sequence Coverage: **67%**

Matched peptides shown in **Bold Red**

```
  1 MLPRSLALPS PLLLSSFRSG ASAEGVPKRL TYDEIQSKTY MEVKGTGTAN
 51 QCPTIDGGVD SFAFKPGKYQ AKKFCLEPTS FTVKAESVNK NAPPDFQNTK
```

101 LMTR**LTYTLD EIEGPFEVSP DGTIK**FVEK**D GIDYAAVTVQ LPGGERVPFL**
151 **FTIKQLVASG KPENFGGEFL VPSYR**GSSFL DPKGR**GGSTG YDNAIALPAG**
201 **GRGDEEDLAK ENIK**NTSSST GKITLSVTK**S KPETGEVIGV FESLQPSDTD**
251 **MGAK**VPKDVK IQGIWYAQLE Q

Start - End	Observed	Mr(expt)	Mr(calc)	ppm	Miss	Sequence	
29 - 38	1252.6420	1251.6347	1251.6459	-9	1	K.RLTYDEIQSK.T	
45 - 68	2425.1350	2424.1277	2424.1380	-4	0	K.GTGTANQCPTIDGGVDSFAFKPGK.Y	
73 - 84	1456.7330	1455.7257	1455.7432	-12	1	K.KFCLEPTSFTVK.A	
74 - 84	1328.6410	1327.6337	1327.6482	-11	0	K.FCLEPTSFTVK.A	
74 - 90	1956.9430	1955.9357	1955.9663	-16	1	K.FCLEPTSFTVKAESVNK.N	
91 - 100	1131.5330	1130.5257	1130.5356	-9	0	K.NAPPDFQNTK.L	
105 - 125	2324.1330	2323.1257	2323.1471	-9	0	R.LTYTLDEIEGPFEVSPDGTIK.F	
130 - 146	1760.8660	1759.8587	1759.8741	-9	0	K.DGIDYAAVTVQLPGGER.V	
147 - 154	964.5550	963.5477	963.5793	-33	0	R.VPFLFTIK.Q	
155 - 175	2295.1600	2294.1527	2294.1695	-7	0	K.QLVASGKPENFGGEFLVPSYR.G	
186 - 202	1576.7580	1575.7507	1575.7641	-9	0	R.GGSTGYDNAIALPAGGR.G	
186 - 210	2434.1310	2433.1237	2433.1408	-7	1	R.GGSTGYDNAIALPAGGRGDEEDLAK.E	
203 - 214	1360.6830	1359.6757	1359.6517	18	1	R.GDEEDLAKENIK.N	
230 - 254	2638.2270	2637.2197	2637.2480	-11	0	K.SKPETGEVIGVFESLQPSDTDMGAK.V	Oxidation (M)

70.
Match to: **clementine0.9_012415m|PAC:19283832** Score: **67** Expect: **0.029**
Nominal mass (M_r): **42768**; Calculated pI value: **5.82**
NCBI BLAST search of clementine0.9_012415m|PAC:19283832 against nr
Unformatted sequence string for pasting into other applications

Fixed modifications: Carbamidomethyl (C)
Variable modifications: Oxidation (M)
Cleavage by Trypsin: cuts C-term side of KR unless next residue is P
Number of mass values matched: **15**
Sequence Coverage: **31%**

Matched peptides shown in **Bold Red**

1 METGITCYSR GVVTPTVSSQ RYTALVSPTS VSSSFGSRSL KASSLFGEPL
51 RVVPRSAVKV SKTKNSSLVT KCELGDSLEE FLTKATPDKA LIR**LMMCMGE**
101 **ALRTIAFK**VR TASCVGTACV NSFGDEQLAV DMLADK**LLFE ALTYSHFCK**Y
151 ACSEEVPELQ DMGGPAEGGF SVAFDPLDGS SIVDTNFTVG TIFGVWPGDK
201 LTGVTGR**DQV AAAMGIYGPR** TTYVIAIK**DF PGTHEFLLLD EGK**WQHVKET
251 TEIGEGK**MFS PGNLR**ATFDN PDYDKLINYY VKQKYTLR**YT GGMVPDVNQI**
301 **IVK**EK**GIFTN VTSPSSK**AKL R**LLFEVAPLG LLIENAGGYS SDGK**ISVLDK
351 VINDLDDRTQ VAYGSKNEII R**FEETLYGSS R**LKGGVPVGA AA

Start - End	Observed	Mr(expt)	Mr(calc)	ppm	Miss	Sequence	
94 - 103	1227.5400	1226.5327	1226.5280	4	0	R.LMMCMGEALR.T	Oxidation (M)

94 - 103	1243.5490	1242.5417	1242.5229	15	0	R.LMMCMGEALR.T	2 Oxidation (M)
94 - 103	1259.5680	1258.5607	1258.5178	34	0	R.LMMCMGEALR.T	3 Oxidation (M)
94 - 108	1819.8720	1818.8647	1818.8500	8	1	R.LMMCMGEALRTIAFK.V	3 Oxidation (M)
137 - 149	1628.7990	1627.7917	1627.8068	-9	0	K.LLFEALTYSHFCK.Y	
208 - 220	1348.6590	1347.6517	1347.6605	-7	0	R.DQVAAAMGIYGPR.T	
208 - 220	1364.6490	1363.6417	1363.6554	-10	0	R.DQVAAAMGIYGPR.T	Oxidation (M)
229 - 243	1717.8300	1716.8227	1716.8359	-8	0	K.DFPGTHEFLLLDEGK.W	
258 - 265	921.4430	920.4357	920.4538	-20	0	K.MFSPGNLR.A	
258 - 265	937.4540	936.4467	936.4487	-2	0	K.MFSPGNLR.A	Oxidation (M)
289 - 303	1633.8230	1632.8157	1632.8545	-24	0	R.YTGGMVPDVNQIIVK.E	
289 - 303	1649.8190	1648.8117	1648.8495	-23	0	R.YTGGMVPDVNQIIVK.E	Oxidation (M)
306 - 317	1237.6180	1236.6107	1236.6350	-20	0	K.GIFTNVTSPSSK.A	
322 - 344	2363.2060	2362.1987	2362.2420	-18	0	R.LLFEVAPLGLLIENAGGYSSDGK.I	
372 - 381	1188.5460	1187.5387	1187.5459	-6	0	R.FEETLYGSSR.L	

71.
Match to: **clementine0.9_009588m|PAC:19284301** Score: **145** Expect: **4.3e-010**
Nominal mass (M_r): **51073**; Calculated pI value: **5.33**
NCBI BLAST search of clementine0.9_009588m|PAC:19284301 against nr
Unformatted sequence string for pasting into other applications

Fixed modifications: Carbamidomethyl (C)
Variable modifications: Oxidation (M)
Cleavage by Trypsin: cuts C-term side of KR unless next residue is P
Number of mass values matched: **20**
Sequence Coverage: **50%**

Matched peptides shown in **Bold Red**

1 MAAALSFNGS GAATSVPSSS FFGTSLKKVS SRIPPSKVPS ASFKITAEVD
51 ENKQTKKDRW K**GLAYDESDD QQDITR**GK**GA VDSLFQAPMG TGTHYAVMSS**
101 **YDYISQGLRT YNLDNTMDGL YIAPAFMDK**L VVHITKNFMS LPNIKVPLIL
151 GIWGGKGQGK **SFQCELVFAK MGINPIMMSA GELESGNAGE PAK**LIRQRYR
201 EAADIIKKGK **MCCLMINDLD AGAGR**MGGTT QYTVNNQMVN ATLMNIADNP
251 TCVQLPGMYN KEENPR**VPII VTGNDFSTLY APLIR**DGRME K**FYWAPTR**ED
301 RIGVCKGIFR NDNVADDDIV K**LVDTFPGQS IDFFGALR**AR VYDDEVRKWI
351 SGVGVGSIGK SLVNSK**EAAP TFEQPR**MTME K**LLEYGNMIV QEQENVKR**VQ
401 LADK**YLSEAA LGEANEDAIQ SGNFYGKAAQ QMNVPVPEGC TDPTAENFDP**
451 **TAR**SDDGSCQ YTL

Start - End	Observed	Mr(expt)	Mr(calc)	ppm	Miss	Sequence	
62 - 76	1725.7430	1724.7357	1724.7489	-8	0	K.GLAYDESDDQQDITR.G	
79 - 109	3322.5550	3321.5477	3321.5435	1	0	K.GAVDSLFQAPMGTGTHYAVMSSYDYISQGLR.T	
79 - 109	3338.6480	3337.6407	3337.5384	31	0	K.GAVDSLFQAPMGTGTHYAVMSSYDYISQGLR.T	Oxidation (M)
79 - 109	3354.5440	3353.5367	3353.5333	1	0	K.GAVDSLFQAPMGTGTHYAVMSSYDYISQGLR.T	2 Oxidation (M)

110 - 129	2309.0380	2308.0307	2308.0392	-4	0	R.TYNLDNTMDGLYIAPAFMDK.L	Oxidation (M)
161 - 170	1228.5980	1227.5907	1227.5958	-4	0	K.SFQCELVFAK.M	
171 - 193	2336.0410	2335.0337	2335.0494	-7	0	K.MGINPIMMSAGELESGNAGEPAK.L	2 Oxidation (M)
171 - 193	2352.0440	2351.0367	2351.0443	-3	0	K.MGINPIMMSAGELESGNAGEPAK.L	3 Oxidation (M)
211 - 225	1696.7250	1695.7177	1695.7201	-1	0	K.MCCLMINDLDAGAGR.M	
211 - 225	1712.7200	1711.7127	1711.7150	-1	0	K.MCCLMINDLDAGAGR.M	Oxidation (M)
267 - 285	2089.1560	2088.1487	2088.1619	-6	0	R.VPIIVTGNDFSTLYAPLIR.D	
292 - 298	940.4610	939.4537	939.4603	-7	0	K.FYWAPTR.E	
322 - 338	1882.9610	1881.9537	1881.9625	-5	0	K.LVDTFPGQSIDFFGALR.A	
367 - 376	1145.5500	1144.5427	1144.5513	-7	0	K.EAAPTFEQPR.M	
382 - 397	1906.9200	1905.9127	1905.9506	-20	0	K.LLEYGNMIVQEQENVK.R	
382 - 398	2063.0320	2062.0247	2062.0517	-13	1	K.LLEYGNMIVQEQENVKR.V	
382 - 398	2079.0360	2078.0287	2078.0466	-9	1	K.LLEYGNMIVQEQENVKR.V	Oxidation (M)
405 - 427	2447.1250	2446.1177	2446.1288	-5	0	K.YLSEAALGEANEDAIQSGNFYGK.A	
428 - 453	2816.2400	2815.2327	2815.2542	-8	0	K.AAQQMNVPVPEGCTDPTAENFDPTAR.S	
428 - 453	2832.2330	2831.2257	2831.2491	-8	0	K.AAQQMNVPVPEGCTDPTAENFDPTAR.S	Oxidation (M)

72.
Match to: **orange1.1g014244m|PAC:18138111** Score: **144** Expect: **5.4e-010**
Nominal mass (M_r): **47200**; Calculated pI value: **5.94**
NCBI BLAST search of orange1.1g014244m|PAC:18138111 against nr
Unformatted sequence string for pasting into other applications

Fixed modifications: Carbamidomethyl (C)
Variable modifications: Oxidation (M)
Cleavage by Trypsin: cuts C-term side of KR unless next residue is P
Number of mass values matched: **21**
Sequence Coverage: **55%**

Matched peptides shown in **Bold Red**

```
  1 MAAAVPLSFN GSGAATSVPS SSFFGTSLKK VSSRIPPSKV PSASFKITAE
 51 VDENKQTKKD RWKGLAYDES DDQQDITRGK GAVDSLFQAP MGTGTHYAVM
101 SSYDYISQGL RTYNLDNTID GLYIAPAFMD KLVVHITKNF MSLPNIKVPL
151 ILGIWGGKGQ GKSFQCELVF AKMGINPIMM SAGELESGNA GEPAKLIRQR
201 YREAADIIKK GKMCCLMIND LDAGAGRMGG TTQYTVNNQM VNATLMNIAD
251 NPTCVQLPGM YNKEENPRVP IIVTGNDFST LYAPLIRDGR MEKFYWAPTR
301 EDRIGVCKGI FRNDNVADDD IVKLVDTFPG QSIDFFGALR ARVYDDEVRK
351 WISGVGVGSI GKSLVNSKEA APTFEQPRMT MEKLLEYGNM IVQEQENVKR
401 VQLADKYLSE AALGEANEDA IQSGNFYG
```

Start - End	Observed	Mr(expt)	Mr(calc)	ppm	Miss	Sequence	
64 - 78	1725.7490	1724.7417	1724.7489	-4	0	K.GLAYDESDDQQDITR.G	
81 - 111	3322.5330	3321.5257	3321.5435	-5	0	K.GAVDSLFQAPMGTGTHYAVMSSYDYISQGLR.T	
81 - 111	3338.5780	3337.5707	3337.5384	10	0	K.GAVDSLFQAPMGTGTHYAVMSSYDYISQGLR.T	Oxidation (M)
81 - 111	3354.5250	3353.5177	3353.5333	-5	0	K.GAVDSLFQAPMGTGTHYAVMSSYDYISQGLR.T	2 Oxidation (M)

112 - 131	2275.0780	2274.0707	2274.0878	-8	0	**R.TYNLDNTIDGLYIAPAFMDK.L**
112 - 131	2291.0680	2290.0607	2290.0827	-10	0	**R.TYNLDNTIDGLYIAPAFMDK.L** Oxidation (M)
148 - 158	1152.7050	1151.6977	1151.7067	-8	0	**K.VPLILGIWGGK.G**
163 - 172	1228.5970	1227.5897	1227.5958	-5	0	**K.SFQCELVFAK.M**
173 - 195	2320.0520	2319.0447	2319.0545	-4	0	**K.MGINPIMMSAGELESGNAGEPAK.L** Oxidation (M)
173 - 195	2336.0360	2335.0287	2335.0494	-9	0	**K.MGINPIMMSAGELESGNAGEPAK.L** 2 Oxidation (M)
173 - 195	2352.0460	2351.0387	2351.0443	-2	0	**K.MGINPIMMSAGELESGNAGEPAK.L** 3 Oxidation (M)
213 - 227	1696.7210	1695.7137	1695.7201	-4	0	**K.MCCLMINDLDAGAGR.M**
213 - 227	1712.7110	1711.7037	1711.7150	-7	0	**K.MCCLMINDLDAGAGR.M** Oxidation (M)
228 - 263	3989.7790	3988.7717	3988.8250	-13	0	**R.MGGTTQYTVNNQMVNATLMNIADNPTCVQLPGMYNK.E**
269 - 287	2089.1620	2088.1547	2088.1619	-3	0	**R.VPIIVTGNDFSTLYAPLIR.D**
294 - 300	940.4630	939.4557	939.4603	-5	0	**K.FYWAPTR.E**
324 - 340	1882.9630	1881.9557	1881.9625	-4	0	**K.LVDTFPGQSIDFFGALR.A**
343 - 349	895.4130	894.4057	894.4083	-3	0	**R.VYDDEVR.K**
369 - 378	1145.5540	1144.5467	1144.5513	-4	0	**K.EAAPTFEQPR.M**
384 - 400	2063.0410	2062.0337	2062.0517	-9	1	**K.LLEYGNMIVQEQENVKR.V**
384 - 400	2079.0360	2078.0287	2078.0466	-9	1	**K.LLEYGNMIVQEQENVKR.V** Oxidation (M)

75.
Match to: **clementine0.9_010096m|PAC:19269912** Score: **197** Expect: **2.7e-015**
Nominal mass (M_r): **50941**; Calculated pI value: **4.76**
NCBI BLAST search of clementine0.9_010096m|PAC:19269912 against nr
Unformatted sequence string for pasting into other applications

Fixed modifications: Carbamidomethyl (C)
Variable modifications: Oxidation (M)
Cleavage by Trypsin: cuts C-term side of KR unless next residue is P
Number of mass values matched: **27**
Sequence Coverage: **60%**

Matched peptides shown in **Bold Red**

```
  1 MREILHIQGG QCGNQIGSKF WEVICDEHGV DPTGKYRGDG VEDLQLERIN
 51 VYYNEASGGR YVPRAVLMDL EPGTMDSIRS GPYGQIFRPD NFVFGQSGAG
101 NNWAKGHYTE GAELIDAVLD VVRKEAENCD CLQGFQVCHS LGGGTGSGMG
151 TLLISKIREE YPDRMMMTFS VFPSPKVSDT VVEPYNATLS VHQLVENADE
201 CMVLDNEALY DICFRTLKLS TPSFGDLNHL ISATMSGVTC CLRFPGQLNS
251 DLRKLAVNLI PFPRLHFFMV GFAPLTSRGS QQYISLTVPE LTQQMWDAKN
301 MMCAADPRHG RYLTASAMFR GKMSTKEVDE QMINVQNKNS SYFVEWIPNN
351 VKSSVCDIPP RGLKMASTFI GNSTSIQEMF RRVSEQFTAM FRRKAFLHWY
401 TGEGMDEMEF TEAESNMNDL VAEYQQYQDA TADDEEEYEE GLEESFEG
```

Start - End	Observed	Mr(expt)	Mr(calc)	ppm	Miss	Sequence
3 - 19	1838.9000	1837.8927	1837.9105	-10	0	**R.EILHIQGGQCGNQIGSK.F**
20 - 35	1888.8510	1887.8437	1887.8462	-1	0	**K.FWEVICDEHGVDPTGK.Y**

36 - 48	1549.7460	1548.7387	1548.7532	-9	1	K.YRGDGVEDLQLER.I	
49 - 60	1342.6290	1341.6217	1341.6313	-7	0	R.INVYYNEASGGR.Y	
65 - 79	1663.7860	1662.7787	1662.7957	-10	0	R.AVLMDLEPGTMDSIR.S	Oxidation (M)
65 - 79	1679.7910	1678.7837	1678.7906	-4	0	R.AVLMDLEPGTMDSIR.S	2 Oxidation (M)
80 - 105	2814.3120	2813.3047	2813.3310	-9	0	R.SGPYGQIFRPDNFVFGQSGAGNNWAK.G	
106 - 123	1956.9860	1955.9787	1955.9953	-8	0	K.GHYTEGAELIDAVLDVVR.K	
157 - 164	1077.5280	1076.5207	1076.5250	-4	1	K.IREEYPDR.M	
216 - 243	3095.5720	3094.5647	3094.5250	13	1	R.TLKLSTPSFGDLNHLISATMSGVTCCLR.F	Oxidation (M)
219 - 243	2737.2920	2736.2847	2736.3034	-7	0	K.LSTPSFGDLNHLISATMSGVTCCLR.F	
244 - 253	1146.5830	1145.5757	1145.5829	-6	0	R.FPGQLNSDLR.K	
244 - 254	1274.6510	1273.6437	1273.6779	-27	1	R.FPGQLNSDLRK.L	
254 - 264	1267.7770	1266.7697	1266.7812	-9	1	R.KLAVNLIPFPR.L	
255 - 264	1139.6870	1138.6797	1138.6862	-6	0	K.LAVNLIPFPR.L	
265 - 278	1622.8280	1621.8207	1621.8439	-14	0	R.LHFFMVGFAPLTSR.G	
265 - 278	1638.8290	1637.8217	1637.8388	-10	0	R.LHFFMVGFAPLTSR.G	Oxidation (M)
279 - 299	2423.1790	2422.1717	2422.1839	-5	0	R.GSQQYISLTVPELTQQMWDAK.N	
279 - 299	2439.1860	2438.1787	2438.1788	-0	0	R.GSQQYISLTVPELTQQMWDAK.N	Oxidation (M)
300 - 311	1431.6320	1430.6247	1430.5965	20	1	K.NMMCAADPRHGR.Y	Oxidation (M)
312 - 320	1059.5170	1058.5097	1058.5219	-11	0	R.YLTASAMFR.G	
312 - 320	1075.5200	1074.5127	1074.5168	-4	0	R.YLTASAMFR.G	Oxidation (M)
339 - 352	1696.8080	1695.8007	1695.8257	-15	0	K.NSSYFVEWIPNNVK.S	
365 - 381	1919.8740	1918.8667	1918.8917	-13	0	K.MASTFIGNSTSIQEMFR.R	
365 - 381	1935.8770	1934.8697	1934.8866	-9	0	K.MASTFIGNSTSIQEMFR.R	Oxidation (M)
383 - 392	1215.5730	1214.5657	1214.5754	-8	0	R.VSEQFTAMFR.R	
383 - 392	1231.5740	1230.5667	1230.5703	-3	0	R.VSEQFTAMFR.R	Oxidation (M)

78.
Match to: **orange1.1g037616m|PAC:18125034** Score: **102** Expect: **8.5e-006**
Nominal mass (M_r): **42534**; Calculated pI value: **5.82**
NCBI BLAST search of orange1.1g037616m|PAC:18125034 against nr
Unformatted sequence string for pasting into other applications

Fixed modifications: Carbamidomethyl (C)
Variable modifications: Oxidation (M)
Cleavage by Trypsin: cuts C-term side of KR unless next residue is P
Number of mass values matched: **15**
Sequence Coverage: **59%**

Matched peptides shown in **Bold Red**

```
  1 MRECISVHIG QAGIQVGNAC WELYCLEHGI QPDGQMPSDK TVGGGDDAFN
 51 TFFSETGAGK HVPRAVFVDL EPTVIDEVRT GTYRQLFHPE QLISGKEDAA
101 NNFARGHYTI GKEIVDLCLD RIRKLADNCT GLQGFLVFNA VGGGTGSGLG
151 SLLLERLSVD YGKKSKLGFT VYPSPQVSTS VVEPYNSVLS THSLLEHTDV
201 AVLLDNEAIY DICRRSLDIE RPTYTNLNRL VSQVISSLTA SLRFDGALNV
```

251 **DVTEFQTNLV PYPRIHFMLS SYAPVISAEK AYHEQLSVAE ITNSAFEPSS**
301 **MMAK**CDPRHG K**YMACCLMYR** GDVVPKDVNA AVATIKTKR**T IQFVDWCPTG**
351 **FKCGINYQPP TVVPGGDLAK** VQRAVCMISN STS

Start - End	Observed	Mr(expt)	Mr(calc)	ppm	Miss	Sequence
41 - 60	1977.8730	1976.8657	1976.8752	-5	0	K.TVGGGDDAFNTFFSETGAGK.H
65 - 79	1701.8930	1700.8857	1700.8985	-8	0	R.AVFVDLEPTVIDEVR.T
85 - 96	1396.7360	1395.7287	1395.7510	-16	0	R.QLFHPEQLISGK.E
97 - 105	1007.4500	1006.4427	1006.4468	-4	0	K.EDAANNFAR.G
113 - 121	1132.5560	1131.5487	1131.5594	-9	0	K.EIVDLCLDR.I
125 - 156	3193.5770	3192.5697	3192.6238	-17	0	K.LADNCTGLQGFLVFNAVGGGTGSGLGSLLLER.L
216 - 229	1691.8540	1690.8467	1690.8638	-10	0	R.SLDIERPTYTNLNR.L
230 - 243	1473.8490	1472.8417	1472.8562	-10	0	R.LVSQVISSLTASLR.F
244 - 264	2395.1720	2394.1647	2394.1856	-9	0	R.FDGALNVDVTEFQTNLVPYPR.I
265 - 280	1792.8920	1791.8847	1791.9229	-21	0	R.IHFMLSSYAPVISAEK.A
281 - 304	2657.2060	2656.1987	2656.2149	-6	0	K.AYHEQLSVAEITNSAFEPSSMMAK.C Oxidation (M)
281 - 304	2673.1930	2672.1857	2672.2098	-9	0	K.AYHEQLSVAEITNSAFEPSSMMAK.C 2 Oxidation (M)
312 - 320	1283.5310	1282.5237	1282.4967	21	0	K.YMACCLMYR.G Oxidation (M)
340 - 352	1598.7530	1597.7457	1597.7599	-9	0	R.TIQFVDWCPTGFK.C
353 - 370	1885.9040	1884.8967	1884.9404	-23	0	K.CGINYQPPTVVPGGDLAK.V

81.
Match to: **clementine0.9_007330m|PAC:19281955** Score: **210** Expect: **1.3e-016**
Nominal mass (M_r): **55491**; Calculated pI value: **4.86**
NCBI BLAST search of clementine0.9_007330m|PAC:19281955 against nr
Unformatted sequence string for pasting into other applications

Fixed modifications: Carbamidomethyl (C)
Variable modifications: Oxidation (M)
Cleavage by Trypsin: cuts C-term side of KR unless next residue is P
Number of mass values matched: **22**
Sequence Coverage: **60%**

Matched peptides shown in **Bold Red**

1 MQAGIDK**LSD AVGLTLGPR**G R**NVVLDEFGS PK**VVNDGVTI AR**AIELADPM**
51 **ENAGAALIRE** VASK**TNDSAG DGTTTASVLA R**EIIK**LGLLS VTSGANPVSL**
101 **KR**GIDK**TVHG LVEELEKR**AR PIEGRDDIKA VATISAGNDD LIGTMIADAI
151 DK**VGPDGVLS IESSSSFETT VEVEEGMEID RGYISPQFVT NPEKLIVEFE**
201 **NAR**VLVTDQK ISAIKDIIPL LEKTTQLR**AP LLIIAEDVTG EALATLVVNK**
251 LRGILNVAAI KAPGFGERRK **ALLQDIAIVT GAEFQAGDLG LLIENTSVEQ**
301 **LGTAR**KVTIR K**DSTTIIADA ASKDEIQAR**I AQLKK**ELAET DSVYDSEK**LA
351 ERIAKLSGGV AVIK**VGAATE TELEDR**KLRI EDAK**NATFAA IEEGIVPGGG**
401 **AALVHLSDHV PAIK**DKLEDA DERLGADIVQ K**ALVAPASLI AHNAGVEGEV**
451 **VVEK**VKDSEW TTGYNAMTDK YENMLQAGVI DPAKVTR**CAL QNAASVAGMV**

501 **LTTQAIVVEK PKPK**TPVAAP PQGLMV

Start - End	Observed	Mr(expt)	Mr(calc)	ppm	Miss	Sequence	
**8 - 19**	**1198.6730**	**1197.6657**	**1197.6717**	**-5**	**0**	**K.LSDAVGLTLGPR.G**	
**22 - 32**	**1204.6070**	**1203.5997**	**1203.6136**	**-11**	**0**	**R.NVVLDEFGSPK.V**	
**43 - 59**	**1754.8940**	**1753.8867**	**1753.9032**	**-9**	**0**	**R.AIELADPMENAGAALIR.E**	
**43 - 59**	**1770.8930**	**1769.8857**	**1769.8981**	**-7**	**0**	**R.AIELADPMENAGAALIR.E**	Oxidation (M)
**65 - 81**	**1636.7710**	**1635.7637**	**1635.7700**	**-4**	**0**	**K.TNDSAGDGTTTASVLAR.E**	
**86 - 101**	**1555.8910**	**1554.8837**	**1554.8981**	**-9**	**0**	**K.LGLLSVTSGANPVSLK.R**	
**86 - 102**	**1711.9670**	**1710.9597**	**1710.9992**	**-23**	**1**	**K.LGLLSVTSGANPVSLKR.G**	
**107 - 117**	**1253.6580**	**1252.6507**	**1252.6663**	**-12**	**0**	**K.TVHGLVEELEK.R**	
**107 - 118**	**1409.7610**	**1408.7537**	**1408.7674**	**-10**	**1**	**K.TVHGLVEELEKR.A**	
**153 - 181**	**3098.4310**	**3097.4237**	**3097.4285**	**-2**	**0**	**K.VGPDGVLSIESSSSFETTVEVEEGMEIDR.G**	
**153 - 181**	**3114.4070**	**3113.3997**	**3113.4234**	**-8**	**0**	**K.VGPDGVLSIESSSSFETTVEVEEGMEIDR.G**	Oxidation (M)
**182 - 194**	**1479.7370**	**1478.7297**	**1478.7405**	**-7**	**0**	**R.GYISPQFVTNPEK.L**	
**195 - 203**	**1090.5820**	**1089.5747**	**1089.5818**	**-7**	**0**	**K.LIVEFENAR.V**	
**229 - 250**	**2250.2460**	**2249.2387**	**2249.2882**	**-22**	**0**	**R.APLLIIAEDVTGEALATLVVNK.L**	
**271 - 305**	**3626.8860**	**3625.8787**	**3625.9203**	**-11**	**0**	**K.ALLQDIAIVTGAEFQAGDLGLLIENTSVEQLGTAR.K**	
**312 - 329**	**1904.9360**	**1903.9287**	**1903.9487**	**-10**	**1**	**K.DSTTIIADAASKDEIQAR.I**	
**336 - 348**	**1485.6840**	**1484.6767**	**1484.6518**	**17**	**0**	**K.ELAETDSVYDSEK.L**	
**365 - 376**	**1290.6090**	**1289.6017**	**1289.6099**	**-6**	**0**	**K.VGAATETELEDR.K**	
**385 - 414**	**2954.5520**	**2953.5447**	**2953.5661**	**-7**	**0**	**K.NATFAAIEEGIVPGGGAALVHLSDHVPAIK.D**	
**432 - 454**	**2273.2270**	**2272.2197**	**2272.2427**	**-10**	**0**	**K.ALVAPASLIAHNAGVEGEVVVEK.V**	
**488 - 514**	**2824.4730**	**2823.4657**	**2823.5350**	**-25**	**0**	**R.CALQNAASVAGMVLTTQAIVVEKPKPK.T**	
**488 - 514**	**2840.4910**	**2839.4837**	**2839.5300**	**-16**	**0**	**R.CALQNAASVAGMVLTTQAIVVEKPKPK.T**	Oxidation (M)

86.
Match to: **clementine0.9_004610m|PAC:19284277** Score: **118** Expect: **2.1e-007**
Nominal mass (M_r): **71346**; Calculated pI value: **5.10**
NCBI BLAST search of clementine0.9_004610m|PAC:19284277 against nr
Unformatted sequence string for pasting into other applications

Fixed modifications: Carbamidomethyl (C)
Variable modifications: Oxidation (M)
Cleavage by Trypsin: cuts C-term side of KR unless next residue is P
Number of mass values matched: **22**
Sequence Coverage: **47%**

Matched peptides shown in **Bold Red**

1 MAGK**GEGPAI GIDLGTTYSC VGVWQHDRVE IIANDQGNRT TPSYVGFTDT**
51 **ER**LIGDAAKN **QVAMNPTNTV FDAK**RLIGRR FSDASVQGDM KLWPFK**VIAG**
101 **PADKPMIGVN YKGEEKQFAA EEISSMVLIK** MREIAEAYLG STIK**NAVVTV**
151 **PAYFNDSQRQ ATKDAGVIAG LNVMR**IINEP TAAAIAYGLD KKATSVGEKN
201 VLIFDLGGGT FDVSLLTIEE GIFEVK**ATAG DTHLGGEDFD NR**MVNHFVQE

```
251 FKRKNKKDIS GNPRALRRLR TACERAKRTL SSTAQTTIEI DSLYEGIDFY
301 STITRARFEE LNMDLFRKCM EPVEKCLRDA KMDKSTVHDV VLVGGSTRIP
351 KVQQLLQDFF NGKELCKNIN PDEAVAYGAA VQAAILSGEG NEKVQDLLLL
401 DVTPLSLGLE TAGGVMTVLI PRNTTIPTKK EQVFSTYSDN QPGVLIQVYE
451 GERTRTRDNN LLGKFELSGI PPAPRGVPQI TVCFDIDANG ILNVSAEDKT
501 TGQKNKITIT NDKGRLSKDE IEKMVQEAEK YKAEDEEHKK KVEAKNALEN
551 YAYNMRNTVK DEKIGSKLDP ADKKKIEDAI DQAIQWLDSN QLAEADEFED
601 KMKELESICN PIIAKMYQGA GGDMGGGMDD DAPPAGGSAA GPKIEEVD
```

Start - End	Observed	Mr(expt)	Mr(calc)	ppm	Miss	Sequence
5 - 28	2588.2020	2587.1947	2587.2126	-7	0	K.GEGPAIGIDLGTTYSCVGVWQHDR.V
29 - 39	1228.6130	1227.6057	1227.6207	-12	0	R.VEIIANDQGNR.T
40 - 52	1473.6840	1472.6767	1472.6784	-1	0	R.TTPSYVGFTDTER.L
60 - 74	1649.7730	1648.7657	1648.7879	-13	0	K.NQVAMNPTNTVFDAK.R
60 - 74	1665.8010	1664.7937	1664.7828	7	0	K.NQVAMNPTNTVFDAK.R Oxidation (M)
97 - 116	2116.0650	2115.0577	2115.1034	-22	1	K.VIAGPADKPMIGVNYKGEEK.Q
113 - 130	2024.9570	2023.9497	2024.0136	-32	1	K.GEEKQFAAEEISSMVLIK.M Oxidation (M)
117 - 130	1565.7900	1564.7827	1564.8170	-22	0	K.QFAAEEISSMVLIK.M
145 - 159	1680.8160	1679.8087	1679.8267	-11	0	K.NAVVTVPAYFNDSQR.Q
160 - 175	1659.8650	1658.8577	1658.8774	-12	1	R.QATKDAGVIAGLNVMR.I Oxidation (M)
164 - 175	1215.6150	1214.6077	1214.6441	-30	0	K.DAGVIAGLNVMR.I
164 - 175	1231.6140	1230.6067	1230.6391	-26	0	K.DAGVIAGLNVMR.I Oxidation (M)
227 - 242	1675.7260	1674.7187	1674.7234	-3	0	K.ATAGDTHLGGEDFDNR.M
279 - 305	3025.4500	3024.4427	3024.4815	-13	0	R.TLSSTAQTTIEIDSLYEGIDFYSTITR.A
308 - 317	1313.6190	1312.6117	1312.6122	-0	0	R.FEELNMDLFR.K
308 - 317	1329.6160	1328.6087	1328.6071	1	0	R.FEELNMDLFR.K Oxidation (M)
335 - 348	1426.7520	1425.7447	1425.7576	-9	0	K.STVHDVVLVGGSTR.I
368 - 393	2601.3350	2600.3277	2600.2718	22	0	K.NINPDEAVAYGAAVQAAILSGEGNEK.V
431 - 453	2658.2510	2657.2437	2657.2609	-6	0	K.EQVFSTYSDNQPGVLIQVYEGER.T
476 - 499	2575.2310	2574.2237	2574.2636	-15	0	R.GVPQITVCFDIDANGILNVSAEDK.T
546 - 556	1374.6270	1373.6197	1373.6033	12	0	K.NALENYAYNMR.N Oxidation (M)
575 - 601	3134.4760	3133.4687	3133.4727	-1	1	K.KIEDAIDQAIQWLDSNQLAEADEFEDK.M

91.
Match to: **orange1.1g024163m|PAC:18124685** Score: **134** Expect: **5.4e-009**
Nominal mass (M_r): **29262**; Calculated pI value: **5.32**
NCBI BLAST search of orange1.1g024163m|PAC:18124685 against nr
Unformatted sequence string for pasting into other applications

Fixed modifications: Carbamidomethyl (C)
Variable modifications: Oxidation (M)
Cleavage by Trypsin: cuts C-term side of KR unless next residue is P
Number of mass values matched: **15**
Sequence Coverage: **70%**

Matched peptides shown in **Bold Red**

1 MLPRSLALPS PLLLSSFRSG ASAEGVPK**RL TYDEIQSK**TY MEVK**GTGTAN**

51 **QCPTIDGGVD SFAFKPGK**YQ AK**KFCLEPTS FTVK**AESVNK **NAPPDFQNTK**

101 LMTR**LTYTLD EIEGPFEVSP DGTIK**FVEK**D GIDYAAVTVQ LPGGERVPFL**

151 **FTIKQLVASG KPENFGGEFL VPSYR**GSSFL DPKGR**GGSTG YDNAIALPAG**

201 **GRGDEEDLAK ENIK**NTSSST GKITLSVTK**S KPETGEVIGV FESLQPSDTD**

251 **MGAK**VPK**DVK IQGIWYAQLE Q**

Start - End	Observed	Mr(expt)	Mr(calc)	ppm	Miss	Sequence	
29 - 38	1252.6450	1251.6377	1251.6459	-7	1	K.RLTYDEIQSK.T	
45 - 68	2425.1310	2424.1237	2424.1380	-6	0	K.GTGTANQCPTIDGGVDSFAFKPGK.Y	
73 - 84	1456.7390	1455.7317	1455.7432	-8	1	K.KFCLEPTSFTVK.A	
74 - 84	1328.6290	1327.6217	1327.6482	-20	0	K.FCLEPTSFTVK.A	
91 - 100	1131.5360	1130.5287	1130.5356	-6	0	K.NAPPDFQNTK.L	
105 - 125	2324.1380	2323.1307	2323.1471	-7	0	R.LTYTLDEIEGPFEVSPDGTIK.F	
130 - 146	1760.8710	1759.8637	1759.8741	-6	0	K.DGIDYAAVTVQLPGGER.V	
147 - 154	964.5460	963.5387	963.5793	-42	0	R.VPFLFTIK.Q	
155 - 175	2295.1640	2294.1567	2294.1695	-6	0	K.QLVASGKPENFGGEFLVPSYR.G	
186 - 202	1576.7620	1575.7547	1575.7641	-6	0	R.GGSTGYDNAIALPAGGR.G	
186 - 210	2434.1220	2433.1147	2433.1408	-11	1	R.GGSTGYDNAIALPAGGRGDEEDLAK.E	
203 - 214	1360.6920	1359.6847	1359.6517	24	1	R.GDEEDLAKENIK.N	
230 - 254	2622.2400	2621.2327	2621.2531	-8	0	K.SKPETGEVIGVFESLQPSDTDMGAK.V	
230 - 254	2638.2290	2637.2217	2637.2480	-10	0	K.SKPETGEVIGVFESLQPSDTDMGAK.V	Oxidation (M)
258 - 271	1690.8460	1689.8387	1689.8726	-20	1	K.DVKIQGIWYAQLEQ.-	

95.
Match to: **clementine0.9_022968m|PAC:19271622** Score: **74** Expect: **0.0048**
Nominal mass (M_r): **20972**; Calculated pI value: **5.39**
NCBI BLAST search of clementine0.9_022968m|PAC:19271622 against nr
Unformatted sequence string for pasting into other applications

Fixed modifications: Carbamidomethyl (C)
Variable modifications: Oxidation (M)
Cleavage by Trypsin: cuts C-term side of KR unless next residue is P
Number of mass values matched: **8**
Sequence Coverage: **57%**

Matched peptides shown in **Bold Red**

1 M**TTSADEGQF LNMLLK**LVNA KNTMEIGVYT GYSLLATALA LPDDGKILAM

51 DINR**ENYELG LPVIQK**AGVA HKIDFR**EGPA LPVLDLLIQD EKNHGSFDFI**

101 **FVDADKDNYL NYHK**RLVELV K**VGGVIGYDN TLWNGSVVAP PDAPLRK**YVR

151 YYRDFVLELN KALAADPR**IE ICMLPVGDGV TICR**RIK

Start - End	Observed	Mr(expt)	Mr(calc)	ppm	Miss	Sequence
2 - 16	1667.7970	1666.7897	1666.8236	-20	0	M.TTSADEGQFLNMLLK.L
55 - 66	1402.7190	1401.7117	1401.7504	-28	0	R.ENYELGLPVIQK.A
77 - 92	1749.9390	1748.9317	1748.9560	-14	0	R.EGPALPVLDLLIQDEK.N
93 - 114	2659.2090	2658.2017	2658.2139	-5	1	K.NHGSFDFIFVDADKDNYLNYHK.R
122 - 146	2567.2860	2566.2787	2566.3180	-15	0	K.VGGVIGYDNTLWNGSVVAPPDAPLR.K
122 - 147	2695.3690	2694.3617	2694.4130	-19	1	K.VGGVIGYDNTLWNGSVVAPPDAPLRK.Y
169 - 184	1832.8960	1831.8887	1831.8995	-6	0	R.IEICMLPVGDGVTICR.R
169 - 184	1848.8860	1847.8787	1847.8944	-8	0	R.IEICMLPVGDGVTICR.R Oxidation (M)

97.
Match to: **clementine0.9_018314m|PAC:19269827** Score: **109** Expect: **1.7e-006**
Nominal mass (M_r): **29940**; Calculated pI value: **5.15**
NCBI BLAST search of clementine0.9_018314m|PAC:19269827 against nr
Unformatted sequence string for pasting into other applications

Fixed modifications: Carbamidomethyl (C)
Variable modifications: Oxidation (M)
Cleavage by Trypsin: cuts C-term side of KR unless next residue is P
Number of mass values matched: **10**
Sequence Coverage: **49%**

Matched peptides shown in **Bold Red**

1 MFR**NQYDTDV TTWSPAGRLF QVEYAMEAVK** QGSAAIGLRS KTHVVLGCVN
51 KANSELSSHQ KKIFK**VDDHI GVAIAGLTAD GR**VLSRYMR**S ECINYSYTYE**
101 **SPLPVGR**LVV QLADKAQRSW K**RPYGVGLLV AGLDEKGAHL YYNCPSGNYF**
151 **EYQAFAIGSR** SQAAKTYLER **RFENFSESTR** EDLIKDALMA IRETLQGETL
201 K**SSICTVAVV GAGEPFHILD QETVQK**LIDS FEIAGTEEGP AAAPDSAAEG
251 GSAAEGGAAS EQGAPADEGV APMDI

Start - End	Observed	Mr(expt)	Mr(calc)	ppm	Miss	Sequence
4 - 18	1710.7610	1709.7537	1709.7645	-6	0	R.NQYDTDVTTWSPAGR.L
19 - 30	1427.7240	1426.7167	1426.7166	0	0	R.LFQVEYAMEAVK.Q
19 - 30	1443.7000	1442.6927	1442.7115	-13	0	R.LFQVEYAMEAVK.Q Oxidation (M)
66 - 82	1679.8580	1678.8507	1678.8639	-8	0	K.VDDHIGVAIAGLTADGR.V
90 - 107	2134.9640	2133.9567	2133.9677	-5	0	R.SECINYSYTYESPLPVGR.L
122 - 136	1586.8800	1585.8727	1585.8828	-6	0	K.RPYGVGLLVAGLDEK.G
137 - 160	2785.2460	2784.2387	2784.2391	-0	0	K.GAHLYYNCPSGNYFEYQAFAIGSR.S
171 - 180	1272.5890	1271.5817	1271.5894	-6	1	R.RFENFSESTR.E
172 - 180	1116.4880	1115.4807	1115.4883	-7	0	R.FENFSESTR.E
202 - 226	2685.3340	2684.3267	2684.3480	-8	0	K.SSICTVAVVGAGEPFHILDQETVQK.L

100.
Match to: **clementine0.9_010729m|PAC:19260578** Score: **183** Expect: **6.7e-014**
Nominal mass (M_r): **48172**; Calculated pI value: **6.29**
NCBI BLAST search of clementine0.9_010729m|PAC:19260578 against nr
Unformatted sequence string for pasting into other applications

Fixed modifications: Carbamidomethyl (C)
Variable modifications: Oxidation (M)
Cleavage by Trypsin: cuts C-term side of KR unless next residue is P
Number of mass values matched: **21**
Sequence Coverage: **68%**

Matched peptides shown in **Bold Red**

```
  1 MAQILAPSMQ WQMRMPKYSN IASPMTTKMW SSLLMKQNKK GTNRSSAKFR
 51 VLALKSEDST VNRLEDLLNL DITPYTDKII AEYIWIGGTG IDMRSKSKTI
101 SKPVEHPSEL PKWNYDGSST GQAPGEDSEV ILYPQAIFKD PFRGGNNILV
151 ICDTYTPAGE PIPTNKRHRA AEIFSNSKVS AEVPWFGIEQ EYTLLQQNVK
201 WPLGWPVGAY PGPQGPYYCG AGADKSFGRD IADAHYKACL YAGINISGTN
251 GEVMPGQWEY QVGPSVGIDA GDHIWCSRYL LERITEQAGV VLSLDPKPIE
301 GDWNGAGCHT NYSTKSTREE GGYETIKKAI LNLSLRHKEH ISAYGEGNER
351 RLTGKHETAS IDSFSWGVAN RGCSIRVGRE TEKQGKGYLE DRRPASNMDP
401 YVVTSLLAET TILWEPTLEA EALAAQKLAL NV
```

Start - End	Observed	Mr(expt)	Mr(calc)	ppm	Miss	Sequence
1 - 17	2063.0540	2062.0467	2061.9984	23	1	-.MAQILAPSMQWQMRMPK.Y Oxidation (M)
1 - 17	2079.0660	2078.0587	2077.9933	31	1	-.MAQILAPSMQWQMRMPK.Y 2 Oxidation (M)
18 - 28	1228.6010	1227.5937	1227.5805	11	0	K.YSNIASPMTTK.M Oxidation (M)
29 - 39	1365.6500	1364.6427	1364.6944	-38	1	K.MWSSLLMKQNK.K
56 - 78	2651.3170	2650.3097	2650.2973	5	1	K.SEDSTVNRLEDLLNLDITPYTDK.I
64 - 78	1762.9230	1761.9157	1761.9036	7	0	R.LEDLLNLDITPYTDK.I
79 - 94	1807.9490	1806.9417	1806.9338	4	0	K.IIAEYIWIGGTGIDMR.S
79 - 94	1823.9490	1822.9417	1822.9287	7	0	K.IIAEYIWIGGTGIDMR.S Oxidation (M)
99 - 112	1561.8630	1560.8557	1560.8511	3	0	K.TISKPVEHPSELPK.W
113 - 139	2972.4250	2971.4177	2971.3876	10	0	K.WNYDGSSTGQAPGEDSEVILYPQAIFK.D
144 - 166	2444.2340	2443.2267	2443.2053	9	0	R.GGNNILVICDTYTPAGEPIPTNK.R
144 - 167	2600.3310	2599.3237	2599.3064	7	1	R.GGNNILVICDTYTPAGEPIPTNKR.H
179 - 200	2578.3420	2577.3347	2577.3115	9	0	K.VSAEVPWFGIEQEYTLLQQNVK.W
201 - 225	2664.2790	2663.2717	2663.2267	17	0	K.WPLGWPVGAYPGPQGPYYCGAGADK.S
284 - 315	3457.6860	3456.6787	3456.6620	5	0	R.ITEQAGVVLSLDPKPIEGDWNGAGCHTNYSTK.S
329 - 336	899.5700	898.5627	898.5600	3	0	K.AILNLSLR.H
337 - 350	1626.7740	1625.7667	1625.7546	7	1	R.HKEHISAYGEGNER.R
339 - 350	1361.6140	1360.6067	1360.6007	4	0	K.EHISAYGEGNER.R
356 - 371	1776.8470	1775.8397	1775.8227	10	0	K.HETASIDSFSWGVANR.G
393 - 427	3828.9990	3827.9917	3827.9655	7	0	R.RPASNMDPYVVTSLLAETTILWEPTLEAEALAAQK.L
393 - 427	3844.9890	3843.9817	3843.9604	6	0	R.RPASNMDPYVVTSLLAETTILWEPTLEAEALAAQK.L Oxidation (M)

101.
Match to: **clementine0.9_010912m|PAC:19284302** Score: **148** Expect: **2.1e-010**
Nominal mass (M_r): **47218**; Calculated pI value: **5.94**
NCBI BLAST search of clementine0.9_010912m|PAC:19284302 against nr
Unformatted sequence string for pasting into other applications

Fixed modifications: Carbamidomethyl (C)
Variable modifications: Oxidation (M)
Cleavage by Trypsin: cuts C-term side of KR unless next residue is P
Number of mass values matched: **26**
Sequence Coverage: **63%**

Matched peptides shown in **Bold Red**

```
  1 MAAAVPLSFN GSGAATSVPS SSFFGTSLKK VSSRIPPSKV PSASFKITAE
 51 VDENKQTKKD RWKGLAYDES DDQQDITRGK GAVDSLFQAP MGTGTHYAVM
101 SSYDYISQGL RTYNLDNTMD GLYIAPAFMD KLVVHITKNF MSLPNIKVPL
151 ILGIWGGKGQ GKSFQCELVF AKMGINPIMM SAGELESGNA GEPAKLIRQR
201 YREAADIIKK GKMCCLMIND LDAGAGRMGG TTQYTVNNQM VNATLMNIAD
251 NPTCVQLPGM YNKEENPRVP IIVTGNDFST LYAPLIRDGR MEKFYWAPTR
301 EDRIGVCKGI FRNDNVADDD IVKLVDTFPG QSIDFFGALR ARVYDDEVRK
351 WISGVGVGSI GKSLVNSKEA APTFEQPRMT MEKLLEYGNM IVQEQENVKR
401 VQLADKYLSE AALGEANEDA IQSGNFYG
```

Start - End	Observed	Mr(expt)	Mr(calc)	ppm	Miss	Sequence
64 - 78	1725.7680	1724.7607	1724.7489	7	0	K.GLAYDESDDQQDITR.G
81 - 111	3322.5810	3321.5737	3321.5435	9	0	K.GAVDSLFQAPMGTGTHYAVMSSYDYISQGLR.T
81 - 111	3338.5980	3337.5907	3337.5384	16	0	K.GAVDSLFQAPMGTGTHYAVMSSYDYISQGLR.T Oxidation (M)
81 - 111	3354.5720	3353.5647	3353.5333	9	0	K.GAVDSLFQAPMGTGTHYAVMSSYDYISQGLR.T 2 Oxidation (M)
112 - 131	2293.0680	2292.0607	2292.0442	7	0	R.TYNLDNTMDGLYIAPAFMDK.L
112 - 131	2309.0640	2308.0567	2308.0392	8	0	R.TYNLDNTMDGLYIAPAFMDK.L Oxidation (M)
112 - 131	2325.0630	2324.0557	2324.0341	9	0	R.TYNLDNTMDGLYIAPAFMDK.L 2 Oxidation (M)
148 - 158	1152.7170	1151.7097	1151.7067	3	0	K.VPLILGIWGGK.G
163 - 172	1228.6050	1227.5977	1227.5958	2	0	K.SFQCELVFAK.M
173 - 195	2304.0850	2303.0777	2303.0595	8	0	K.MGINPIMMSAGELESGNAGEPAK.L
173 - 195	2336.0730	2335.0657	2335.0494	7	0	K.MGINPIMMSAGELESGNAGEPAK.L 2 Oxidation (M)
173 - 195	2352.0740	2351.0667	2351.0443	10	0	K.MGINPIMMSAGELESGNAGEPAK.L 3 Oxidation (M)
213 - 227	1696.7370	1695.7297	1695.7201	6	0	K.MCCLMINDLDAGAGR.M
213 - 227	1712.7320	1711.7247	1711.7150	6	0	K.MCCLMINDLDAGAGR.M Oxidation (M)
213 - 227	1728.7190	1727.7117	1727.7099	1	0	K.MCCLMINDLDAGAGR.M 2 Oxidation (M)
228 - 263	3989.8320	3988.8247	3988.8250	-0	0	R.MGGTTQYTVNNQMVNATLMNIADNPTCVQLPGMYNK.E
269 - 287	2089.1880	2088.1807	2088.1619	9	0	R.VPIIVTGNDFSTLYAPLIR.D
294 - 300	940.4670	939.4597	939.4603	-1	0	K.FYWAPTR.E
324 - 340	1882.9840	1881.9767	1881.9625	8	0	K.LVDTFPGQSIDFFGALR.A
343 - 349	895.4190	894.4117	894.4083	4	0	R.VYDDEVR.K

343 - 350	1023.5020	1022.4947	1022.5033	-8	1	R.VYDDEVRK.W
351 - 362	1159.6410	1158.6337	1158.6397	-5	0	K.WISGVGVGSIGK.S
369 - 378	1145.5620	1144.5547	1144.5513	3	0	K.EAAPTFEQPR.M
384 - 400	2063.0710	2062.0637	2062.0517	6	1	K.LLEYGNMIVQEQENVKR.V
384 - 400	2079.0620	2078.0547	2078.0466	4	1	K.LLEYGNMIVQEQENVKR.V Oxidation (M)
407 - 428	2319.0670	2318.0597	2318.0338	11	0	K.YLSEAALGEANEDAIQSGNFYG.-

103.
Match to: **clementine0.9_011786m|PAC:19254460** Score: **141** Expect: **1.1e-009**
Nominal mass (M_r): **45558**; Calculated pI value: **5.97**
NCBI BLAST search of clementine0.9_011786m|PAC:19254460 against nr
Unformatted sequence string for pasting into other applications

Fixed modifications: Carbamidomethyl (C)
Variable modifications: Oxidation (M)
Cleavage by Trypsin: cuts C-term side of KR unless next residue is P
Number of mass values matched: **17**
Sequence Coverage: **61%**

Matched peptides shown in **Bold Red**

1 MAISLNSPCS LYSPTKTHLG FFNNQRQLAV FYNKRSSSSS SSSSSKQRGC
51 LVITCSAGDS QTIVIGLAAD SGCGKSTFMR R**LTSVFGGAA EPPKGGNPDS**
101 **NTLISETTTV ICLDDYHSLD R**TGRKEKGVT ALDPR**ANNFD LMYEQVK**AMK
151 **DGVSVEKPIY NHVTGLLDPP ELIKPPKILV IEGLHPMYDA R**VR**ELLDFSI**
201 **YLDISNEVK**F AWKIQRDMTE RGHSLESIK**A SIEARKPDFD AYIDPQKQYA**
251 **DAVIEVLPTQ LIPDDNEGK**V LRVRLIMKEG VK**YFSPVYLF DEGSTIEWIP**
301 **CGR**KLTCSYP GIK**FSYGPDA YFGHEVSILE MDGK**FDR**LDE LIYVESHLSN**
351 **LSTKFYGEVT QQMLKHADFP GSNNGTGLFQ TIVGLKIRDL YEQIITSK**AA
401 APVEAKA

Start - End	Observed	Mr(expt)	Mr(calc)	ppm	Miss	Sequence
82 - 94	1273.6380	1272.6307	1272.6714	-32	0	R.LTSVFGGAAEPPK.G
95 - 121	2993.4230	2992.4157	2992.3720	15	0	K.GGNPDSNTLISETTTVICLDDYHSLDR.T
136 - 147	1487.7090	1486.7017	1486.6762	17	0	R.ANNFDLMYEQVK.A Oxidation (M)
151 - 177	2955.6360	2954.6287	2954.6117	6	0	K.DGVSVEKPIYNHVTGLLDPPELIKPPK.I
178 - 191	1626.8710	1625.8637	1625.8599	2	0	K.ILVIEGLHPMYDAR.V
178 - 191	1642.8640	1641.8567	1641.8548	1	0	K.ILVIEGLHPMYDAR.V Oxidation (M)
194 - 209	1897.9690	1896.9617	1896.9720	-5	0	R.ELLDFSIYLDISNEVK.F
230 - 247	2064.0020	2062.9947	2063.0323	-18	1	K.ASIEARKPDFDAYIDPQK.Q
236 - 247	1436.7170	1435.7097	1435.6983	8	0	R.KPDFDAYIDPQK.Q
248 - 269	2428.2400	2427.2327	2427.2169	7	0	K.QYADAVIEVLPTQLIPDDNEGK.V
283 - 303	2536.2180	2535.2107	2535.1781	13	0	K.YFSPVYLFDEGSTIEWIPCGR.K
314 - 334	2378.1470	2377.1397	2377.0573	35	0	K.FSYGPDAYFGHEVSILEMDGK.F Oxidation (M)
338 - 354	1961.0070	1959.9997	1960.0153	-8	0	R.LDELIYVESHLSNLSTK.F
355 - 365	1343.6680	1342.6607	1342.6591	1	0	K.FYGEVTQQMLK.H

355 - 365	1359.6690	1358.6617	1358.6540	6	0	K.FYGEVTQQMLK.H Oxidation (M)
366 - 386	2173.1090	2172.1017	2172.0964	2	0	K.HADFPGSNNGTGLFQTIVGLK.I
387 - 398	1478.8210	1477.8137	1477.8140	-0	1	K.IRDLYEQIITSK.A

105.
Match to: **clementine0.9_010729m|PAC:19260578** Score: **218** Expect: **2.1e-017**
Nominal mass (M_r): **48172**; Calculated pI value: **6.29**
NCBI BLAST search of clementine0.9_010729m|PAC:19260578 against nr
Unformatted sequence string for pasting into other applications

Fixed modifications: Carbamidomethyl (C)
Variable modifications: Oxidation (M)
Cleavage by Trypsin: cuts C-term side of KR unless next residue is P
Number of mass values matched: **17**
Sequence Coverage: **59%**

Matched peptides shown in **Bold Red**

```
  1 MAQILAPSMQ WQMRMPKYSN IASPMTTKMW SSLLMKQNKK GTNRSSAKFR
 51 VLALKSEDST VNRLEDLLNL DITPYTDKII AEYIWIGGTG IDMRSKSKTI
101 SKPVEHPSEL PKWNYDGSST GQAPGEDSEV ILYPQAIFKD PFRGGNNILV
151 ICDTYTPAGE PIPTNKRHRA AEIFSNSKVS AEVPWFGIEQ EYTLLQQNVK
201 WPLGWPVGAY PGPQGPYYCG AGADKSFGRD IADAHYKACL YAGINISGTN
251 GEVMPGQWEY QVGPSVGIDA GDHIWCSRYL LERITEQAGV VLSLDPKPIE
301 GDWNGAGCHT NYSTKSTREE GGYETIKKAI LNLSLRHKEH ISAYGEGNER
351 RLTGKHETAS IDSFSWGVAN RGCSIRVGRE TEKQGKGYLE DRRPASNMDP
401 YVVTSLLAET TILWEPTLEA EALAAQKLAL NV
```

Start - End	Observed	Mr(expt)	Mr(calc)	ppm	Miss	Sequence
56 - 78	2651.3220	2650.3147	2650.2973	7	1	K.SEDSTVNRLEDLLNLDITPYTDK.I
64 - 78	1762.9230	1761.9157	1761.9036	7	0	R.LEDLLNLDITPYTDK.I
79 - 94	1807.9530	1806.9457	1806.9338	7	0	K.IIAEYIWIGGTGIDMR.S
79 - 94	1823.9510	1822.9437	1822.9287	8	0	K.IIAEYIWIGGTGIDMR.S Oxidation (M)
99 - 112	1561.8660	1560.8587	1560.8511	5	0	K.TISKPVEHPSELPK.W
113 - 139	2972.4250	2971.4177	2971.3876	10	0	K.WNYDGSSTGQAPGEDSEVILYPQAIFK.D
144 - 166	2444.2330	2443.2257	2443.2053	8	0	R.GGNNILVICDTYTPAGEPIPTNK.R
144 - 167	2600.3390	2599.3317	2599.3064	10	1	R.GGNNILVICDTYTPAGEPIPTNKR.H
179 - 200	2578.3400	2577.3327	2577.3115	8	0	K.VSAEVPWFGIEQEYTLLQQNVK.W
201 - 225	2664.2650	2663.2577	2663.2267	12	0	K.WPLGWPVGAYPGPQGPYYCGAGADK.S
284 - 315	3457.6940	3456.6867	3456.6620	7	0	R.ITEQAGVVLSLDPKPIEGDWNGAGCHTNYSTK.S
329 - 336	899.5690	898.5617	898.5600	2	0	K.AILNLSLR.H
337 - 350	1626.7730	1625.7657	1625.7546	7	1	R.HKEHISAYGEGNER.R
339 - 350	1361.6140	1360.6067	1360.6007	4	0	K.EHISAYGEGNER.R
356 - 371	1776.8480	1775.8407	1775.8227	10	0	K.HETASIDSFSWGVANR.G
393 - 427	3829.0000	3827.9927	3827.9655	7	0	R.RPASNMDPYVVTSLLAETTILWEPTLEAEALAAQK.L

393 - 427	3845.0020	3843.9947	3843.9604	9	0	R.RPASNMDPYVVTSLLAETTILWEPTLEAEALAAQK.L Oxidation (M)

106.
Match to: **orange1.1g008638m|PAC:18095844** Score: **152** Expect: **8.5e-011**
Nominal mass (M_r): **59862**; Calculated pI value: **6.06**
NCBI BLAST search of orange1.1g008638m|PAC:18095844 against nr
Unformatted sequence string for pasting into other applications

Fixed modifications: Carbamidomethyl (C)
Variable modifications: Oxidation (M)
Cleavage by Trypsin: cuts C-term side of KR unless next residue is P
Number of mass values matched: **22**
Sequence Coverage: **51%**

Matched peptides shown in **Bold Red**

```
  1 MASRRLLSSL LRSSVRRSPS KSSLSNSSVF SPAATRRASP YGHLLSRVSE
 51 YATSAAATAP PAQTPKSDVK KGGGGKITDE FTGKGAIGQV CQVIGAVVDV
101 RFDEGLPPIL TALEVVDHSV RLVLEVAQHM GEGVVRTIAM DGTEGLVRGQ
151 RVLNTGSPIT VPVGRVTLGR IMNVIGEPID EKGDLKTEHY LPIHREAPAF
201 VEQATEQQIL VTGIKVVDLL APYQRGGKIG LFGGAGVGKT VLIMELINNV
251 AKAHGGFSVF AGVGERTREG NDLYREMIES GVIKLGDKQA DSKCALVYGQ
301 MNEPPGARAR VGLTGLTVAE HFRDAEGQDV LLFIDNIFRF TQANSEVSAL
351 LGRIPSAVGY QPTLATDLGG LQERITTTKK GSITSVQAIY VPADDLTDPA
401 PATTFAHLDA TTVLSRQISE LGIYPAVDPL DSTSRMLSPH ILGEEHYNTA
451 RGVQKVLQNY KNLQDIIAIL GMDELSEDDK LTVARARKIQ RFLSQPFHVA
501 EVFTGAPGKY VELKESIASF QGVLDGKYDD LPEQSFYMVG GIEEVIAKAE
551 KIAKESAA
```

Start - End	Observed	Mr(expt)	Mr(calc)	ppm	Miss	Sequence
85 - 101	1740.9450	1739.9377	1739.9353	1	0	K.GAIGQVCQVIGAVVDVR.F
102 - 121	2207.1760	2206.1687	2206.1634	2	0	R.FDEGLPPILTALEVVDHSVR.L
102 - 136	3824.9000	3823.8927	3824.0295	-36	1	R.FDEGLPPILTALEVVDHSVRLVLEVAQHMGEGVVR.T
122 - 136	1636.8760	1635.8687	1635.8767	-5	0	R.LVLEVAQHMGEGVVR.T
122 - 136	1652.8870	1651.8797	1651.8716	5	0	R.LVLEVAQHMGEGVVR.T Oxidation (M)
152 - 165	1409.8110	1408.8037	1408.8038	-0	0	R.VLNTGSPITVPVGR.V
187 - 195	1165.6100	1164.6027	1164.6040	-1	0	K.TEHYLPIHR.E
216 - 225	1173.6640	1172.6567	1172.6554	1	0	K.VVDLLAPYQR.G
253 - 266	1390.6940	1389.6867	1389.6790	6	0	K.AHGGFSVFAGVGER.T
269 - 284	1868.9950	1867.9877	1867.8985	48	1	R.EGNDLYREMIESGVIK.L Oxidation (M)
276 - 284	1021.5370	1020.5297	1020.5161	13	0	R.EMIESGVIK.L Oxidation (M)
294 - 308	1662.7830	1661.7757	1661.7654	6	0	K.CALVYGQMNEPPGAR.A
294 - 308	1678.8030	1677.7957	1677.7603	21	0	K.CALVYGQMNEPPGAR.A Oxidation (M)
311 - 323	1399.7720	1398.7647	1398.7620	2	0	R.VGLTGLTVAEHFR.D
324 - 339	1864.9550	1863.9477	1863.9367	6	0	R.DAEGQDVLLFIDNIFR.F

Start - End	Observed	Mr(expt)	Mr(calc)	ppm	Miss	Sequence
340 - 353	1492.7810	1491.7737	1491.7681	4	0	R.FTQANSEVSALLGR.I
354 - 374	2186.1500	2185.1427	2185.1379	2	0	R.IPSAVGYQPTLATDLGGLQER.I
381 - 416	3714.9060	3713.8987	3713.8789	5	0	K.GSITSVQAIYVPADDLTDPAPATTFAHLDATTVLSR.Q
417 - 435	2061.0530	2060.0457	2060.0426	2	0	R.QISELGIYPAVDPLDSTSR.M
462 - 485	2672.3730	2671.3657	2671.3738	-3	1	K.NLQDIIAILGMDELSEDDKLTVAR.A
462 - 485	2688.3890	2687.3817	2687.3687	5	1	K.NLQDIIAILGMDELSEDDKLTVAR.A Oxidation (M)
515 - 527	1350.6630	1349.6557	1349.6827	-20	0	K.ESIASFQGVLDGK.Y

111.
Match to: **clementine0.9_004603m|PAC:19260210** Score: **75** Expect: **0.0045**
Nominal mass (M_r): **71381**; Calculated pI value: **5.11**
NCBI BLAST search of clementine0.9_004603m|PAC:19260210 against nr
Unformatted sequence string for pasting into other applications

Fixed modifications: Carbamidomethyl (C)
Variable modifications: Oxidation (M)
Cleavage by Trypsin: cuts C-term side of KR unless next residue is P
Number of mass values matched: **9**
Sequence Coverage: **21%**

Matched peptides shown in **Bold Red**

```
  1 MAGKGEGPAI GIDLGTTYSC VGVWQHDRVE IIANDQGNRT TPSYVAFTDT
 51 ERLIGDAAKN QVAMNPTNTV FDAKRLIGRR FSDPSVQSDM KLWPFKVIAG
101 AGDKPMIVVN YKGEEKQFSA EEISSMVLNK MKEIAEAYLG TTIKNAVVTV
151 PAYFNDSQRQ ATKDAGVISG LNVMRIINEP TAAAIAYGLD KKAGSTGEKN
201 VLIFDLGGGT FDVSLLTIEE GIFEVKATAG DTHLGGEDFD NRMVNHFVQE
251 FKRKNKKDIS GNPRALRRLR TACERAKRTL SSTAQTTIEI DSLYEGIDFY
301 TTITRARFEE LNMDLFRKCM EPVEKCLRDA KMDKSSVHDV VLVGGSTRIP
351 KVQQLLQDFF NGKELCKSIN PDEAVAYGAA VQAAILSGEG NEKVQDLLLL
401 DVTPLSLGLE TAGGVMTVLI PRNTTIPTKK EQVFSTYSDN QPGVLIQVYE
451 GERTRTRDNN LLGKFELSGI PPAPRGVPQI NVCFDIDANG ILNVSAEDKT
501 TGQKNKITIT NDKGRLSKEE IEKMVQEAEK YKAEDDEHKK KVEAKNALEN
551 YAYNMRNTIK DEKISAKLPT ADKKKIEDAI EEAIQWLDGN QLAEADEFED
601 KMKELEGICN PIIAKMYQGG GADAGASMDE DGPSAGAGSG AGPKIEEVD
```

Start - End	Observed	Mr(expt)	Mr(calc)	ppm	Miss	Sequence
29 - 39	1228.6600	1227.6527	1227.6207	26	0	R.VEIIANDQGNR.T
40 - 52	1487.7340	1486.7267	1486.6940	22	0	R.TTPSYVAFTDTER.L
133 - 144	1308.6910	1307.6837	1307.6972	-10	0	K.EIAEAYLGTTIK.N
145 - 159	1680.8690	1679.8617	1679.8267	21	0	K.NAVVTVPAYFNDSQR.Q
227 - 242	1675.7840	1674.7767	1674.7234	32	0	K.ATAGDTHLGGEDFDNR.M
308 - 317	1329.6630	1328.6557	1328.6071	37	0	R.FEELNMDLFR.K Oxidation (M)
335 - 348	1412.7790	1411.7717	1411.7420	21	0	K.SSVHDVVLVGGSTR.I
431 - 453	2658.3210	2657.3137	2657.2609	20	0	K.EQVFSTYSDNQPGVLIQVYEGER.T

476 - 499	2588.2810	2587.2737	2587.2588	6	0	R.GVPQINVCFDIDANGILNVSAEDK.T

112.
Match to: **clementine0.9_002637m|PAC:19282329** Score: **265** Expect: **4.3e-022**
Nominal mass (M_r): **90244**; Calculated pI value: **5.14**
NCBI BLAST search of clementine0.9_002637m|PAC:19282329 against nr
Unformatted sequence string for pasting into other applications

Fixed modifications: Carbamidomethyl (C)
Variable modifications: Oxidation (M)
Cleavage by Trypsin: cuts C-term side of KR unless next residue is P
Number of mass values matched: **27**
Sequence Coverage: **47%**

Matched peptides shown in **Bold Red**

```
  1 MSHPAESSDA NSGKKDYSTA ILERKKSPNR LVVDEAINDD NSVVGLHPDT
 51 MDKLQIFRGD TILIKGKKRK DTICIALADD TCEQPKIRMN KVVRSNLRVR
101 LGDVVSVHQC PDVKYGKRVH ILPIDDTIEG VTGNLFDAFL RPYFTEAYRP
151 VRKGDLFLVR GGMRSVEFKV IETDPGEYCV VAPDTEIFCE GEPIKREDED
201 RLDEVGYDDV GGVRKQMAQI RELVELPLRH PQLFKSIGVK PPKGILLYGP
251 PGSGKTLIAR AVANETGAFF FCINGPEIMS KLAGESESNL RKAFEEAEKN
301 APSIIFIDEI DSIAPKREKT HGEVERRIVS QLLTLMDGLK SRAHVIVIGA
351 TNRPNSIDPA LRRFGRFDRE IDIGVPDEVG RLEVLRIHTK NMKLSDDVDL
401 ERIAKDTHGY VGADLAALCT EAALQCIREK MDVIDLEDET IDAEILNSMA
451 VSNEHFQTAL GTSNPSALRE TVVEVPNVSW EDIGGLENVK RELQETVQYP
501 VEHPEKFEKF GMSPSKGVLF YGPPGCGKTL LAKAIANECQ ANFISVKGPE
551 LLTMWFGESE ANVREIFDKA RQSAPCVLFF DELDSIATQR GSSVGDAGGA
601 ADRVLNQLLT EMDGMSAKKT VFIIGATNRP DIIDPALLRP GRLDQLIYIP
651 LPDEDSRHQI FKACLRKSPI SKDVDLRALA KYTQGFSGAD ITEICQRACK
701 YAIRENIEKD IERERRRDN PEAMDEDAAE DEVAEIKAAH FEESMKFARR
751 SVSDADIRKY QAFAQTLQQS RGIGSEFRFA EAGTGATTGA DPFSTSAGGA
801 DDDDLYS
```

Start - End	Observed	Mr(expt)	Mr(calc)	ppm	Miss	Sequence
31 - 53	2512.2100	2511.2027	2511.1799	9	0	R.LVVDEAINDDNSVVGLHPDTMDK.L Oxidation (M)
101 - 114	1552.7800	1551.7727	1551.7716	1	0	R.LGDVVSVHQCPDVK.Y
119 - 152	3935.0540	3934.0467	3934.0418	1	0	R.VHILPIDDTIEGVTGNLFDAFLRPYFTEAYRPVR.K
153 - 160	947.5740	946.5667	946.5600	7	1	R.KGDLFLVR.G
170 - 196	3123.4960	3122.4887	3122.4577	10	1	K.VIETDPGEYCVVAPDTEIFCEGEPIKR.E
197 - 214	2037.9250	2036.9177	2036.8923	12	1	R.EDEDRLDEVGYDDVGGVR.K
222 - 229	968.5820	967.5747	967.5702	5	0	R.ELVELPLR.H
244 - 255	1158.6330	1157.6257	1157.6445	-16	0	K.GILLYGPPGSGK.T
261 - 281	2319.1260	2318.1187	2318.0711	21	0	R.AVANETGAFFFCINGPEIMSK.L Oxidation (M)
327 - 340	1586.8650	1585.8577	1585.9225	-41	1	R.RIVSQLLTLMDGLK.S
328 - 340	1446.8070	1445.7997	1445.8163	-11	0	R.IVSQLLTLMDGLK.S Oxidation (M)

**343 - 362**	**2114.1960**	**2113.1887**	**2113.1756**	**6**	**0**	**R.AHVIVIGATNRPNSIDPALR.R**	
**367 - 381**	**1716.8650**	**1715.8577**	**1715.8479**	**6**	**1**	**R.FDREIDIGVPDEVGR.L**	
**370 - 381**	**1298.6630**	**1297.6557**	**1297.6514**	**3**	**0**	**R.EIDIGVPDEVGR.L**	
**470 - 490**	**2313.1800**	**2312.1727**	**2312.1536**	**8**	**0**	**R.ETVVEVPNVSWEDIGGLENVK.R**	
**492 - 506**	**1825.9080**	**1824.9007**	**1824.8894**	**6**	**0**	**R.ELQETVQYPVEHPEK.F**	
**492 - 509**	**2230.2070**	**2229.1997**	**2229.0953**	**47**	**1**	**R.ELQETVQYPVEHPEKFEK.F**	
**517 - 528**	**1251.6220**	**1250.6147**	**1250.6118**	**2**	**0**	**K.GVLFYGPPGCGK.T**	
**534 - 547**	**1564.7770**	**1563.7697**	**1563.7715**	**-1**	**0**	**K.AIANECQANFISVK.G**	
**548 - 564**	**1951.9410**	**1950.9337**	**1950.9146**	**10**	**0**	**K.GPELLTMWFGESEANVR.E**	Oxidation (M)
**572 - 590**	**2197.0860**	**2196.0787**	**2196.0521**	**12**	**0**	**R.QSAPCVLFFDELDSIATQR.G**	
**619 - 642**	**2633.5200**	**2632.5127**	**2632.5177**	**-2**	**1**	**K.KTVFIIGATNRPDIIDPALLRPGR.L**	
**620 - 642**	**2505.3220**	**2504.3147**	**2504.4227**	**-43**	**0**	**K.TVFIIGATNRPDIIDPALLRPGR.L**	
**643 - 657**	**1786.9360**	**1785.9287**	**1785.9149**	**8**	**0**	**R.LDQLIYIPLPDEDSR.H**	
**682 - 697**	**1845.8670**	**1844.8597**	**1844.8363**	**13**	**0**	**K.YTQGFSGADITEICQR.A**	
**759 - 771**	**1568.8230**	**1567.8157**	**1567.8107**	**3**	**1**	**R.KYQAFAQTLQQSR.G**	
**760 - 771**	**1440.7300**	**1439.7227**	**1439.7157**	**5**	**0**	**K.YQAFAQTLQQSR.G**	

113.
Match to: **clementine0.9_005361m|PAC:19265639** Score: **211** Expect: **1.1e-016**
Nominal mass (M_r): **65086**; Calculated pI value: **5.85**
NCBI BLAST search of clementine0.9_005361m|PAC:19265639 against nr
Unformatted sequence string for pasting into other applications

Fixed modifications: Carbamidomethyl (C)
Variable modifications: Oxidation (M)
Cleavage by Trypsin: cuts C-term side of KR unless next residue is P
Number of mass values matched: **25**
Sequence Coverage: **50%**

Matched peptides shown in **Bold Red**

1 MASTFTAMSS ISSMIAPNGR MTDKKLASSS NKLSSLASIS SSSFGRRQSV
51 ALRRARTPKI YAAKDLHFNK DGYAMKKLQN GVNK**LADLVG** **VTLGPK**GRNV
101 VLESKYGAPK IVNDGVTVAK **EVELEDPVEN** **IGAK**LVRQAA AK**TNDLAGDG**
151 **TTTSVVLAQG** **LIAEGVKVVA** **AGANPVLITR** GIEKTSKALV SELKQMSK**EV**
201 **EDSELADVAA** **VSAGNNYEVG** **NMIAEAMSK**V GRKGVVTLEE GK**SAENMLYV**
251 **VEGMQFDRGY** **ISPYFVTDSE** **KMAVEYENCK** **LLLVDK**KITN AR**DLINVLED**
301 **AIRGAYPILI** **IAEDIEQEAL** **ATLVVNK**LRG ALKIAALKAP GFGER**KSQYL**
351 **DDIAILTGGT** **VIRDEVGLAL** **DK**VGKEVLGN ASKVVLTKDT TTIVGDGSTQ
401 DAVSKRVAQI R**TLIENAEQD** **YER**EKLNERI AK**LSGGVAVI** **QVGAQTETEL**
451 **KEK**KLRVEDA LNATK**AAVEE** **GIVVGGGCTL** **LR**LSSKVDAI KETLDNDEEK
501 VGADIVKRAL CYPLKLIAKN AGVNGSVVSE KVLSSDNHK**Y** **GYNAATGNYE**
551 **DLMAAGIIDP** **TK**VVRCCLEH ASSVAK**TFLM** **SDCVVVEIKE** **PEPAMPAGNP**
601 **MDNSGYGY**

Start - End	Observed	Mr(expt)	Mr(calc)	ppm	Miss	Sequence
85 - 96	1182.7130	1181.7057	1181.7020	3	0	K.LADLVGVTLGPK.G
121 - 134	1541.7770	1540.7697	1540.7620	5	0	K.EVELEDPVENIGAK.L
143 - 167	2430.2930	2429.2857	2429.2650	9	0	K.TNDLAGDGTTTSVVLAQGLIAEGVK.V
168 - 180	1280.7760	1279.7687	1279.7612	6	0	K.VVAAGANPVLITR.G
199 - 229	3229.4830	3228.4757	3228.4438	10	0	K.EVEDSELADVAAVSAGNNYEVGNMIAEAMSK.V Oxidation (M)
199 - 229	3245.4750	3244.4677	3244.4387	9	0	K.EVEDSELADVAAVSAGNNYEVGNMIAEAMSK.V 2 Oxidation (M)
243 - 258	1888.8740	1887.8667	1887.8495	9	0	K.SAENMLYVVEGMQFDR.G
243 - 258	1904.8710	1903.8637	1903.8444	10	0	K.SAENMLYVVEGMQFDR.G Oxidation (M)
243 - 258	1920.8680	1919.8607	1919.8393	11	0	K.SAENMLYVVEGMQFDR.G 2 Oxidation (M)
259 - 271	1505.7290	1504.7217	1504.7086	9	0	R.GYISPYFVTDSEK.M
272 - 286	1840.8760	1839.8687	1839.9110	-23	1	K.MAVEYENCKLLLVDK.K Oxidation (M)
293 - 303	1270.7110	1269.7037	1269.6928	9	0	R.DLINVLEDAIR.G
304 - 327	2583.4420	2582.4347	2582.4207	5	0	R.GAYPILIIAEDIEQEALATLVVNK.L
346 - 363	1963.0950	1962.0877	1962.0786	5	1	R.KSQYLDDIAILTGGTVIR.D
347 - 363	1835.0070	1833.9997	1833.9836	9	0	K.SQYLDDIAILTGGTVIR.D
347 - 372	2775.5040	2774.4967	2774.4702	10	1	K.SQYLDDIAILTGGTVIRDEVGLALDK.V
412 - 423	1480.7040	1479.6967	1479.6841	9	0	R.TLIENAEQDYER.E
433 - 451	1900.0490	1899.0417	1899.0313	5	0	K.LSGGVAVIQVGAQTETELK.E
433 - 453	2157.1710	2156.1637	2156.1689	-2	1	K.LSGGVAVIQVGAQTETELKEK.K
466 - 482	1700.9170	1699.9097	1699.8927	10	0	K.AAVEEGIVVGGGCTLLR.L
540 - 562	2448.1780	2447.1707	2447.1315	16	0	K.YGYNAATGNYEDLMAAGIIDPTK.V
540 - 562	2464.1590	2463.1517	2463.1264	10	0	K.YGYNAATGNYEDLMAAGIIDPTK.V Oxidation (M)
577 - 589	1556.7820	1555.7747	1555.7626	8	0	K.TFLMSDCVVVEIK.E Oxidation (M)
577 - 608	3550.6000	3549.5927	3549.5448	13	1	K.TFLMSDCVVVEIKEPEPAMPAGNPMDNSGYGY.- 2 Oxidation (M)
577 - 608	3566.6120	3565.6047	3565.5397	18	1	K.TFLMSDCVVVEIKEPEPAMPAGNPMDNSGYGY.- 3 Oxidation (M)

116.
Match to: **orange1.1g005304m|PAC:18127398** Score: **197** Expect: **2.7e-015**
Nominal mass (M_r): **75921**; Calculated pI value: **6.44**
NCBI BLAST search of orange1.1g005304m|PAC:18127398 against nr
Unformatted sequence string for pasting into other applications

Fixed modifications: Carbamidomethyl (C)
Variable modifications: Oxidation (M)
Cleavage by Trypsin: cuts C-term side of KR unless next residue is P
Number of mass values matched: **28**
Sequence Coverage: **51%**

Matched peptides shown in **Bold Red**

```
  1 MEASSACLVG NILTTHKTKT NLSKDFHGRH LLFNSSFRSL EKKPKVAVVK
 51 ASLSQKQHEG RRGFLKKLVG NVGVGTALLG SGKAYADEQG VSSSRMSYSR
101 FLEYLDKDRV KKVDLFENGT IAIVEAISPE LGNRVQRVRV QLPGLSQELL
151 QKFREKNIDF AAHNAQEDSG SLLFNLIGNL AFPLILIGGL FLLSRRSSGG
201 MGGPGGPGFP LAFGQSKAKF QMEPNTGVTF DDVAGVDEAK QDFMEVVEFL
```

```
251 KKPERFTAIG ARIPKGVLLV GPPGTGKTLL AKAIAGEAGV PFFSISGSEF
301 VEMFVGVGAS RVRDLFKKAK ENAPCIVFVD EIDAVGRQRG TGIGGGNDER
351 EQTLNQLLTE MDGFEGNTGI IVIAATNRAD ILDSALLRPG RFDRQVTVDV
401 PDIRGRTEIL KVHGSNKKFD ADVSLDVIAM RTPGFSGADL ANLLNEAAIL
451 AGRRGKAAIS SKEIDDSIDR IVAGMEGTVM TDGKSKSLVA YHEVGHAICG
501 TLTPGHDPVQ KVTLVPRGQA RGLTWFIPSD DPTLISKQQL FARIVGGLGG
551 RAAEEVIFGE PEVTTGAAGD LQQITGLAKQ AHYFFFFLQM VTTFGMSEIG
601 PWSLMDGSQS GDVIMRMMAR NSMSEKLAED IDAAVKRLSD RAYEIALSQI
651 RNNREAIDKI VEVLLEKETM SGDEFRAILS EFVEIPAENR VPPAVPAPLS
701 VSV
```

Start - End	Observed	Mr(expt)	Mr(calc)	ppm	Miss	Sequence
2 - 17	1700.8790	1699.8717	1699.8563	9	0	M.EASSACLVGNILTTHK.T
51 - 61	1240.6690	1239.6617	1239.6320	24	1	K.ASLSQKQHEGR.R
96 - 107	1567.7730	1566.7657	1566.7388	17	1	R.MSYSRFLEYLDK.D Oxidation (M)
101 - 109	1198.6200	1197.6127	1197.6030	8	1	R.FLEYLDKDR.V
112 - 134	2485.3420	2484.3347	2484.3224	5	1	K.KVDLFENGTIAIVEAISPELGNR.V
113 - 134	2357.2300	2356.2227	2356.2274	-2	0	K.VDLFENGTIAIVEAISPELGNR.V
140 - 152	1452.8230	1451.8157	1451.8348	-13	0	R.VQLPGLSQELLQK.F
197 - 217	1908.9180	1907.9107	1907.8836	14	0	R.SSGGMGGPGGPGFPLAFGQSK.A Oxidation (M)
241 - 251	1400.6940	1399.6867	1399.6694	12	0	K.QDFMEVVEFLK.K Oxidation (M)
283 - 311	2918.4450	2917.4377	2917.4321	2	0	K.AIAGEAGVPFFSISGSEFVEMFVGVGASR.V
283 - 311	2934.4680	2933.4607	2933.4270	12	0	K.AIAGEAGVPFFSISGSEFVEMFVGVGASR.V Oxidation (M)
319 - 337	2103.0710	2102.0637	2102.0466	8	1	K.AKENAPCIVFVDEIDAVGR.Q
321 - 337	1903.9400	1902.9327	1902.9146	10	0	K.ENAPCIVFVDEIDAVGR.Q
351 - 378	3048.5380	3047.5307	3047.5233	2	0	R.EQTLNQLLTEMDGFEGNTGIIVIAATNR.A
351 - 378	3064.5470	3063.5397	3063.5183	7	0	R.EQTLNQLLTEMDGFEGNTGIIVIAATNR.A Oxidation (M)
379 - 391	1396.7970	1395.7897	1395.7834	5	0	R.ADILDSALLRPGR.F
395 - 404	1141.6260	1140.6187	1140.6139	4	0	R.QVTVDVPDIR.G
418 - 431	1579.8140	1578.8067	1578.8076	-1	1	K.KFDADVSLDVIAMR.T
418 - 431	1595.8340	1594.8267	1594.8025	15	1	K.KFDADVSLDVIAMR.T Oxidation (M)
419 - 431	1467.7410	1466.7337	1466.7075	18	0	K.FDADVSLDVIAMR.T Oxidation (M)
432 - 453	2171.1640	2170.1567	2170.1382	9	0	R.TPGFSGADLANLLNEAAILAGR.R
463 - 484	2384.0070	2382.9997	2383.0883	-37	1	K.EIDDSIDRIVAGMEGTVMTDGK.S 2 Oxidation (M)
471 - 486	1639.8710	1638.8637	1638.7957	42	1	R.IVAGMEGTVMTDGKSK.S Oxidation (M)
487 - 511	2686.3780	2685.3707	2685.3333	14	0	K.SLVAYHEVGHAICGTLTPGHDPVQK.V
522 - 537	1789.9290	1788.9217	1788.9298	-5	0	R.GLTWFIPSDDPTLISK.Q
552 - 579	2815.4520	2814.4447	2814.4287	6	0	R.AAEEVIFGEPEVTTGAAGDLQQITGLAK.Q
642 - 651	1163.6470	1162.6397	1162.6346	4	0	R.AYEIALSQIR.N
677 - 690	1587.8460	1586.8387	1586.8304	5	0	R.AILSEFVEIPAENR.V

121.
Match to: **clementine0.9_024051m|PAC:19279711** Score: **119** Expect: **1.7e-007**
Nominal mass (M_r): **17443**; Calculated pI value: **5.15**
NCBI BLAST search of clementine0.9_024051m|PAC:19279711 against nr
Unformatted sequence string for pasting into other applications

Fixed modifications: Carbamidomethyl (C)
Variable modifications: Oxidation (M)
Cleavage by Trypsin: cuts C-term side of KR unless next residue is P
Number of mass values matched: **7**
Sequence Coverage: **70%**

Matched peptides shown in **Bold Red**

```
  1 MAPIAVGDPL PDGTLAYFDE QDQLQQVSVH SLAAGKKVIL FGVPGAFTPT
 51 CSLKHVPGFI EKADELNSKG VDEILCISVN DPFVMKAWAK TFPENKSMKF
101 LADGSAKYTH ALGLELDLSE KGLGTRSRRF ALLVDDLKVK AANVESGGDF
151 TVSSADDILK AL
```

Start - End	Observed	Mr(expt)	Mr(calc)	ppm	Miss	Sequence
2 - 36	3650.8790	3649.8717	3649.8264	12	0	M.APIAVGDPLPDGTLAYFDEQDQLQQVSVHSLAAGK.K
37 - 54	1935.0450	1934.0377	1934.0700	-17	1	K.KVILFGVPGAFTPTCSLK.H
38 - 54	1807.0000	1805.9927	1805.9750	10	0	K.VILFGVPGAFTPTCSLK.H
70 - 86	1951.9800	1950.9727	1950.9431	15	0	K.GVDEILCISVNDPFVMK.A Oxidation (M)
108 - 121	1588.8410	1587.8337	1587.8144	12	0	K.YTHALGLELDLSEK.G
129 - 138	1189.7050	1188.6977	1188.6866	9	1	R.RFALLVDDLK.V
141 - 160	1995.9780	1994.9707	1994.9433	14	0	K.AANVESGGDFTVSSADDILK.A

122.
Match to: **clementine0.9_024784m|PAC:19280181** Score: **48** Expect: **1.9**
Nominal mass (M_r): **16962**; Calculated pI value: **6.07**
NCBI BLAST search of clementine0.9_024784m|PAC:19280181 against nr
Unformatted sequence string for pasting into other applications

Fixed modifications: Carbamidomethyl (C)
Variable modifications: Oxidation (M)
Cleavage by Trypsin: cuts C-term side of KR unless next residue is P
Number of mass values matched: **4**
Sequence Coverage: **40%**

Matched peptides shown in **Bold Red**

```
  1 MRRKCSIALL SPTPWSTCAR CFLHEHVLVC SRREAKRFAD LTADETTDLW
 51 LTAQKVGKQL ESYHKGSSTT FAIQDGPEAG QTVPHVHIHV LPRKAGDFEK
101 NDEIYDAIEV KEKELQQKLD LDKERKDRSL EEMNQEADQY RSLFL
```

Start - End	Observed	Mr(expt)	Mr(calc)	ppm	Miss	Sequence
38 - 55	2039.0100	2038.0027	2037.9895	6	0	R.FADLTADETTDLWLTAQK.V
66 - 93	2951.5530	2950.5457	2950.5050	14	0	K.GSSTTFAIQDGPEAGQTVPHVHIHVLPR.K
129 - 141	1612.7190	1611.7117	1611.6835	18	0	R.SLEEMNQEADQYR.S
129 - 141	1628.7010	1627.6937	1627.6784	9	0	R.SLEEMNQEADQYR.S Oxidation (M)

124.
Match to: **clementine0.9_015646m|PAC:19271726** Score: **110** Expect: **1.3e-006**
Nominal mass (M_r): **36735**; Calculated pI value: **4.81**
NCBI BLAST search of clementine0.9_015646m|PAC:19271726 against nr
Unformatted sequence string for pasting into other applications

Fixed modifications: Carbamidomethyl (C)
Variable modifications: Oxidation (M)
Cleavage by Trypsin: cuts C-term side of KR unless next residue is P
Number of mass values matched: **11**
Sequence Coverage: **45%**

Matched peptides shown in **Bold Red**

```
  1 MRLIGSLLIF SLVLSFVLGG SAGNCGSGVV CPGGECCSRF GWCGLTTDYC
 51 CEGCQSNCNQ VVCGECDPDD GTAGDGGELG KIISRKMFED LLEYRNDKRC
101 PARCFYTYDA FIEAAKAFPA FGNSGNETMR KREIAAFFAQ TGHETTGGWP
151 DAPGGEYAWG YCFNREVGAA SSDYCDPNYP CRGKYYGRGP IQLSWNYNYL
201 RCGEGLGLGE ELLNNPDLLA TDPVLSFKSA IWFWMTAQPP KPSCHEVIID
251 EWKPSANDVN AGRLPGYGLT TNIINGGIEC GYVGNDAVRN RIGFFTTFCG
301 KFGIQPGDNL DCSNQRPYGL NLMAQSM
```

Start - End	Observed	Mr(expt)	Mr(calc)	ppm	Miss	Sequence
**86 - 95**	**1343.6790**	**1342.6717**	**1342.6591**	**9**	**1**	**R.KMFEDLLEYR.N**
**86 - 95**	**1359.6750**	**1358.6677**	**1358.6540**	**10**	**1**	**R.KMFEDLLEYR.N** Oxidation (M)
**87 - 95**	**1215.5860**	**1214.5787**	**1214.5641**	**12**	**0**	**K.MFEDLLEYR.N**
**87 - 95**	**1231.5830**	**1230.5757**	**1230.5591**	**14**	**0**	**K.MFEDLLEYR.N** Oxidation (M)
**104 - 116**	**1598.7390**	**1597.7317**	**1597.7123**	**12**	**0**	**R.CFYTYDAFIEAAK.A**
**133 - 165**	**3663.6530**	**3662.6457**	**3662.5950**	**14**	**0**	**R.EIAAFFAQTGHETTGGWPDAPGGEYAWGYCFNR.E**
**166 - 182**	**1960.8080**	**1959.8007**	**1959.7727**	**14**	**0**	**R.EVGAASSDYCDPNYPCR.G**
**189 - 201**	**1623.8500**	**1622.8427**	**1622.8205**	**14**	**0**	**R.GPIQLSWNYNYLR.C**
**202 - 228**	**2871.4860**	**2870.4787**	**2870.4372**	**14**	**0**	**R.CGEGLGLGEELLNNPDLLATDPVLSFK.S**
**264 - 289**	**2723.3860**	**2722.3787**	**2722.3385**	**15**	**0**	**R.LPGYGLTTNIINGGIECGYVGNDAVR.N**
**292 - 301**	**1177.5790**	**1176.5717**	**1176.5638**	**7**	**0**	**R.IGFFTTFCGK.F**

126.
Match to: **clementine0.9_019310m|PAC:19259529** Score: **135** Expect: **4.3e-009**
Nominal mass (M_r): **28499**; Calculated pI value: **5.51**
NCBI BLAST search of clementine0.9_019310m|PAC:19259529 against nr
Unformatted sequence string for pasting into other applications

Fixed modifications: Carbamidomethyl (C)

Variable modifications: Oxidation (M)
Cleavage by Trypsin: cuts C-term side of KR unless next residue is P
Number of mass values matched: **13**
Sequence Coverage: **56%**

Matched peptides shown in **Bold Red**

```
  1 MANDAYEDAI AGLTKLLSEK SDLEGVAAAK IKQITADLEA AGSRDIDPVE
 51 RMKTGFIQFK TEKYEKNPDL YGALAKGQSP KFLVFACSDS RVCPSHILNF
101 QPGEAFMVRN IANMVPPYDQ KKYSGAGAAI EYAVLHLKVE NIVVIGHSCC
151 GGIKGLMSIP DNGTTASDFI EEWVKICSSA KSKVKKECND LSFEEQCKNC
201 EKEAVNVSLG NLLTYPFVRE SVVKNTLALK GAHYDFVNGK FELWDLDFNI
251 LPSVSV
```

Start - End	Observed	Mr(expt)	Mr(calc)	ppm	Miss	Sequence
33 - 44	1231.6370	1230.6297	1230.6204	8	0	K.QITADLEAAGSR.D
82 - 91	1201.5830	1200.5757	1200.5598	13	0	K.FLVFACSDSR.V
92 - 109	2102.0620	2101.0547	2101.0237	15	0	R.VCPSHILNFQPGEAFMVR.N
92 - 109	2118.0610	2117.0537	2117.0187	17	0	R.VCPSHILNFQPGEAFMVR.N Oxidation (M)
110 - 121	1389.6980	1388.6907	1388.6758	11	0	R.NIANMVPPYDQK.K
110 - 121	1405.6940	1404.6867	1404.6707	11	0	R.NIANMVPPYDQK.K Oxidation (M)
122 - 138	1790.9800	1789.9727	1789.9726	0	1	K.KYSGAGAAIEYAVLHLK.V
123 - 138	1662.9080	1661.9007	1661.8777	14	0	K.YSGAGAAIEYAVLHLK.V
139 - 154	1741.8950	1740.8877	1740.8651	13	0	K.VENIVVIGHSCCGGIK.G
155 - 175	2326.1340	2325.1267	2325.0835	19	0	K.GLMSIPDNGTTASDFIEEWVK.I Oxidation (M)
187 - 198	1558.6540	1557.6467	1557.6075	25	0	K.ECNDLSFEEQCK.N
203 - 219	1892.0570	1891.0497	1891.0203	16	0	K.EAVNVSLGNLLTYPFVR.E
231 - 240	1107.5310	1106.5237	1106.5145	8	0	K.GAHYDFVNGK.F

128.
Match to: **clementine0.9_019617m|PAC:19281824** Score: **162** Expect: **8.5e-012**
Nominal mass (M_r): **27724**; Calculated pI value: **5.55**
NCBI BLAST search of clementine0.9_019617m|PAC:19281824 against nr
Unformatted sequence string for pasting into other applications

Fixed modifications: Carbamidomethyl (C)
Variable modifications: Oxidation (M)
Cleavage by Trypsin: cuts C-term side of KR unless next residue is P
Number of mass values matched: **15**
Sequence Coverage: **74%**

Matched peptides shown in **Bold Red**

```
  1 MTKNYPTVSE DYKKAVEKCK RKLRGFIAEK NCAPLMLRIA WHSAGTYDVK
 51 TKTGGPFGTM RLAAEQAHSA NNGLDIAVRL LEPFKEQFPT ISYADLYQLA
```

101 **GVVGVEVTGG PDIPFHPGRD DKAEPPQEGR** LPDAKQGNDH LR**QVFGAQMG**
151 **LSDKDIVALS GGHTLGR**CHK ER**SGFEGPWT RNPLIFDNSY FTELLTGEKD**
201 **GLLQLPSDKA LLDDPVFRPL VEKYAADEDA FFADYAEAHL K**LSELGFAEA
251

Start - End	Observed	Mr(expt)	Mr(calc)	ppm	Miss	Sequence
31 - 38	974.4980	973.4907	973.4837	7	0	K.NCAPLMLR.I
39 - 50	1347.6850	1346.6777	1346.6619	12	0	R.IAWHSAGTYDVK.T
53 - 61	923.4490	922.4417	922.4331	9	0	K.TGGPFGTMR.L
62 - 79	1849.9750	1848.9677	1848.9442	13	0	R.LAAEQAHSANNGLDIAVR.L
86 - 119	3629.8760	3628.8687	3628.8202	13	0	K.EQFPTISYADLYQLAGVVGVEVTGGPDIPFHPGR.D
120 - 130	1241.5830	1240.5757	1240.5684	6	1	R.DDKAEPPQEGR.L
123 - 130	883.4530	882.4457	882.4195	30	0	K.AEPPQEGR.L
143 - 167	2557.3520	2556.3447	2556.3119	13	1	R.QVFGAQMGLSDKDIVALSGGHTLGR.C
143 - 167	2573.3530	2572.3457	2572.3068	15	1	R.QVFGAQMGLSDKDIVALSGGHTLGR.C Oxidation (M)
155 - 167	1295.7140	1294.7067	1294.6994	6	0	K.DIVALSGGHTLGR.C
173 - 181	1036.4920	1035.4847	1035.4774	7	0	R.SGFEGPWTR.N
182 - 199	2101.0750	2100.0677	2100.0415	12	0	R.NPLIFDNSYFTELLTGEK.D
182 - 209	3167.6340	3166.6267	3166.6074	6	1	R.NPLIFDNSYFTELLTGEKDGLLQLPSDK.A
210 - 223	1611.9340	1610.9267	1610.9032	15	0	K.ALLDDPVFRPLVEK.Y
224 - 241	2046.9430	2045.9357	2045.9007	17	0	K.YAADEDAFFADYAEAHLK.L

130.
Match to: **orange1.1g017504m|PAC:18102820** Score: **67** Expect: **0.025**
Nominal mass (M_r): **39870**; Calculated pI value: **5.95**
NCBI BLAST search of orange1.1g017504m|PAC:18102820 against nr
Unformatted sequence string for pasting into other applications

Fixed modifications: Carbamidomethyl (C)
Variable modifications: Oxidation (M)
Cleavage by Trypsin: cuts C-term side of KR unless next residue is P
Number of mass values matched: **11**
Sequence Coverage: **29%**

Matched peptides shown in **Bold Red**

1 MWGIIRQKVA AGGGSPVARI RPVVSNLRNY SSAVKQMMVR EALNSALDEE
51 MSADPK**VFLM GEEVGEYQGA YK**ISKGLLEK YGPERVLDTP ITEAGFTGIG
101 VGAAYYGLKP VVEFMTFNFS MQAIDHIINS AAK**SNYMSSG QISVPIVFRG**
151 **PNGAAAGVGA QHSHCYAAWY ASVPGLKVLS PYSSEDAR**GL LKAAIRDPDP
201 VVFLENELLY GESFPVSAEV LDSSFCLPIG KAKIEREGKD VTITAFSKIV
251 GLSLKAAEIL AK**EGISAEVI NLR**SIRPLDR STINASVRKT NRLVTVEEGF
301 PQHGVGAEIC ASVIEESFGY LDAPVER**IAG ADVPMPYAAN LERMAVPQVE**
351 **DIVR**AAKRAC YRSVPMAAAA

Start - End	Observed	Mr(expt)	Mr(calc)	ppm	Miss	Sequence

57 - 72	1819.8950	1818.8877	1818.8498	21	0	K.VFLMGEEVGEYQGAYK.I	
57 - 72	1835.8860	1834.8787	1834.8447	19	0	K.VFLMGEEVGEYQGAYK.I	Oxidation (M)
134 - 149	1784.9150	1783.9077	1783.8927	8	0	K.SNYMSSGQISVPIVFR.G	
134 - 149	1800.9180	1799.9107	1799.8876	13	0	K.SNYMSSGQISVPIVFR.G	Oxidation (M)
150 - 177	2797.3640	2796.3567	2796.3191	13	0	R.GPNGAAAGVGAQHSHCYAAWYASVPGLK.V	
178 - 188	1223.6010	1222.5937	1222.5830	9	0	K.VLSPYSSEDAR.G	
263 - 273	1200.6670	1199.6597	1199.6510	7	0	K.EGISAEVINLR.S	
328 - 343	1687.8640	1686.8567	1686.8399	10	0	R.IAGADVPMPYAANLER.M	
328 - 343	1703.8610	1702.8537	1702.8348	11	0	R.IAGADVPMPYAANLER.M	Oxidation (M)
344 - 354	1256.6750	1255.6677	1255.6595	7	0	R.MAVPQVEDIVR.A	
344 - 354	1272.6720	1271.6647	1271.6544	8	0	R.MAVPQVEDIVR.A	Oxidation (M)

132.
Match to: **orange1.1g016265m|PAC:18101604** Score: **141** Expect: **1.1e-009**
Nominal mass (M_r): **41894**; Calculated pI value: **8.77**
NCBI BLAST search of orange1.1g016265m|PAC:18101604 against nr
Unformatted sequence string for pasting into other applications

Fixed modifications: Carbamidomethyl (C)
Variable modifications: Oxidation (M)
Cleavage by Trypsin: cuts C-term side of KR unless next residue is P
Number of mass values matched: **11**
Sequence Coverage: **42%**

Matched peptides shown in **Bold Red**

```
  1 METILTTTAS KLTIFHSISS TKLNSFSRKF SVGFSGNKNR KKVTTLKQGS
 51 FSYLPLGVQA SASQAAASST EAEPTKVGTV PSEMKAWLYG EYGGVDVLKF
101 DEKVTVPQVK EDQVLIKVVA AALNPVDGKR RQGKFKATDS PLPTVPGYDV
151 AGVVVKVGTQ VKEFKEGDEV YGDINEKALE GPKQFGSLAE YTAVEERLLA
201 PKPKNLDFVQ AAGLPLAIET AYEGLERTGF SAGKSILVLN GSGGVGSLVI
251 QLAKQVFGAS RVAATSSTRN LEFLKSLGAD LAIDYTKDNF EDLPEKFDVV
301 YDAIGQCDRA VKAIKEGGTV VALTGAVTPP GFRFVVTSNG EVLKKLNPYL
351 ESGKVKPIID PKGPFPFSQV VEAFSYIETN KATGKVVIHP IP
```

Start - End	Observed	Mr(expt)	Mr(calc)	ppm	Miss	Sequence
86 - 99	1569.8100	1568.8027	1568.7875	10	0	K.AWLYGEYGGVDVLK.F
137 - 156	1985.0820	1984.0747	1984.0517	12	0	K.ATDSPLPTVPGYDVAGVVVK.V
163 - 177	1771.8680	1770.8607	1770.7948	37	1	K.EFKEGDEVYGDINEK.A
166 - 177	1367.6540	1366.6467	1366.5888	42	0	K.EGDEVYGDINEK.A
184 - 197	1599.7900	1598.7827	1598.7576	16	0	K.QFGSLAEYTAVEER.L
205 - 227	2490.3310	2489.3237	2489.2802	17	0	K.NLDFVQAAGLPLAIETAYEGLER.T
235 - 254	1925.1580	1924.1507	1924.1357	8	0	K.SILVLNGSGGVGSLVIQLAK.Q
288 - 309	2645.2270	2644.2197	2644.1752	17	1	K.DNFEDLPEKFDVVYDAIGQCDR.A
297 - 309	1557.7300	1556.7227	1556.6930	19	0	K.FDVVYDAIGQCDR.A
316 - 333	1728.9540	1727.9467	1727.9207	15	0	K.EGGTVVALTGAVTPPGFR.F

363 - 381	2160.0990	2159.0917	2159.0575	16	0	K.GPFPFSQVVEAFSYIETNK.A

134.
Match to: **orange1.1g008638m|PAC:18095844** Score: **278** Expect: **2.1e-023**
Nominal mass (M_r): **59862**; Calculated pI value: **6.06**
NCBI BLAST search of orange1.1g008638m|PAC:18095844 against nr
Unformatted sequence string for pasting into other applications

Fixed modifications: Carbamidomethyl (C)
Variable modifications: Oxidation (M)
Cleavage by Trypsin: cuts C-term side of KR unless next residue is P
Number of mass values matched: **27**
Sequence Coverage: **63%**

Matched peptides shown in **Bold Red**

```
  1 MASRRLLSSL LRSSVRRSPS KSSLSNSSVF SPAATRRASP YGHLLSRVSE
 51 YATSAAATAP PAQTPKSDVK KGGGGKITDE FTGKGAIGQV CQVIGAVVDV
101 RFDEGLPPIL TALEVVDHSV RLVLEVAQHM GEGVVRTIAM DGTEGLVRGQ
151 RVLNTGSPIT VPVGRVTLGR IMNVIGEPID EKGDLKTEHY LPIHREAPAF
201 VEQATEQQIL VTGIKVVDLL APYQRGGKIG LFGGAGVGKT VLIMELINNV
251 AKAHGGFSVF AGVGERTREG NDLYREMIES GVIKLGDKQA DSKCALVYGQ
301 MNEPPGARAR VGLTGLTVAE HFRDAEGQDV LLFIDNIFRF TQANSEVSAL
351 LGRIPSAVGY QPTLATDLGG LQERITTTKK GSITSVQAIY VPADDLTDPA
401 PATTFAHLDA TTVLSRQISE LGIYPAVDPL DSTSRMLSPH ILGEEHYNTA
451 RGVQKVLQNY KNLQDIIAIL GMDELSEDDK LTVARARKIQ RFLSQPFHVA
501 EVFTGAPGKY VELKESIASF QGVLDGKYDD LPEQSFYMVG GIEEVIAKAE
551 KIAKESAA
```

Start - End	Observed	Mr(expt)	Mr(calc)	ppm	Miss	Sequence
85 - 101	1740.9700	1739.9627	1739.9353	16	0	K.GAIGQVCQVIGAVVDVR.F
102 - 121	2207.2090	2206.2017	2206.1634	17	0	R.FDEGLPPILTALEVVDHSVR.L
122 - 136	1636.9060	1635.8987	1635.8767	13	0	R.LVLEVAQHMGEGVVR.T
122 - 136	1652.9020	1651.8947	1651.8716	14	0	R.LVLEVAQHMGEGVVR.T Oxidation (M)
137 - 148	1262.6660	1261.6587	1261.6336	20	0	R.TIAMDGTEGLVR.G
137 - 148	1278.6560	1277.6487	1277.6286	16	0	R.TIAMDGTEGLVR.G Oxidation (M)
152 - 165	1409.8300	1408.8227	1408.8038	13	0	R.VLNTGSPITVPVGR.V
171 - 182	1373.7000	1372.6927	1372.6908	1	0	R.IMNVIGEPIDEK.G Oxidation (M)
187 - 195	1165.6230	1164.6157	1164.6040	10	0	K.TEHYLPIHR.E
196 - 215	2172.1870	2171.1797	2171.1474	15	0	R.EAPAFVEQATEQQILVTGIK.V
216 - 225	1173.6770	1172.6697	1172.6554	12	0	K.VVDLLAPYQR.G
240 - 252	1457.8540	1456.8467	1456.8323	10	0	K.TVLIMELINNVAK.A
253 - 266	1390.7070	1389.6997	1389.6790	15	0	K.AHGGFSVFAGVGER.T
294 - 308	1662.7910	1661.7837	1661.7654	11	0	K.CALVYGQMNEPPGAR.A
294 - 308	1678.7950	1677.7877	1677.7603	16	0	K.CALVYGQMNEPPGAR.A Oxidation (M)
311 - 323	1399.7910	1398.7837	1398.7620	16	0	R.VGLTGLTVAEHFR.D

324 - 339	1864.9760	1863.9687	1863.9367	17	0	R.DAEGQDVLLFIDNIFR.F
340 - 353	1492.7970	1491.7897	1491.7681	14	0	R.FTQANSEVSALLGR.I
354 - 374	2186.1840	2185.1767	2185.1379	18	0	R.IPSAVGYQPTLATDLGGLQER.I
380 - 416	3843.0400	3842.0327	3841.9738	15	1	K.KGSITSVQAIYVPADDLTDPAPATTFAHLDATTVLSR.Q
381 - 416	3714.9450	3713.9377	3713.8789	16	0	K.GSITSVQAIYVPADDLTDPAPATTFAHLDATTVLSR.Q
417 - 435	2061.0830	2060.0757	2060.0426	16	0	R.QISELGIYPAVDPLDSTSR.M
436 - 451	1883.9390	1882.9317	1882.8996	17	0	R.MLSPHILGEEHYNTAR.G Oxidation (M)
462 - 485	2672.4250	2671.4177	2671.3738	16	1	K.NLQDIIAILGMDELSEDDKLTVAR.A
462 - 485	2688.4220	2687.4147	2687.3687	17	1	K.NLQDIIAILGMDELSEDDKLTVAR.A Oxidation (M)
528 - 548	2403.1910	2402.1837	2402.1352	20	0	K.YDDLPEQSFYMVGGIEEVIAK.A
528 - 548	2419.1840	2418.1767	2418.1301	19	0	K.YDDLPEQSFYMVGGIEEVIAK.A Oxidation (M)

136.
Match to: **clementine0.9_032803m|PAC:19261734** Score: **69** Expect: **0.015**
Nominal mass (M_r): **44701**; Calculated pI value: **5.49**
NCBI BLAST search of clementine0.9_032803m|PAC:19261734 against nr
Unformatted sequence string for pasting into other applications

Fixed modifications: Carbamidomethyl (C)
Variable modifications: Oxidation (M)
Cleavage by Trypsin: cuts C-term side of KR unless next residue is P
Number of mass values matched: **9**
Sequence Coverage: **30%**

Matched peptides shown in **Bold Red**

```
  1 MAASAMVLDP KPVSEPPPST RSDLTEQWSD EDDLYSRLKS LQRQLEFIDI
 51 QEEYVKDEQK NLKRELLRAQ EEVKRIQSVP LVIGQFMEMV DQNNGIVGST
101 TGSNYYVRIL STINRELLKP SASVALHRHS NALVDVLPPE ADSSISLLSQ
151 SEKPDVTYND IGGCDIQKQE IREAVELPLT HHELYKQIGI DPPRGVLLYG
201 PPGTGKTMLA KAVANHTTAA FIRVVGSEFV QKYLGEGPRM VRDVDAIATA
251 RFDAQTGADR EVQRILMELL NQMDGFDQTV NVKVIMATNR ADTLDPALLR
301 PGRLDRKIEF PLPDRRQKRL VFQVCTAKMN LSDEVDLEDY VSRPDKISAA
351 EIAAICQEAG MHAVRKNRYV ILPKDFEKGY RTNVKKPDTD FEFYK
```

Start - End	Observed	Mr(expt)	Mr(calc)	ppm	Miss	Sequence
22 - 37	1958.8640	1957.8567	1957.8177	20	0	R.SDLTEQWSDEDDLYSR.L
44 - 60	2154.0930	2153.0857	2153.0528	15	1	R.QLEFIDIQEEYVKDEQK.N
116 - 128	1420.8120	1419.8047	1419.8198	-11	0	R.ELLKPSASVALHR.H
173 - 186	1678.8890	1677.8817	1677.8726	5	0	R.EAVELPLTHHELYK.Q
291 - 303	1394.7860	1393.7787	1393.7677	8	0	R.ADTLDPALLRPGR.L
307 - 315	1114.6280	1113.6207	1113.6182	2	1	R.KIEFPLPDR.R
329 - 346	2141.0330	2140.0257	2139.9630	29	0	K.MNLSDEVDLEDYVSRPDK.I Oxidation (M)
347 - 365	1998.0080	1997.0007	1996.9822	9	0	K.ISAAEIAAICQEAGMHAVR.K
347 - 365	2014.0140	2013.0067	2012.9771	15	0	K.ISAAEIAAICQEAGMHAVR.K Oxidation (M)

138.
Match to: **orange1.1g012445m|PAC:18104933** Score: **163** Expect: **6.7e-012**
Nominal mass (M_r): **50379**; Calculated pI value: **5.91**
NCBI BLAST search of orange1.1g012445m|PAC:18104933 against nr
Unformatted sequence string for pasting into other applications

Fixed modifications: Carbamidomethyl (C)
Variable modifications: Oxidation (M)
Cleavage by Trypsin: cuts C-term side of KR unless next residue is P
Number of mass values matched: **20**
Sequence Coverage: **58%**

Matched peptides shown in **Bold Red**

```
  1 MYRNAASRLR ALKGHVRCRV PSATRFASSS AVASTSSSSG GLFSWLTGER
 51 SSSSPSLDFP LPGVSLPPSL PDYVEPGKTK ISTLPNGVKI ASETSVSPVA
101 SISLYVGCGS IYESPISFGT THLLERMAFR STRNRSHLRI VREVEAIGGN
151 VQASASREQM GYSFDALKTY VPEMVELLID CVRNPVFLDW EVNEQLTKVK
201 SEISEVSNNP QSLLLEAIHS AGYSGALANP LLAPESAINR LNSTLLEEFV
251 AENYTGPRMV LAASGVEHDQ LVSVAEPLLS DLPSIHPREE PKSVYTGGDY
301 RCQADSGDQL THFVLAFELP GGWHKDKDAM TLTVLQMLLG GGGSFSAGGP
351 GKGMYSRLYR RVLNEFPQVQ SFSAFSNIYN HSGMFGIQGT TGSDFVSKAI
401 DLAARELISV ATPGEVDQVQ LDRAKQSTKS AILMNLESRM VVSEDIGRQV
451 LTYGERYGWR PDI
```

Start - End	Observed	Mr(expt)	Mr(calc)	ppm	Miss	Sequence
51 - 78	2869.4920	2868.4847	2868.4433	14	0	R.SSSSPSLDFPLPGVSLPPSLPDYVEPGK.T
90 - 126	3928.0230	3927.0157	3926.9612	14	0	K.IASETSVSPVASISLYVGCGSIYESPISFGTTHLLER.M
143 - 157	1487.7620	1486.7547	1486.7376	12	0	R.EVEAIGGNVQASASR.E
158 - 168	1288.6040	1287.5967	1287.5805	13	0	R.EQMGYSFDALK.T
158 - 168	1304.6090	1303.6017	1303.5754	20	0	R.EQMGYSFDALK.T Oxidation (M)
169 - 183	1836.9520	1835.9447	1835.9161	16	0	K.TYVPEMVELLIDCVR.N
184 - 198	1831.9470	1830.9397	1830.9152	13	0	R.NPVFLDWEVNEQLTK.V
241 - 258	2053.0530	2052.0457	2052.0164	14	0	R.LNSTLLEEFVAENYTGPR.M
259 - 288	3180.7140	3179.7067	3179.6649	13	0	R.MVLAASGVEHDQLVSVAEPLLSDLPSIHPR.E
259 - 288	3196.7150	3195.7077	3195.6598	15	0	R.MVLAASGVEHDQLVSVAEPLLSDLPSIHPR.E Oxidation (M)
293 - 301	1017.4820	1016.4747	1016.4563	18	0	K.SVYTGGDYR.C
302 - 325	2713.3350	2712.3277	2712.2755	19	0	R.CQADSGDQLTHFVLAFELPGGWHK.D
326 - 352	2608.3530	2607.3457	2607.3037	16	1	K.DKDAMTLTVLQMLLGGGGSFSAGGPGK.G
326 - 352	2624.3470	2623.3397	2623.2986	16	1	K.DKDAMTLTVLQMLLGGGGSFSAGGPGK.G Oxidation (M)
326 - 352	2640.3380	2639.3307	2639.2935	14	1	K.DKDAMTLTVLQMLLGGGGSFSAGGPGK.G 2 Oxidation (M)
406 - 423	1969.0500	1968.0427	1968.0164	13	0	R.ELISVATPGEVDQVQLDR.A
430 - 439	1133.6080	1132.6007	1132.5910	9	0	K.SAILMNLESR.M
430 - 439	1149.5980	1148.5907	1148.5859	4	0	K.SAILMNLESR.M Oxidation (M)
449 - 456	965.5140	964.5067	964.4978	9	0	R.QVLTYGER.Y
449 - 463	1852.9440	1851.9367	1851.9268	5	1	R.QVLTYGERYGWRPDI.-

139.
Match to: **orange1.1g043137m|PAC:18101066** Score: **142** Expect: **8.5e-010**
Nominal mass (M_r): **48059**; Calculated pI value: **5.54**
NCBI BLAST search of orange1.1g043137m|PAC:18101066 against nr
Unformatted sequence string for pasting into other applications

Fixed modifications: Carbamidomethyl (C)
Variable modifications: Oxidation (M)
Cleavage by Trypsin: cuts C-term side of KR unless next residue is P
Number of mass values matched: **15**
Sequence Coverage: **53%**

Matched peptides shown in **Bold Red**

```
  1 MAITITAVKA RQIFDSRGNP TVEVDVTTSD GHVARAAVPS GASTGIYEAL
 51 ELRDGGSDYL GKGVSKAVSN VNAIIGPALA GKDPTEQTAI DNYMVQQLDG
101 TVNEWGWCKQ KLGANAILAV SLAVCKAGAH VKKIPLYKHI AELSGNKNLV
151 LPVPAFNVIN GGSHAGNKLA MQEFMILPVG ASCFKEAMKM GVEVYHHLKA
201 VIKKKYGQDA TNVGDEGGFA PNIQENKEGL ELLNTAIAKA GYTGKVVIGM
251 DVAASEFYGS DKTYDLNFKE ENNDGSQKIS GDALKDLYKS FISDYPIVSI
301 EDPFDQDDWE HYAKLTSEVG EKVQIVGDDL LVTNPKRVEK AIKEKTCNAL
351 LLKVNQIGSV TESIEAVRMS KQAGWGVMAS HRSGETEDTF IADLSVGLAT
401 GQIKTGAPCR SERLAKYNQL LRIEEELGAE AVYAGAKFRA PVEPY
```

Start - End	Observed	Mr(expt)	Mr(calc)	ppm	Miss	Sequence
18 - 35	1853.9250	1852.9177	1852.8916	14	0	R.GNPTVEVDVTTSDGHVAR.A
36 - 53	1804.9690	1803.9617	1803.9366	14	0	R.AAVPSGASTGIYEALELR.D
112 - 126	1499.8830	1498.8757	1498.8541	14	0	K.LGANAILAVSLAVCK.A
148 - 168	2118.1650	2117.1577	2117.1382	9	0	K.NLVLPVPAFNVINGGSHAGNK.L
169 - 185	1973.9930	1972.9857	1972.9461	20	0	K.LAMQEFMILPVGASCFK.E 2 Oxidation (M)
206 - 227	2324.0790	2323.0717	2323.0353	16	0	K.YGQDATNVGDEGGFAPNIQENK.E
246 - 262	1787.8970	1786.8897	1786.8448	25	0	K.VVIGMDVAASEFYGSDK.T
290 - 314	3016.4040	3015.3967	3015.3450	17	0	K.SFISDYPIVSIEDPFDQDDWEHYAK.L
323 - 336	1510.8520	1509.8447	1509.8403	3	0	K.VQIVGDDLLVTNPK.R
354 - 368	1601.8690	1600.8617	1600.8420	12	0	K.VNQIGSVTESIEAVR.M
372 - 382	1199.5800	1198.5727	1198.5666	5	0	K.QAGWGVMASHR.S
372 - 382	1215.5850	1214.5777	1214.5615	13	0	K.QAGWGVMASHR.S Oxidation (M)
383 - 404	2252.1610	2251.1537	2251.1220	14	0	R.SGETEDTFIADLSVGLATGQIK.T
423 - 437	1549.7700	1548.7627	1548.7671	-3	0	R.IEEELGAEAVYAGAK.F
438 - 445	978.5090	977.5017	977.4971	5	1	K.FRAPVEPY.-

140.
Match to: **clementine0.9_007135m|PAC:19257184** Score: **79** Expect: **0.0016**
Nominal mass (M_r): **56809**; Calculated pI value: **5.19**
NCBI BLAST search of clementine0.9_007135m|PAC:19257184 against nr
Unformatted sequence string for pasting into other applications

Fixed modifications: Carbamidomethyl (C)
Variable modifications: Oxidation (M)
Cleavage by Trypsin: cuts C-term side of KR unless next residue is P
Number of mass values matched: **12**
Sequence Coverage: **32%**

Matched peptides shown in **Bold Red**

```
  1 MLKGVEELAD AVKVTMGPKG RNVVIEQSWG APKVTKDGVT VAKSIEFKDK
 51 VKNIGASLVK QVANATNDVA GDGTTCATVL TRAIFTEGCK SVAAGMNAMD
101 LRRGITMAVD AVVTNLKSRA RMISTSEEIA QVGTISANGE REIGELIAKA
151 MEKVGKEGVI TIQDGKTLYN ELEVVEGMKL DRGYISPYFI TNQKNQKCEL
201 EDPLILVHEK KISNLTAVVR VLELALKRQR PLLIVAEDVE SEALATLILN
251 KLRAGIKVCA IKAPGFGENR KANMQDLAVL TGGDLITEEL GMDLEKVNLD
301 MLGTCKKVTI SKDDTVILDG AGDKKSIEER CEQIRSAIEN STSDYDKEKL
351 QERLAKLSGG VAVLKIGGAS EAEVGEKKDR VTDALNATKA AVEEGIVPGG
401 GVALLYAAKE LEKLSTANFD QKIGVQIIQN ALKTPVHTIA ANAGVEGAVV
451 VGKLLEQDNT DLGYDAAKGE YVDMVKSGII DPLKVIRTAL VDAASVSSLM
501 TTTEAIVVEL PKEEKEAPGG MGGMGGMGGM DY
```

Start - End	Observed	Mr(expt)	Mr(calc)	ppm	Miss	Sequence	
22 - 33	1327.7160	1326.7087	1326.6932	12	0	R.NVVIEQSWGAPK.V	
122 - 141	2093.0420	2092.0347	2092.0106	12	0	R.MISTSEEIAQVGTISANGER.E	
122 - 141	2109.0380	2108.0307	2108.0055	12	0	R.MISTSEEIAQVGTISANGER.E	Oxidation (M)
167 - 179	1524.7910	1523.7837	1523.7541	19	0	K.TLYNELEVVEGMK.L	
167 - 179	1540.7670	1539.7597	1539.7490	7	0	K.TLYNELEVVEGMK.L	Oxidation (M)
183 - 194	1430.7530	1429.7457	1429.7242	15	0	R.GYISPYFITNQK.N	
198 - 210	1594.8310	1593.8237	1593.8072	10	0	K.CELEDPLILVHEK.K	
211 - 220	1100.6780	1099.6707	1099.6713	-1	1	K.KISNLTAVVR.V	
229 - 251	2535.4770	2534.4697	2534.4319	15	0	R.QRPLLIVAEDVESEALATLILNK.L	
390 - 409	1885.0720	1884.0647	1884.0356	15	0	K.AAVEEGIVPGGGVALLYAAK.E	
434 - 453	1890.0600	1889.0527	1889.0371	8	0	K.TPVHTIAANAGVEGAVVVGK.L	
488 - 515	2948.5640	2947.5567	2947.5311	9	1	R.TALVDAASVSSLMTTTEAIVVELPKEEK.E	Oxidation (M)

144.
Match to: **orange1.1g047192m|PAC:18112249** Score: **78** Expect: **0.0021**
Nominal mass (M_r): **66158**; Calculated pI value: **8.50**
NCBI BLAST search of orange1.1g047192m|PAC:18112249 against nr
Unformatted sequence string for pasting into other applications

Fixed modifications: Carbamidomethyl (C)
Variable modifications: Oxidation (M)
Cleavage by Trypsin: cuts C-term side of KR unless next residue is P
Number of mass values matched: **7**
Sequence Coverage: **20%**

Matched peptides shown in **Bold Red**

```
  1 MESCCSTCTK LSSASPSHPC IADSRGFGRK FTRNSLDHHL LSSSLPKPFL
 51 QVNNARNTFL YRRSSSRFPS TASRGIISAE AWDFGRFLKT LYFFNGPPSP
101 AKFVEFLVEK LSGPSPKEPV KAMETSGIVL VAGATGGVGR RVVDILRNKG
151 LPVRVLVRNE EKARKMLGPD VDLIVGDITK ENTLTPEYFK GVRKVINAVS
201 VIVGPKEGDT PDRAKYSQGI KFFEPEIKGD SPEMVEYLGM RNLINAVKGS
251 VGLQNGKLLF GFEENSLKEL PWGALDDVVM GGVSESTFQI DRTGGENGAP
301 TGLFKGVVST ANNGGFTSIR TRNFAEPEDL SAYDGLKLRL KGDGRRYKFV
351 VRTSSDWDTV GYTASFDTVG GQWQSIRLPF SSLRPIFQAR TVLDAPPFDP
401 SNIVSLQLMF SKFEYDGKLN PTFVEGAFQL PVSSIQSYIK DPVTPRFVHV
451 SSAGVTRPER PGLDLSKQPP AVRLNKELGF ILTFKLKGED LIRESGIPYT
501 IVRPCALTEE PAGADLIFDQ GDNITGKISR EEVARICVAA LESPFALDKT
551 FEVKSTIPFS ESFTVDPENP PQEKDYNIYF KGLKDGITGK ESLEQSPVPV
601
```

Start - End	Observed	Mr(expt)	Mr(calc)	ppm	Miss	Sequence	
90 - 102	1438.7470	1437.7397	1437.7292	7	0	K.TLYFFNGPPSPAK.F	
258 - 268	1296.6920	1295.6847	1295.6761	7	0	K.LLFGFEENSLK.E	
269 - 292	2637.2880	2636.2807	2636.2429	14	0	K.ELPWGALDDVVMGGVSESTFQIDR.T	Oxidation (M)
306 - 320	1479.7730	1478.7657	1478.7478	12	0	K.GVVSTANNGGFTSIR.T	
353 - 377	2764.3220	2763.3147	2763.2413	27	0	R.TSSDWDTVGYTASFDTVGGQWQSIR.L	
447 - 467	2252.2400	2251.2327	2251.2073	11	0	R.FVHVSSAGVTRPERPGLDLSK.Q	
536 - 549	1533.8130	1532.8057	1532.7908	10	0	R.ICVAALESPFALDK.T	

149.
Match to: **clementine0.9_024678m|PAC:19282974** Score: **80** Expect: **0.0012**
Nominal mass (M_r): **16349**; Calculated pI value: **5.93**
NCBI BLAST search of clementine0.9_024678m|PAC:19282974 against nr
Unformatted sequence string for pasting into other applications

Fixed modifications: Carbamidomethyl (C)
Variable modifications: Oxidation (M)
Cleavage by Trypsin: cuts C-term side of KR unless next residue is P
Number of mass values matched: **7**
Sequence Coverage: **56%**

Matched peptides shown in **Bold Red**

1 MEQTFIMIKP DGVQR**GLVGE IIIR**FEKKGF SLKGLK**LMTV DRPFAEK**HYE
51 DLSSKPFFGS LIEYITSGPV VAMIWEGEGV VKTGR**TIIGA TNPAQSAPGT**
101 **IRGDLAIVTG RNIIHGSDSV ESAQKEIALW FPDGPINWQS SLHPWIYE**

Start - End	Observed	Mr(expt)	Mr(calc)	ppm	Miss	Sequence
16 - 24	969.6200	968.6127	968.6019	11	0	R.GLVGEIIIR.F
37 - 47	1306.6970	1305.6897	1305.6751	11	0	K.LMTVDRPFAEK.H
37 - 47	1322.6910	1321.6837	1321.6700	10	0	K.LMTVDRPFAEK.H Oxidation (M)
86 - 102	1667.9340	1666.9267	1666.9002	16	0	R.TIIGATNPAQSAPGTIR.G
103 - 111	901.5100	900.5027	900.5029	-0	0	R.GDLAIVTGR.N
112 - 125	1484.7520	1483.7447	1483.7267	12	0	R.NIIHGSDSVESAQK.E
126 - 148	2798.4100	2797.4027	2797.3540	17	0	K.EIALWFPDGPINWQSSLHPWIYE.-

152.
Match to: **clementine0.9_023949m|PAC:19285413** Score: **61** Expect: **0.11**
Nominal mass (M_r): **17821**; Calculated pI value: **5.93**
NCBI BLAST search of clementine0.9_023949m|PAC:19285413 against nr
Unformatted sequence string for pasting into other applications

Fixed modifications: Carbamidomethyl (C)
Variable modifications: Oxidation (M)
Cleavage by Trypsin: cuts C-term side of KR unless next residue is P
Number of mass values matched: **3**
Sequence Coverage: **35%**

Matched peptides shown in **Bold Red**

1 MNVEKLMKMA GAVRTGGKGS VRRKKKAVHK TTTTDDKRLQ STLKRIGVNA
51 IPAIEEVNIF KDDVVIQFLN PKVQASIAAN TWVVSGAPQT KK**LQDILPGI**
101 **INQLGPDNLD NLRK**LAEQFQ K**QAPNAGTGA PTTQEDDDDE VPELVAGETF**
151 **EAAAEEK**TEK PDAAS

Start - End	Observed	Mr(expt)	Mr(calc)	ppm	Miss	Sequence
93 - 113	2331.3070	2330.2997	2330.2594	17	0	K.LQDILPGIINQLGPDNLDNLR.K
93 - 114	2459.3990	2458.3917	2458.3543	15	1	K.LQDILPGIINQLGPDNLDNLRK.L
122 - 157	3702.7130	3701.7057	3701.6340	19	0	K.QAPNAGTGAPTTQEDDDDEVPELVAGETFEAAAEEK.T

153.
Match to: **orange1.1g031474m|PAC:18117825** Score: **105** Expect: **4.3e-006**
Nominal mass (M_r): **17806**; Calculated pI value: **6.74**
NCBI BLAST search of orange1.1g031474m|PAC:18117825 against nr
Unformatted sequence string for pasting into other applications

Fixed modifications: Carbamidomethyl (C)

Variable modifications: Oxidation (M)
Cleavage by Trypsin: cuts C-term side of KR unless next residue is P
Number of mass values matched: **7**
Sequence Coverage: **49%**

Matched peptides shown in **Bold Red**

```
  1 MSTPFVTAIS FLLLTFSTKP LVGLADPLVD VHGNKVEASR DYYLVSAIRG
 51 AGGGGLTLFR GRNELCPLDV VQLSSDSERG TRLRFSMHDK TSIINEDVDL
101 NVRFSTETRC NEPTVWRVDS YDPSRGKWFI TTGGVEGNPG AQTLKNWFKF
151 ERIGRDRGE
```

Start - End	Observed	Mr(expt)	Mr(calc)	ppm	Miss	Sequence
41 - 49	1099.5950	1098.5877	1098.5709	15	0	R.DYYLVSAIR.G
50 - 60	1005.5560	1004.5487	1004.5403	8	0	R.GAGGGGLTLFR.G
61 - 79	2174.0930	2173.0857	2173.0433	20	1	R.GRNELCPLDVVQLSSDSER.G
63 - 79	1960.9630	1959.9557	1959.9208	18	0	R.NELCPLDVVQLSSDSER.G
91 - 103	1487.7930	1486.7857	1486.7627	15	0	K.TSIINEDVDLNVR.F
110 - 117	1061.5030	1060.4957	1060.4760	19	0	R.CNEPTVWR.V
128 - 145	1875.9900	1874.9827	1874.9527	16	0	K.WFITTGGVEGNPGAQTLK.N

154.
Match to: **clementine0.9_018172m|PAC:19279067** Score: **74** Expect: **0.0059**
Nominal mass (M_r): **30236**; Calculated pI value: **7.10**
NCBI BLAST search of clementine0.9_018172m|PAC:19279067 against nr
Unformatted sequence string for pasting into other applications

Fixed modifications: Carbamidomethyl (C)
Variable modifications: Oxidation (M)
Cleavage by Trypsin: cuts C-term side of KR unless next residue is P
Number of mass values matched: **7**
Sequence Coverage: **37%**

Matched peptides shown in **Bold Red**

```
  1 MLAAASRILS RSTPLLKPSL ARTHFPAGYR FAVRSMADSA ASPFKKIQIQ
 51 RDDTTFDAYV VGKEDAPGIV VVQEWWGVDF EIKNHAVKIS QLNPGFKALI
101 PDLYRGKVGL DTAEAQHLMS GLDWPGAVKD IHASVNWLKA NGSKKVGVTG
151 YCMGGALAIA SSVLVPEVDA VVSFYGVPPP ELADPTQAKA PVQAHFGELD
201 NFVGFSDVKT AKALEEKLKA SGVPYEVHIY PGSAHAFMNI SPDGVKRRKE
251 MGMDDHDPAA VELAWSRFQS WMTRYLSA
```

Start - End	Observed	Mr(expt)	Mr(calc)	ppm	Miss	Sequence	
98 - 105	960.5570	959.5497	959.5440	6	0	K.ALIPDLYR.G	
108 - 129	2311.1350	2310.1277	2310.1314	-2	0	K.VGLDTAEAQHLMSGLDWPGAVK.D	Oxidation (M)
130 - 139	1182.6340	1181.6267	1181.6193	6	0	K.DIHASVNWLK.A	

190 - 209	2177.0700	2176.0627	2176.0589	2	0	K.APVQAHFGELDNFVGFSDVK.T	
220 - 246	2843.4060	2842.3987	2842.3748	8	0	K.ASGVPYEVHIYPGSAHAFMNISPDGVK.R	
220 - 246	2859.3610	2858.3537	2858.3698	-6	0	K.ASGVPYEVHIYPGSAHAFMNISPDGVK.R	Oxidation (M)
250 - 267	2061.8800	2060.8727	2060.8568	8	0	K.EMGMDDHDPAAVELAWSR.F	2 Oxidation (M)

159.
Match to: **clementine0.9_019405m|PAC:19255951** Score: **78** Expect: **0.0019**
Nominal mass (M_r): **28483**; Calculated pI value: **6.39**
NCBI BLAST search of clementine0.9_019405m|PAC:19255951 against nr
Unformatted sequence string for pasting into other applications

Fixed modifications: Carbamidomethyl (C)
Variable modifications: Oxidation (M)
Cleavage by Trypsin: cuts C-term side of KR unless next residue is P
Number of mass values matched: **8**
Sequence Coverage: **38%**

Matched peptides shown in **Bold Red**

1 MLKTEKEDLK PVAAAKVEQI TAQLQTPSDT KAFDSVER**IK EGFIHFK**REK
51 YEKNPALYSE LAKGQSPK**YM VFACSDSRVC PSHVLDFQPG EAFVVRNVAN**
101 **IVPPYDQTKY AGVGAAVEYA VLHLKVSNIV VIGHSACGGI K**GLMSFTFDG
151 NNSTDFIEDW VKIGIPAKSK VLTEHGDKPF GDQCTYCEK**E AVNVSLSNLL**
201 **TYPFVR**EGLV NKTLALKGGY YDFVNGSFEL WGLDFSLSPP LSVKDVATIL
251 HWKLH

Start - End	Observed	Mr(expt)	Mr(calc)	ppm	Miss	Sequence	
39 - 47	1118.6630	1117.6557	1117.6284	24	1	R.IKEGFIHFK.R	
69 - 78	1235.5380	1234.5307	1234.5111	16	0	K.YMVFACSDSR.V	
69 - 78	1251.5350	1250.5277	1250.5060	17	0	K.YMVFACSDSR.V	Oxidation (M)
79 - 96	2057.0730	2056.0657	2056.0201	22	0	R.VCPSHVLDFQPGEAFVVR.N	
97 - 109	1458.7740	1457.7667	1457.7514	10	0	R.NVANIVPPYDQTK.Y	
110 - 125	1660.9380	1659.9307	1659.8984	19	0	K.YAGVGAAVEYAVLHLK.V	
126 - 141	1610.8970	1609.8897	1609.8610	18	0	K.VSNIVVIGHSACGGIK.G	
190 - 206	1922.0780	1921.0707	1921.0309	21	0	K.EAVNVSLSNLLTYPFVR.E	

161.
Match to: **orange1.1g016566m|PAC:18107937** Score: **223** Expect: **6.7e-018**
Nominal mass (M_r): **42300**; Calculated pI value: **8.61**
NCBI BLAST search of orange1.1g016566m|PAC:18107937 against nr
Unformatted sequence string for pasting into other applications

Fixed modifications: Carbamidomethyl (C)
Variable modifications: Oxidation (M)
Cleavage by Trypsin: cuts C-term side of KR unless next residue is P

Number of mass values matched: **18**
Sequence Coverage: **50%**

Matched peptides shown in **Bold Red**

```
  1 MAARPASLTQ QQIRLQSKSQ SPMASSLSTA ASSRLLCSTT AAAAAAAKLS
 51 FSSASSLSFS LSSPSSLKCL RFSPLISQRR SSVNRGYSTV PTTKCAASDP
101 DQLKSAREDI RELLKSTFCH PILVRLGWHD AGTYDKNIEE WPRRGGANAS
151 LRFEVELKHA ANAGLVNALK LIQPIKDKYS GVTYADLFQL ASATAIEEAG
201 GPKIPMKYGR VDVSGPEQCP EEGRLPAAGP PSPAEHLRNV FYRMGLNDKE
251 IVALSGAHTV GRSRPERSGW GKPETKYTKD GPGAPGGQSW TVQWLKFDNS
301 YFKDIKERRD EDLLVLPTDA VLFEDPSFKV YAEKYAEDQE AFFKDYAEAH
351 AKLSNLGAKF DPPEGIVLDD GAAPEKFVAA KYSSGKD
```

Start - End	Observed	Mr(expt)	Mr(calc)	ppm	Miss	Sequence
2 - 14	1439.8180	1438.8107	1438.8004	7	0	M.AARPASLTQQQIR.L
19 - 34	1567.7760	1566.7687	1566.7308	24	0	K.SQSPMASSLSTAASSR.L
116 - 125	1229.6650	1228.6577	1228.6387	16	0	K.STFCHPILVR.L
126 - 136	1262.6110	1261.6037	1261.5728	25	0	R.LGWHDAGTYDK.N
137 - 143	943.4670	942.4597	942.4559	4	0	K.NIEEWPR.R
177 - 203	2802.4370	2801.4297	2801.3759	19	1	K.DKYSGVTYADLFQLASATAIEEAGGPK.I
179 - 203	2559.3110	2558.3037	2558.2540	19	0	K.YSGVTYADLFQLASATAIEEAGGPK.I
211 - 224	1558.7040	1557.6967	1557.6729	15	0	R.VDVSGPEQCPEEGR.L
211 - 238	2952.4780	2951.4707	2951.4196	17	1	R.VDVSGPEQCPEEGRLPAAGPPSPAEHLR.N
225 - 238	1412.7850	1411.7777	1411.7572	15	0	R.LPAAGPPSPAEHLR.N
244 - 262	1968.0660	1967.0587	1967.0258	17	1	R.MGLNDKEIVALSGAHTVGR.S
244 - 262	1984.0640	1983.0567	1983.0208	18	1	R.MGLNDKEIVALSGAHTVGR.S Oxidation (M)
250 - 262	1309.7350	1308.7277	1308.7150	10	0	K.EIVALSGAHTVGR.S
280 - 296	1783.9030	1782.8957	1782.8690	15	0	K.DGPGAPGGQSWTVQWLK.F
309 - 329	2419.2880	2418.2807	2418.2319	20	1	R.RDEDLLVLPTDAVLFEDPSFK.V
310 - 329	2263.1770	2262.1697	2262.1308	17	0	R.DEDLLVLPTDAVLFEDPSFK.V
335 - 344	1247.5840	1246.5767	1246.5506	21	0	K.YAEDQEAFFK.D
360 - 376	1769.8910	1768.8837	1768.8519	18	0	K.FDPPEGIVLDDGAAPEK.F

164.
Match to: **clementine0.9_011462m|PAC:19259581** Score: **147** Expect: **2.7e-010**
Nominal mass (M_r): **45094**; Calculated pI value: **8.46**
NCBI BLAST search of clementine0.9_011462m|PAC:19259581 against nr
Unformatted sequence string for pasting into other applications

Fixed modifications: Carbamidomethyl (C)
Variable modifications: Oxidation (M)
Cleavage by Trypsin: cuts C-term side of KR unless next residue is P
Number of mass values matched: **10**
Sequence Coverage: **38%**

Matched peptides shown in **Bold Red**

```
  1 MGSSLRPSEP IMLVKPSLSS LFAPRLSRAN AQAQQPRFIS TSRACSLPSS
 51 DSSSSSSSLS RRQFVSQTAT LSLSISLAAT TGLYEQPAKS EEALSAWERV
101 YIPVDPGVVL LDIAFVPDDL NHGFLLGTRQ TLLETKDGGK TWAPRSIPSA
151 EEEDFNYRFN SISFKGKEGW IVGKPAILLH TSDAGESWER IPLSSQLPGD
201 MVYIKATGEK SAEMVTDEGA IYITSNRGYN WRAAVQETVS ATLNRTVSSG
251 ISGASYYTGT FNTVNRSPDG SYVAVSSRGN FYLTWQPGQA FWQPHNRAVA
301 RRIQNMGWRA DGGLWLLVRG GGLFLSKGTG ITEEFEEVPV QSRGFGILDV
351 GYRSQDEAWA AGGSGVLLKT TNGGKTWIRE KAADNIAANL YSVKFINEKK
401 GFVLGNDGVL LQYLG
```

Start - End	Observed	Mr(expt)	Mr(calc)	ppm	Miss	Sequence
100 - 129	3264.8060	3263.7987	3263.7595	12	0	R.VYIPVDPGVVLLDIAFVPDDLNHGFLLGTR.Q
146 - 158	1556.7140	1555.7067	1555.6790	18	0	R.SIPSAEEEDFNYR.F
191 - 205	1676.9090	1675.9017	1675.8855	10	0	R.IPLSSQLPGDMVYIK.A Oxidation (M)
211 - 227	1872.8920	1871.8847	1871.8571	15	0	K.SAEMVTDEGAIYITSNR.G Oxidation (M)
233 - 245	1359.7410	1358.7337	1358.7154	14	0	R.AAVQETVSATLNR.T
246 - 266	2182.0840	2181.0767	2181.0338	20	0	R.TVSSGISGASYYTGTFNTVNR.S
310 - 319	1099.6340	1098.6267	1098.6186	7	0	R.ADGGLWLLVR.G
328 - 343	1777.8930	1776.8857	1776.8530	18	0	K.GTGITEEFEEVPVQSR.G
344 - 353	1096.5960	1095.5887	1095.5713	16	0	R.GFGILDVGYR.S
400 - 415	1692.9220	1691.9147	1691.9247	-6	1	K.KGFVLGNDGVLLQYLG.-

165.
Match to: **orange1.1g013804m|PAC:18122816** Score: **128** Expect: **2.1e-008**
Nominal mass (M_r): **46267**; Calculated pI value: **8.24**
NCBI BLAST search of orange1.1g013804m|PAC:18122816 against nr
Unformatted sequence string for pasting into other applications

Fixed modifications: Carbamidomethyl (C)
Variable modifications: Oxidation (M)
Cleavage by Trypsin: cuts C-term side of KR unless next residue is P
Number of mass values matched: **8**
Sequence Coverage: **30%**

Matched peptides shown in **Bold Red**

```
  1 MAYSLISSST FLLSRSPNTT LAPLNKHNFP LRPSRARATA GTIICCSNSP
 51 TTSAIRSIVS KLLLFTKPSS SASSAFESLF VFCGSVVLSF TLLFSNVDSA
101 SAFVVTPQRK LQTDELATVR LFQENTPSVV NITNLAARQD AFTLDVLEVP
151 QGSGSGFVWD SKGHVVTNYH VIRGASDIRV TFADQSAYDA KIVGFDQDKD
201 VAVLRIDAPK DKLRPIPIGV SADLLVGQKV YAIGNPFGLD HTLTTGVISG
251 LRREISSAAT GRPIQDVIQT DAAINPGNSG GPLLDSSGSL IGINTAIYSP
301 SGASSGVGFS IPVDTVNGIV DQLVKFGKVT RPILGIKFAP DQSVEQLGVS
```

351 **GVLVLDAPPN GPAGK**AGLLS TKRDAYGRLI LGDIITSVNG KKVSNGSDLY
401 RILDQCK**VGD EVIVEVLR**GD QKEKIPVKLE PKPDET

Start - End	Observed	Mr(expt)	Mr(calc)	ppm	Miss	Sequence
121 - 138	1987.0560	1986.0487	1986.0534	-2	0	R.LFQENTPSVVNITNLAAR.Q
163 - 173	1294.7040	1293.6967	1293.6942	2	0	K.GHVVTNYHVIR.G
192 - 205	1574.8520	1573.8447	1573.8464	-1	1	K.IVGFDQDKDVAVLR.I
213 - 229	1776.0710	1775.0637	1775.0669	-2	0	K.LRPIPIGVSADLLVGQK.V
230 - 252	2401.2750	2400.2677	2400.2802	-5	0	K.VYAIGNPFGLDHTLTTGVISGLR.R
329 - 337	996.6580	995.6507	995.6491	2	0	K.VTRPILGIK.F
338 - 365	2762.3760	2761.3687	2761.4287	-22	0	K.FAPDQSVEQLGVSGVLVLDAPPNGPAGK.A
408 - 418	1227.6910	1226.6837	1226.6871	-3	0	K.VGDEVIVEVLR.G

167.
Match to: **orange1.1g018105m|PAC:18133493** Score: **108** Expect: **2.1e-006**
Nominal mass (M_r): **38338**; Calculated pI value: **9.37**
NCBI BLAST search of orange1.1g018105m|PAC:18133493 against nr
Unformatted sequence string for pasting into other applications

Fixed modifications: Carbamidomethyl (C)
Variable modifications: Oxidation (M)
Cleavage by Trypsin: cuts C-term side of KR unless next residue is P
Number of mass values matched: **10**
Sequence Coverage: **38%**

Matched peptides shown in **Bold Red**

1 M**ASATAPTTL SLVK**TAASSS LSSPRASFLR MPTSASRRLG FSAADPLLTC
51 HVASRLSSIK TKGTRAVVSM AKKSVGELSG ADLKGKKVFV R**ADLNVPLDD**
101 **NQNITDDTRI R**AAVPTIKHL IQNGAK**VILS SHLGRPK**GVT PK**FSLAPLVP**
151 **R**LSELLGIQV VKADDCIGPE VEK**LVASLPE GGVLLLENVR** FYKEEEKNDP
201 EFAKK**LASLA DLYVNDAFGT AHR**AHASTEG VTK**YLKPSVA GFLLQK**ELDY
251 LVGAVSSPK**R PFAAIVGGSK** VSSKIGVIES LLETCDILLL GGGMIFTFYK
301 **AQGISVGSSL VEEDKLDLAT TLLAK**AKAKG VNLLLPSDVV IADKFAPDAN
351 SKVCFLNSYK

Start - End	Observed	Mr(expt)	Mr(calc)	ppm	Miss	Sequence
2 - 14	1259.6630	1258.6557	1258.7133	-46	0	M.ASATAPTTLSLVK.T
92 - 109	2028.9480	2027.9407	2027.9396	1	0	R.ADLNVPLDDNQNITDDTR.I
92 - 111	2298.1690	2297.1617	2297.1248	16	1	R.ADLNVPLDDNQNITDDTRIR.A
127 - 137	1206.7260	1205.7187	1205.7244	-5	0	K.VILSSHLGRPK.G
143 - 151	999.6020	998.5947	998.5913	3	0	K.FSLAPLVPR.L
174 - 190	1779.0390	1778.0317	1778.0302	1	0	K.LVASLPEGGVLLLENVR.F
206 - 223	1933.9760	1932.9687	1932.9694	-0	0	K.LASLADLYVNDAFGTAHR.A
234 - 246	1463.8520	1462.8447	1462.8548	-7	0	K.YLKPSVAGFLLQK.E
260 - 270	1102.6400	1101.6327	1101.6295	3	0	K.RPFAAIVGGSK.V

301 - 325	2558.3660	2557.3587	2557.3850	-10	1	K.AQGISVGSSLVEEDKLDLATTLLAK.A

170.
Match to: **clementine0.9_012385m|PAC:19264406** Score: **217** Expect: **2.7e-017**
Nominal mass (M_r): **43620**; Calculated pI value: **5.65**
NCBI BLAST search of clementine0.9_012385m|PAC:19264406 against nr
Unformatted sequence string for pasting into other applications

Fixed modifications: Carbamidomethyl (C)
Variable modifications: Oxidation (M)
Cleavage by Trypsin: cuts C-term side of KR unless next residue is P
Number of mass values matched: **26**
Sequence Coverage: **71%**

Matched peptides shown in **Bold Red**

```
  1 METFLFTSES VNEGHPDKLC DQISDAVLDA CLEQDPESKV ACETCTKTNM
 51 VMVFGEITTK AKVDYEKIVR DTCRSIGFVS DDVGLDADHC KVLVNIEQQS
101 PDIAQGVHGH FTKRPEDIGA GDQGHMFGYA TDETSEFMPL SHVLATKLGA
151 RLTEVRKNST CSWLRPDGKT QVTVEYYNDN GAMVPVRVHT VLISTQHDET
201 VTNDEIAADL KEHVIKPVIP EKYLDEKTIF HLNPSGRFVI GGPHGDAGLT
251 GRKIIIDTYG GWGAHGGGAF SGKDPTKVDR SGAYIVRQAA KSIVANGLAR
301 RCIVQVSYAI GVPEPLSVFV DSYGTGKIPD KEILKIVKES FDFRPGMMTI
351 NLDLKRGGNG RFLKTAAYGH FGRDDPDFTW EVVKPLKWEK PQA
```

Start - End	Observed	Mr(expt)	Mr(calc)	ppm	Miss	Sequence
19 - 39	2406.0970	2405.0897	2405.0726	7	0	K.LCDQISDAVLDACLEQDPESK.V
48 - 60	1486.7180	1485.7107	1485.7207	-7	0	K.TNMVMVFGEITTK.A Oxidation (M)
75 - 91	1834.8290	1833.8217	1833.8204	1	0	R.SIGFVSDDVGLDADHCK.V
92 - 113	2417.2520	2416.2447	2416.2499	-2	0	K.VLVNIEQQSPDIAQGVHGHFTK.R
114 - 147	3707.6540	3706.6467	3706.7032	-15	0	K.RPEDIGAGDQGHMFGYATDETSEFMPLSHVLATK.L
114 - 147	3723.6630	3722.6557	3722.6981	-11	0	K.RPEDIGAGDQGHMFGYATDETSEFMPLSHVLATK.L Oxidation (M)
114 - 147	3739.6500	3738.6427	3738.6930	-13	0	K.RPEDIGAGDQGHMFGYATDETSEFMPLSHVLATK.L 2 Oxidation (M)
170 - 187	2055.9700	2054.9627	2054.9731	-5	0	K.TQVTVEYYNDNGAMVPVR.V
170 - 187	2071.9670	2070.9597	2070.9681	-4	0	K.TQVTVEYYNDNGAMVPVR.V Oxidation (M)
188 - 222	3919.0370	3918.0297	3918.0738	-11	1	R.VHTVLISTQHDETVTNDEIAADLKEHVIKPVIPEK.Y
212 - 222	1288.7600	1287.7527	1287.7551	-2	0	K.EHVIKPVIPEK.Y
228 - 237	1141.6160	1140.6087	1140.6040	4	0	K.TIFHLNPSGR.F
238 - 252	1453.7570	1452.7497	1452.7474	2	0	R.FVIGGPHGDAGLTGR.K
238 - 253	1581.8160	1580.8087	1580.8423	-21	1	R.FVIGGPHGDAGLTGRK.I
253 - 273	2092.0340	2091.0267	2091.0538	-13	1	R.KIIIDTYGGWGAHGGGAFSGK.D
254 - 273	1963.9580	1962.9507	1962.9588	-4	0	K.IIIDTYGGWGAHGGGAFSGK.D
292 - 300	900.5260	899.5187	899.5188	-0	0	K.SIVANGLAR.R
302 - 327	2785.3730	2784.3657	2784.4044	-14	0	R.CIVQVSYAIGVPEPLSVFVDSYGTGK.I

339 - 355	2013.9660	2012.9587	2012.9700	-6	0	K.ESFDFRPGMMTINLDLK.R
339 - 355	2029.9620	2028.9547	2028.9649	-5	0	K.ESFDFRPGMMTINLDLK.R Oxidation (M)
339 - 355	2045.9630	2044.9557	2044.9598	-2	0	K.ESFDFRPGMMTINLDLK.R 2 Oxidation (M)
339 - 356	2186.0570	2185.0497	2185.0660	-7	1	K.ESFDFRPGMMTINLDLKR.G Oxidation (M)
339 - 356	2202.0530	2201.0457	2201.0609	-7	1	K.ESFDFRPGMMTINLDLKR.G 2 Oxidation (M)
365 - 373	979.4790	978.4717	978.4672	5	0	K.TAAYGHFGR.D
365 - 387	2649.3230	2648.3157	2648.3024	5	1	K.TAAYGHFGRDDPDFTWEVVKPLK.W
374 - 387	1688.8490	1687.8417	1687.8457	-2	0	R.DDPDFTWEVVKPLK.W

171.
Match to: **clementine0.9_013725m|PAC:19275240** Score: **119** Expect: **1.7e-007**
Nominal mass (M_r): **39923**; Calculated pI value: **6.33**
NCBI BLAST search of clementine0.9_013725m|PAC:19275240 against nr
Unformatted sequence string for pasting into other applications

Fixed modifications: Carbamidomethyl (C)
Variable modifications: Oxidation (M)
Cleavage by Trypsin: cuts C-term side of KR unless next residue is P
Number of mass values matched: **11**
Sequence Coverage: **36%**

Matched peptides shown in **Bold Red**

1 MGKGGMSQGE KKDGEEVNMA AWLMGVNTLK IQPFELPSLG PYDVLVRMKA
51 VGICGSDVHY LKTLRCADFV VK**EPMVIGHE CAGVIEK**VGS EVKTLVPGDR
101 VALEPGISCW RCDHCKGGRY NLCPEMKFFA TPPVHGSLAN QVVHPADLCF
151 K**LPDNVSLEE GAMCEPLSVG LHACR**RANIG PETNVLIMGA GPIGLVTMLG
201 ARAFGAPR**IV IVDVDDYR**LS VAKELGADNI VK**VSTNLQDI AEEVEK**IQKA
251 **MGTGIDVSFD CAGFNK**TMST ALSATRAGGK **VCLVGMGHHE MTVPLTPAAA**
301 **REVDVVGVFR** YK**NTWPLCLE FLR**SGKIDVK PLITHRFGFS QK**EVEEAFET**
351 **SAR**GGTAIKV MFNL

Start - End	Observed	Mr(expt)	Mr(calc)	ppm	Miss	Sequence
73 - 87	1668.8030	1667.7957	1667.8011	-3	0	K.EPMVIGHECAGVIEK.V
73 - 87	1684.8110	1683.8037	1683.7960	5	0	K.EPMVIGHECAGVIEK.V Oxidation (M)
152 - 175	2670.2220	2669.2147	2669.2247	-4	0	K.LPDNVSLEEGAMCEPLSVGLHACR.R Oxidation (M)
209 - 218	1206.6390	1205.6317	1205.6292	2	0	R.IVIVDVDDYR.L
233 - 246	1574.7940	1573.7867	1573.7835	2	0	K.VSTNLQDIAEEVEK.I
250 - 266	1805.7810	1804.7737	1804.7760	-1	0	K.AMGTGIDVSFDCAGFNK.T Oxidation (M)
281 - 301	2263.1070	2262.0997	2262.1072	-3	0	K.VCLVGMGHHEMTVPLTPAAAR.E Oxidation (M)
281 - 301	2279.1010	2278.0937	2278.1021	-4	0	K.VCLVGMGHHEMTVPLTPAAAR.E 2 Oxidation (M)
302 - 310	1019.5530	1018.5457	1018.5448	1	0	R.EVDVVGVFR.Y
313 - 323	1448.7350	1447.7277	1447.7282	-0	0	K.NTWPLCLEFLR.S
343 - 353	1267.5840	1266.5767	1266.5728	3	0	K.EVEEAFETSAR.G

172.
Match to: **orange1.1g043137m|PAC:18101066** Score: **235** Expect: **4.3e-019**
Nominal mass (M_r): **48059**; Calculated pI value: **5.54**
NCBI BLAST search of orange1.1g043137m|PAC:18101066 against nr
Unformatted sequence string for pasting into other applications

Fixed modifications: Carbamidomethyl (C)
Variable modifications: Oxidation (M)
Cleavage by Trypsin: cuts C-term side of KR unless next residue is P
Number of mass values matched: **20**
Sequence Coverage: **64%**

Matched peptides shown in **Bold Red**

```
  1 MAITITAVKA RQIFDSRGNP TVEVDVTTSD GHVARAAVPS GASTGIYEAL
 51 ELRDGGSDYL GKGVSKAVSN VNAIIGPALA GKDPTEQTAI DNYMVQQLDG
101 TVNEWGWCKQ KLGANAILAV SLAVCKAGAH VKKIPLYKHI AELSGNKNLV
151 LPVPAFNVIN GGSHAGNKLA MQEFMILPVG ASCFKEAMKM GVEVYHHLKA
201 VIKKKYGQDA TNVGDEGGFA PNIQENKEGL ELLNTAIAKA GYTGKVVIGM
251 DVAASEFYGS DKTYDLNFKE ENNDGSQKIS GDALKDLYKS FISDYPIVSI
301 EDPFDQDDWE HYAKLTSEVG EKVQIVGDDL LVTNPKRVEK AIKEKTCNAL
351 LLKVNQIGSV TESIEAVRMS KQAGWGVMAS HRSGETEDTF IADLSVGLAT
401 GQIKTGAPCR SERLAKYNQL LRIEEELGAE AVYAGAKFRA PVEPY
```

Start - End	Observed	Mr(expt)	Mr(calc)	ppm	Miss	Sequence
18 - 35	1853.8960	1852.8887	1852.8916	-2	0	R.GNPTVEVDVTTSDGHVAR.A
36 - 53	1804.9430	1803.9357	1803.9366	-1	0	R.AAVPSGASTGIYEALELR.D
67 - 82	1494.8550	1493.8477	1493.8566	-6	0	K.AVSNVNAIIGPALAGK.D
112 - 126	1499.8580	1498.8507	1498.8541	-2	0	K.LGANAILAVSLAVCK.A
148 - 168	2118.1280	2117.1207	2117.1382	-8	0	K.NLVLPVPAFNVINGGSHAGNK.L
169 - 185	1973.9560	1972.9487	1972.9461	1	0	K.LAMQEFMILPVGASCFK.E 2 Oxidation (M)
190 - 199	1228.6150	1227.6077	1227.6070	1	0	K.MGVEVYHHLK.A Oxidation (M)
205 - 227	2452.1570	2451.1497	2451.1302	8	1	K.KYGQDATNVGDEGGFAPNIQENK.E
206 - 227	2324.0450	2323.0377	2323.0353	1	0	K.YGQDATNVGDEGGFAPNIQENK.E
246 - 262	1787.8710	1786.8637	1786.8448	11	0	K.VVIGMDVAASEFYGSDK.T
263 - 278	1901.8700	1900.8627	1900.8439	10	1	K.TYDLNFKEENNDGSQK.I
290 - 314	3016.3310	3015.3237	3015.3450	-7	0	K.SFISDYPIVSIEDPFDQDDWEHYAK.L
323 - 336	1510.8420	1509.8347	1509.8403	-4	0	K.VQIVGDDLLVTNPK.R
354 - 368	1601.8490	1600.8417	1600.8420	-0	0	K.VNQIGSVTESIEAVR.M
372 - 382	1199.5720	1198.5647	1198.5666	-2	0	K.QAGWGVMASHR.S
372 - 382	1215.5700	1214.5627	1214.5615	1	0	K.QAGWGVMASHR.S Oxidation (M)
383 - 404	2252.1190	2251.1117	2251.1220	-5	0	R.SGETEDTFIADLSVGLATGQIK.T
417 - 422	806.4560	805.4487	805.4446	5	0	K.YNQLLR.I
423 - 437	1549.7610	1548.7537	1548.7671	-9	0	R.IEEELGAEAVYAGAK.F
438 - 445	978.5010	977.4937	977.4971	-3	1	K.FRAPVEPY.-

173.
Match to: **orange1.1g008774m|PAC:18106813** Score: **170** Expect: **1.3e-012**
Nominal mass (M_r): **61119**; Calculated pI value: **5.65**
NCBI BLAST search of orange1.1g008774m|PAC:18106813 against nr
Unformatted sequence string for pasting into other applications

Fixed modifications: Carbamidomethyl (C)
Variable modifications: Oxidation (M)
Cleavage by Trypsin: cuts C-term side of KR unless next residue is P
Number of mass values matched: **16**
Sequence Coverage: **42%**

Matched peptides shown in **Bold Red**

```
  1 MDNWKLKEHP QLSRNNIVAV VVLDGWGEFK PDKYNCIHVA DTPTMDSFKK
 51 SAPGRWRLLR AHGSAVGLPT EDDMGNSEVG HNALGAGRIF AQGAKLVDLA
101 LASGKIYQDE GFNYIKPSFE TGTLHLIGLL SDGGVHSRLD QLQLLLKGAS
151 ERGAKRIRLH ILTDGRDVLD GSSVGFVETI EKDLAELRGK GVDAQIASGG
201 GRMYVTMDRY ENDWDVVKRG WDAQVLGEAP HKFKSAVEAV KKLREQPNAN
251 DQYLPPFVIV DENGKAVGPI VDGDAVVTFN FRADRMVMLA KALEYEDFDK
301 FDRVRFPKIR YAGMLQYDGE LKLPSHYLVS PPEIDRTSGE YLVHNGVRTF
351 ACSETVKFGH VTFFWNGNRS GYFDSNLEEY VEIPSDSGIT FNVQPKMKAL
401 EIAERAKKAI LSRRFHQVRV NLPNSDMVGH TGDIEATVVA CKAADEAVKI
451 IIDAIEKVGG IYLVTADHGN AEDMVKRNKS GEPLLKDGNI QVLTSHTLKP
501 VPIAIGGPGL PDDVKFRTDL PNGGLANVAA TFINLHGYEA PSDYEPSLIE
551 VTDD
```

Start - End	Observed	Mr(expt)	Mr(calc)	ppm	Miss	Sequence
6 - 14	1107.6250	1106.6177	1106.6196	-2	1	K.LKEHPQLSR.N
61 - 88	2735.2450	2734.2377	2734.2365	0	0	R.AHGSAVGLPTEDDMGNSEVGHNALGAGR.I Oxidation (M)
159 - 166	924.5360	923.5287	923.5189	11	0	R.LHILTDGR.D
191 - 202	1087.5410	1086.5337	1086.5418	-7	0	K.GVDAQIASGGGR.M
219 - 232	1563.7800	1562.7727	1562.7954	-14	1	K.RGWDAQVLGEAPHK.F
220 - 232	1407.6980	1406.6907	1406.6943	-3	0	R.GWDAQVLGEAPHK.F
245 - 265	2387.1240	2386.1167	2386.1441	-11	0	R.EQPNANDQYLPPFVIVDENGK.A
266 - 282	1776.9180	1775.9107	1775.9207	-6	0	K.AVGPIVDGDAVVTFNFR.A
286 - 300	1818.9020	1817.8947	1817.8579	20	1	R.MVMLAKALEYEDFDK.F Oxidation (M)
292 - 303	1547.7060	1546.6987	1546.6940	3	1	K.ALEYEDFDKFDR.V
323 - 336	1622.8470	1621.8397	1621.8464	-4	0	K.LPSHYLVSPPEIDR.T
337 - 348	1331.6630	1330.6557	1330.6630	-5	0	R.TSGEYLVHNGVR.T
358 - 369	1481.7050	1480.6977	1480.7000	-2	0	K.FGHVTFFWNGNR.S
420 - 442	2443.1550	2442.1477	2442.1519	-2	0	R.VNLPNSDMVGHTGDIEATVVACK.A Oxidation (M)
458 - 476	2004.9620	2003.9547	2003.9623	-4	0	K.VGGIYLVTADHGNAEDMVK.R Oxidation (M)
487 - 515	2951.5900	2950.5827	2950.6128	-10	0	K.DGNIQVLTSHTLKPVPIAIGGPGLPDDVK.F

178.
Match to: **orange1.1g045706m|PAC:18132194** Score: **68** Expect: **0.019**
Nominal mass (M_r): **14819**; Calculated pI value: **6.92**
NCBI BLAST search of orange1.1g045706m|PAC:18132194 against nr
Unformatted sequence string for pasting into other applications

Fixed modifications: Carbamidomethyl (C)
Variable modifications: Oxidation (M)
Cleavage by Trypsin: cuts C-term side of KR unless next residue is P
Number of mass values matched: **4**
Sequence Coverage: **35%**

Matched peptides shown in **Bold Red**

1 VGEIISRFEK KGFKLIGLKL FQCPKDLAEE HYKDLNSKPF FPKLIEYITS
51 GPVVCMAWEG AGVVASAR**KL IGSTDPLQAE PGTIR**GDLAV QTGR**NVVHGS**
101 **DSPENGKR**EI GLWFK**EGELC QWTPAQAQWL R**E

Start - End	Observed	Mr(expt)	Mr(calc)	ppm	Miss	Sequence
69 - 85	1795.9600	1794.9527	1794.9839	-17	1	R.KLIGSTDPLQAEPGTIR.G
70 - 85	1667.8840	1666.8767	1666.8890	-7	0	K.LIGSTDPLQAEPGTIR.G
95 - 108	1495.7130	1494.7057	1494.7175	-8	1	R.NVVHGSDSPENGKR.E
116 - 131	1972.9520	1971.9447	1971.9261	9	0	K.EGELCQWTPAQAQWLR.E

180.
Match to: **gi|224365649|ref|YP_002608376.1|** Score: **51** Expect: **1.2**
PSI 9 kDa protein [Vitis vinifera]

Nominal mass (M_r): **9545**; Calculated pI value: **6.67**
NCBI BLAST search of gi|224365649|ref|YP_002608376.1| against nr
Unformatted sequence string for pasting into other applications

Fixed modifications: Carbamidomethyl (C)
Variable modifications: Oxidation (M)
Cleavage by Trypsin: cuts C-term side of KR unless next residue is P
Number of mass values matched: **3**
Sequence Coverage: **43%**

Matched peptides shown in **Bold Red**

1 MSHSVK**IYDT CIGCTQCVR**A CPTDVLEMIP WDGCKAKQIA SAPRTEDCVG
51 CKR**CESACPT DFLSVRVYLW HETTR**SMGLA Y

Start - End	Observed	Mr(expt)	Mr(calc)	ppm	Miss	Sequence
**7 - 19**	**1645.7170**	**1644.7097**	**1644.7059**	**2**	**0**	**K.IYDTCIGCTQCVR.A**
**54 - 66**	**1541.6600**	**1540.6527**	**1540.6650**	**-8**	**0**	**R.CESACPTDFLSVR.V**
**67 - 75**	**1204.6110**	**1203.6037**	**1203.6037**	**0**	**0**	**R.VYLWHETTR.S**

185.
Match to: **orange1.1g028155m|PAC:18095430** Score: **110** Expect: **1.3e-006**

Nominal mass (M_r): **23962**; Calculated pI value: **6.18**
NCBI BLAST search of orange1.1g028155m|PAC:18095430 against nr
Unformatted sequence string for pasting into other applications

Fixed modifications: Carbamidomethyl (C)
Variable modifications: Oxidation (M)
Cleavage by Trypsin: cuts C-term side of KR unless next residue is P
Number of mass values matched: **8**
Sequence Coverage: **45%**

Matched peptides shown in **Bold Red**

1 MAVEICVK**AA VGAPDILGDC PFSQR**ALLTL EEKKVPYKRH LINISDKPQW
51 FMEISPEGKV PVVK**FDDKWV ADSDVIVR**II EEKYPEPSLT NPPEFASLGS
101 KIFPSFVNFL K**SKDPNDGTE QALLEELK**AL DEHLK**THGGP FIAGEK**VTAV
151 DLSLAPK**LYH LQVALEHFKQ WTVPESLAHV HGYTKKLFAL ESFQK**TKAEK
201 QYVIAGWVPK VNA

Start - End	Observed	Mr(expt)	Mr(calc)	ppm	Miss	Sequence
**9 - 25**	**1773.8520**	**1772.8447**	**1772.8516**	**-4**	**0**	**K.AAVGAPDILGDCPFSQR.A**
**65 - 78**	**1664.8210**	**1663.8137**	**1663.8206**	**-4**	**1**	**K.FDDKWVADSDVIVR.I**
**69 - 78**	**1159.6050**	**1158.5977**	**1158.6033**	**-5**	**0**	**K.WVADSDVIVR.I**
**112 - 128**	**1886.9230**	**1885.9157**	**1885.9269**	**-6**	**1**	**K.SKDPNDGTEQALLEELK.A**
**136 - 146**	**1113.5680**	**1112.5607**	**1112.5615**	**-1**	**0**	**K.THGGPFIAGEK.V**
**158 - 169**	**1497.8190**	**1496.8117**	**1496.8140**	**-1**	**0**	**K.LYHLQVALEHFK.Q**
**170 - 185**	**1852.9230**	**1851.9157**	**1851.9268**	**-6**	**0**	**K.QWTVPESLAHVHGYTK.K**
**186 - 195**	**1210.6760**	**1209.6687**	**1209.6757**	**-6**	**1**	**K.KLFALESFQK.T**

186.
Match to: **orange1.1g025540m|PAC:18138357** Score: **150** Expect: **1.3e-010**

Nominal mass (M_r): **27160**; Calculated pI value: **5.74**
NCBI BLAST search of orange1.1g025540m|PAC:18138357 against nr
Unformatted sequence string for pasting into other applications

Fixed modifications: Carbamidomethyl (C)

Variable modifications: Oxidation (M)
Cleavage by Trypsin: cuts C-term side of KR unless next residue is P
Number of mass values matched: **11**
Sequence Coverage: **65%**

Matched peptides shown in **Bold Red**

```
  1 MGRKFFVGGN WKCNGTPEEV KKIVSVLNEG QVPSSDVVEV VVSPPFVFLG
 51 LVKSSLRPGF HVAAQNCWVK KGGAFTGEIS AEMLVNLEIP WVILGHSERR
101 LILNELNEFV GDKVAYALSQ GLKVIACVGE TLEQREAGST MDVVAAQTKA
151 IADRVSSWSN IVLAYEPVWA IGTGKVATPA QAQEVHFELR KWLLANTSPE
201 IAAATRIIYG GSVNGANCKE LAAQPDVDGF LVGGASLKPE FIDIIKSAEL
251 K
```

Start - End	Observed	Mr(expt)	Mr(calc)	ppm	Miss	Sequence
54 - 70	1957.0150	1956.0077	1955.9788	15	0	K.SSLRPGFHVAAQNCWVK.K
71 - 99	3169.6490	3168.6417	3168.6277	4	1	K.KGGAFTGEISAEMLVNLEIPWVILGHSER.R Oxidation (M)
100 - 113	1659.9250	1658.9177	1658.8991	11	1	R.RLILNELNEFVGDK.V
101 - 113	1503.8290	1502.8217	1502.7980	16	0	R.LILNELNEFVGDK.V
124 - 135	1374.7210	1373.7137	1373.6973	12	0	K.VIACVGETLEQR.E
155 - 175	2277.2230	2276.2157	2276.1841	14	0	R.VSSWSNIVLAYEPVWAIGTGK.V
176 - 190	1695.9120	1694.9047	1694.8740	18	0	K.VATPAQAQEVHFELR.K
176 - 191	1824.0050	1822.9977	1822.9690	16	1	K.VATPAQAQEVHFELRK.W
192 - 206	1613.8930	1612.8857	1612.8573	18	0	K.WLLANTSPEIAAATR.I
207 - 219	1352.6830	1351.6757	1351.6554	15	0	R.IIYGGSVNGANCK.E
220 - 246	2842.5770	2841.5697	2841.5164	19	0	K.ELAAQPDVDGFLVGGASLKPEFIDIIK.S

188.
Match to: **orange1.1g019034m|PAC:18135677** Score: **162** Expect: **8.5e-012**
Nominal mass (M_r): **39324**; Calculated pI value: **7.66**
NCBI BLAST search of orange1.1g019034m|PAC:18135677 against nr
Unformatted sequence string for pasting into other applications

Fixed modifications: Carbamidomethyl (C)
Variable modifications: Oxidation (M)
Cleavage by Trypsin: cuts C-term side of KR unless next residue is P
Number of mass values matched: **17**
Sequence Coverage: **47%**

Matched peptides shown in **Bold Red**

```
  1 MPPTTAVSAS SSFTLFRVPS SWSTKLKPTT TYIQIPNRFF PKHPTFKMTT
 51 TTIRAAVSIE KETPETERPP TFLRESDDKE SSSSSASSVR ARFEKMIRDA
101 QDSVCQAIEK TDGGGKFKED VWSRPGGGGG ISRVLQDGAI WEKAGVNVSV
151 VYGVMPPEAY RAAKAAASDE KPGPIPFFAA GISSVLHPKN PFAPTLHFNY
201 RYFETDAPKD TPGAPRQWWF GGGTDLTPAY IFEEDVKHFH STQKSACDKF
```

251 **DPTFYPRFK**K WCDDYFYIKH RGERRGLGGL FFDDLNDYDQ EMLLSFATEC
301 ANSVIPAYIP IIEKR**KDTPF** **TDQHKAWQQL** **R**RGR**YVEFNL** **VSNSPED**

Start - End	Observed	Mr(expt)	Mr(calc)	ppm	Miss	Sequence
43 - 54	1433.7660	1432.7587	1432.7497	6	1	K.HPTFKMTTTTIR.A
62 - 74	1572.7990	1571.7917	1571.7944	-2	0	K.ETPETERPPTFLR.E
75 - 90	1657.7630	1656.7557	1656.7074	29	1	R.ESDDKESSSSSASSVR.A
117 - 133	1804.8990	1803.8917	1803.9016	-5	1	K.FKEDVWSRPGGGGGISR.V
134 - 143	1158.6090	1157.6017	1157.6081	-5	0	R.VLQDGAIWEK.A
144 - 161	1907.9560	1906.9487	1906.9611	-7	0	K.AGVNVSVVYGVMPPEAYR.A
144 - 161	1923.9520	1922.9447	1922.9560	-6	0	K.AGVNVSVVYGVMPPEAYR.A Oxidation (M)
144 - 164	2194.0710	2193.0637	2193.1252	-28	1	K.AGVNVSVVYGVMPPEAYRAAK.A Oxidation (M)
190 - 201	1476.7400	1475.7327	1475.7310	1	0	K.NPFAPTLHFNYR.Y
217 - 237	2459.1420	2458.1347	2458.1481	-5	0	R.QWWFGGGTDLTPAYIFEEDVK.H
245 - 257	1603.7190	1602.7117	1602.7137	-1	1	K.SACDKFDPTFYPR.F
250 - 257	1042.5010	1041.4937	1041.4920	2	0	K.FDPTFYPR.F
250 - 259	1317.6470	1316.6397	1316.6554	-12	1	K.FDPTFYPRFK.K
316 - 325	1216.5900	1215.5827	1215.5884	-5	1	R.KDTPFTDQHK.A
317 - 325	1088.4910	1087.4837	1087.4935	-9	0	K.DTPFTDQHK.A
326 - 331	801.4320	800.4247	800.4293	-6	0	K.AWQQLR.R
335 - 347	1512.7310	1511.7237	1511.6780	30	0	R.YVEFNLVSNSPED.-

189.
Match to: **clementine0.9_016525m|PAC:19269127** Score: **54** Expect: **0.57**
Nominal mass (M_r): **34281**; Calculated pI value: **5.92**
NCBI BLAST search of clementine0.9_016525m|PAC:19269127 against nr
Unformatted sequence string for pasting into other applications

Fixed modifications: Carbamidomethyl (C)
Variable modifications: Oxidation (M)
Cleavage by Trypsin: cuts C-term side of KR unless next residue is P
Number of mass values matched: **4**
Sequence Coverage: **19%**

Matched peptides shown in **Bold Red**

1 MASKSKILSI GGTGYIGKFI VEASVK**AGHP** **TFVLVR**ESTL SAPSKSQLLD
51 HFKNLGVNFV VGDVLNHESL VNAIKQVDVV ISTVGHALLA DQVKIIAAIK
101 EAGNVKR**FFP** **SEFGNDVDR**A HGAVEPAKSA YYDVKARIRR AVEAEGIPYT
151 YVESYCFDGY FLPNLLQPGA AAPPRDKVVI LGDGNPRAVY NKEDDIATYT
201 IKAVDDPRTL NK**NLYIQPPG** **NIYSFNDLVS** **LWER**KIGKTL EREYVSEEQL
251 LKNIQEAAPP QNVILSIYHS VFMNGVQTNF K**IEPSFGVEA** **SQLYPDVK**YT
301 TVDEYLNQSV

Start - End	Observed	Mr(expt)	Mr(calc)	ppm	Miss	Sequence

27 - 36	1096.6390	1095.6317	1095.6189	12	0	K.AGHPTFVLVR.E
108 - 119	1429.6590	1428.6517	1428.6310	14	0	R.FFPSEFGNDVDR.A
213 - 234	2638.3840	2637.3767	2637.3227	20	0	K.NLYIQPPGNIYSFNDLVSLWER.K
282 - 298	1878.9780	1877.9707	1877.9411	16	0	K.IEPSFGVEASQLYPDVK.Y

191.
Match to: **clementine0.9_015365m|PAC:19257524** Score: **196** Expect: **3.4e-015**
Nominal mass (M_r): **35859**; Calculated pI value: **6.10**
NCBI BLAST search of clementine0.9_015365m|PAC:19257524 against nr
Unformatted sequence string for pasting into other applications

Fixed modifications: Carbamidomethyl (C)
Variable modifications: Oxidation (M)
Cleavage by Trypsin: cuts C-term side of KR unless next residue is P
Number of mass values matched: **19**
Sequence Coverage: **66%**

Matched peptides shown in **Bold Red**

```
  1 MAKEPVRVLV TGAAGQIGYA LVPMIARGVM LGTDQPVILH MLDIPPAAEA
 51 LNGVKMELVD AAFPLLKGVV ATTDAVEACT GVNIAVMVGG FPRKEGMERK
101 DVMSKNVSIY KAQASALEQH AAPNCKVLVV ANPANTNALI LKEFAPSIPA
151 KNITCLTRLD HNRALGQISE KLNVQVSDVK NVIIWGNHSS SQYPDVNHAT
201 VNTAAGEKPV RELVKDDAWL NGEFITTVQQ RGAAIIKARK LSSALSAASS
251 ACDHIRDWVL GTPEGTWVSM GVYSDGSYNV PAGLIYSFPV TCRNGEWTIV
301 QGLSIDEFSR KKLDLTAEEL SEEKALAYSC LS
```

Start - End	Observed	Mr(expt)	Mr(calc)	ppm	Miss	Sequence
8 - 27	2000.1140	1999.1067	1999.1288	-11	0	R.VLVTGAAGQIGYALVPMIAR.G
8 - 27	2016.1220	2015.1147	2015.1238	-4	0	R.VLVTGAAGQIGYALVPMIAR.G Oxidation (M)
28 - 55	2899.5000	2898.4927	2898.5347	-14	0	R.GVMLGTDQPVILHMLDIPPAAEALNGVK.M
28 - 55	2915.5020	2914.4947	2914.5296	-12	0	R.GVMLGTDQPVILHMLDIPPAAEALNGVK.M Oxidation (M)
28 - 55	2931.4940	2930.4867	2930.5246	-13	0	R.GVMLGTDQPVILHMLDIPPAAEALNGVK.M 2 Oxidation (M)
56 - 67	1346.7290	1345.7217	1345.7315	-7	0	K.MELVDAAFPLLK.G
56 - 67	1362.7280	1361.7207	1361.7265	-4	0	K.MELVDAAFPLLK.G Oxidation (M)
68 - 93	2607.2690	2606.2617	2606.2833	-8	0	K.GVVATTDAVEACTGVNIAVMVGGFPR.K Oxidation (M)
112 - 126	1595.7550	1594.7477	1594.7522	-3	0	K.AQASALEQHAAPNCK.V
127 - 142	1649.9870	1648.9797	1648.9876	-5	0	K.VLVVANPANTNALILK.E
152 - 158	877.4550	876.4477	876.4487	-1	0	K.NITCLTR.L
181 - 211	3361.6370	3360.6297	3360.6599	-9	0	K.NVIIWGNHSSSQYPDVNHATVNTAAGEKPVR.E
212 - 231	2362.1930	2361.1857	2361.1965	-5	1	R.ELVKDDAWLNGEFITTVQQR.G
216 - 231	1892.9030	1891.8957	1891.9064	-6	0	K.DDAWLNGEFITTVQQR.G
240 - 256	1773.8840	1772.8767	1772.8839	-4	1	R.KLSSALSAASSACDHIR.D
241 - 256	1645.7950	1644.7877	1644.7889	-1	0	K.LSSALSAASSACDHIR.D
294 - 310	1950.9610	1949.9537	1949.9483	3	0	R.NGEWTIVQGLSIDEFSR.K

**312 - 324**	**1504.7620**	**1503.7547**	**1503.7668**	**-8**	**1**	**K.KLDLTAEELSEEK.A**
**313 - 324**	**1376.6780**	**1375.6707**	**1375.6718**	**-1**	**0**	**K.LDLTAEELSEEK.A**

197.
Match to: **clementine0.9_012376m|PAC:19276938** Score: **68** Expect: **0.023**
Nominal mass (M_r): **44041**; Calculated pI value: **5.98**
NCBI BLAST search of clementine0.9_012376m|PAC:19276938 against nr
Unformatted sequence string for pasting into other applications

Fixed modifications: Carbamidomethyl (C)
Variable modifications: Oxidation (M)
Cleavage by Trypsin: cuts C-term side of KR unless next residue is P
Number of mass values matched: **7**
Sequence Coverage: **24%**

Matched peptides shown in **Bold Red**

```
  1 MPATDYQGSF LGRISIRRNQ VMSMEGNHEQ ELEDLELFQK HVADRFAELA
 51 PPSPSQPSDD IPSDPILSIA WIRKLLDVFL CCDAEFKAIL IMGRDPSQIS
101 KPPLDRLIPE MLDRDVKALD ICNAITNGIE AVRHYQKLAE IAVSALEQRP
151 FGDGQVKRAR KALTSLITAM ACEDKDGANY TRTWSFGRRG AAAAGNNKST
201 GQLRSLSWSV SKNWSAAKQI HAMSANLVAP RGAEASGLAL PVYIMSTILL
251 FVMWTMVATI PCQERSGLAT HFPVPKQLVW AQSIIGLHEK IAEEWKKKEK
301 KGSAGLLEEM QKLDKLGQSL IEFTDSFQFP AEDERAEEVS AHLAELGETC
351 RRMEEGLVPL QVQIREVFHR IVRSRAEFLD VLDLNGKSSA LAM
```

Start - End	Observed	Mr(expt)	Mr(calc)	ppm	Miss	Sequence
**1 - 13**	**1442.6930**	**1441.6857**	**1441.6660**	**14**	**0**	**-.MPATDYQGSFLGR.I**
**1 - 17**	**1911.9230**	**1910.9157**	**1910.9673**	**-27**	**1**	**-.MPATDYQGSFLGRISIR.R**
**2 - 17**	**1780.8490**	**1779.8417**	**1779.9268**	**-48**	**1**	**M.PATDYQGSFLGRISIR.R**
**88 - 106**	**2123.0960**	**2122.0887**	**2122.1568**	**-32**	**1**	**K.AILIMGRDPSQISKPPLDR.L** Oxidation (M)
**138 - 158**	**2284.1490**	**2283.1417**	**2283.2335**	**-40**	**1**	**K.LAEIAVSALEQRPFGDGQVKR.A**
**161 - 175**	**1667.7790**	**1666.7717**	**1666.8270**	**-33**	**1**	**R.KALTSLITAMACEDK.D** Oxidation (M)
**313 - 335**	**2685.3450**	**2684.3377**	**2684.2970**	**15**	**1**	**K.LDKLGQSLIEFTDSFQFPAEDER.A**

198.
Match to: **clementine0.9_008926m|PAC:19259027** Score: **92** Expect: **9.3e-005**
Nominal mass (M_r): **54000**; Calculated pI value: **6.00**
NCBI BLAST search of clementine0.9_008926m|PAC:19259027 against nr
Unformatted sequence string for pasting into other applications

Fixed modifications: Carbamidomethyl (C)
Variable modifications: Oxidation (M)
Cleavage by Trypsin: cuts C-term side of KR unless next residue is P

Number of mass values matched: **13**
Sequence Coverage: **34%**

Matched peptides shown in **Bold Red**

```
  1 MAPKALDYES MNENVKKVQY AVRGELYLRA SELQKEGKKI IFTNVGNPHA
 51 LGQRPLTFPR QVVALCQAPF LLDDPNVGIV FPADAIARAK HYLSLTSGGL
101 GAYSDSRGIP GVRKEVAEFI ERRDGYPSDP ELIFLTDGAS KGVMQTLNCV
151 IRGEGDGVLV PVPQYPLYSA TISLLGGSLV PYYLEETANW GLDLNDLQQS
201 VAQARSKGIT VRAMVIINPG NPTGQCLSEA NLREILRFCY QENLVLLGDE
251 VYQQNIYQDE RPFVSSKKVL MDMGPPFSKE VQLVSFHTVS KGYWGECGQR
301 GGYFEMTNIP PKTVDEIYKI ASIALSPNVP AQIFMGLMVN PLKPGDISYE
351 QFVRESKGIL ESLRRRARMM TDGFNSCRNV VCNFTEGAMY SFPQIRLPPK
401 AIEAAKRAGK VPDVFYCLRL LEATGISTVP GSGFGQKEGV FHLRTTILPA
451 EEDMPAIMES FKKFNDEFME QYEDYRGYSR M
```

Start - End	Observed	Mr(expt)	Mr(calc)	ppm	Miss	Sequence	
91 - 107	1783.8400	1782.8327	1782.8537	-12	0	K.HYLSLTSGGLGAYSDSR.G	
114 - 122	1120.5780	1119.5707	1119.5924	-19	1	R.KEVAEFIER.R	
115 - 122	992.5090	991.5017	991.4974	4	0	K.EVAEFIER.R	
213 - 233	2255.0790	2254.0717	2254.1198	-21	0	R.AMVIINPGNPTGQCLSEANLR.E	
213 - 233	2271.0700	2270.0627	2270.1147	-23	0	R.AMVIINPGNPTGQCLSEANLR.E	Oxidation (M)
238 - 267	3681.6950	3680.6877	3680.7457	-16	0	R.FCYQENLVLLGDEVYQQNIYQDERPFVSSK.K	
280 - 291	1373.7190	1372.7117	1372.7351	-17	0	K.EVQLVSFHTVSK.G	
292 - 300	1112.4630	1111.4557	1111.4505	5	0	K.GYWGECGQR.G	
301 - 312	1369.6460	1368.6387	1368.6384	0	0	R.GGYFEMTNIPPK.T	Oxidation (M)
379 - 396	2148.9810	2147.9737	2147.9769	-1	0	R.NVVCNFTEGAMYSFPQIR.L	Oxidation (M)
411 - 419	1168.5790	1167.5717	1167.5747	-3	0	K.VPDVFYCLR.L	
420 - 437	1761.8990	1760.8917	1760.9309	-22	0	R.LLEATGISTVPGSGFGQK.E	
464 - 476	1801.7270	1800.7197	1800.6937	14	0	K.FNDEFMEQYEDYR.G	Oxidation (M)

199.
Match to: **clementine0.9_009022m|PAC:19276540** Score: **198** Expect: **2.1e-015**
Nominal mass (M_r): **52969**; Calculated pI value: **5.92**
NCBI BLAST search of clementine0.9_009022m|PAC:19276540 against nr
Unformatted sequence string for pasting into other applications

Fixed modifications: Carbamidomethyl (C)
Variable modifications: Oxidation (M)
Cleavage by Trypsin: cuts C-term side of KR unless next residue is P
Number of mass values matched: **19**
Sequence Coverage: **52%**

Matched peptides shown in **Bold Red**

```
  1 MSPMLETCLA LGKTFPVYDP RTAEVIANVA EGDAEDIDRA VATARKAFDE
 51 GPWPKMTPYE RSRIMLRAAD LIEKNMEELA ALETWNNGKP YVQSLKSEVP
101 MVVRLLHYYA GWADKIHGLT VPGDGNYHIQ TLHEPIGVAG QIVPWNFPLL
151 LFTWKVAPAL TCGNTIVLKS AEQTPLTALY VAKLFHEAGL PPGVLNVVSG
201 YGPTAGAALA SHMDVDKLSF TGHCDTGKIV QELAAKSNLK PVTLELGGKS
251 PFIIFDDADV DQAVELAHFA LFYNQGQCCC AGSRTYVHER VYDEFVEKAK
301 ARAMRRIVGD PFKSGVEQGP QIDPEQFEKV LRYIRSGIES NATLECGGDR
351 LGNRGYFVQP TVFSDVKDDM LIAQDEIFGP VQSILKFKET DDVIRRANKT
401 RFGLAAGVFT KSADTANTMM RALRAGTVWI NCYDVFDAAI PFGGYKLSGI
451 GREKGIYSLN AYLQVKAVVQ PVKNPAWL
```

Start - End	Observed	Mr(expt)	Mr(calc)	ppm	Miss	Sequence	
14 - 21	994.5000	993.4927	993.4920	1	0	K.TFPVYDPR.T	
22 - 39	1887.8830	1886.8757	1886.8857	-5	0	R.TAEVIANVAEGDAEDIDR.A	
46 - 55	1174.5880	1173.5807	1173.5818	-1	1	R.KAFDEGPWPK.M	
97 - 104	916.4870	915.4797	915.4848	-6	0	K.SEVPMVVR.L	
105 - 115	1336.6690	1335.6617	1335.6611	0	0	R.LLHYYAGWADK.I	
170 - 183	1491.7810	1490.7737	1490.7980	-16	0	K.SAEQTPLTALYVAK.L	
184 - 217	3406.6570	3405.6497	3405.7027	-16	0	K.LFHEAGLPPGVLNVVSGYGPTAGAALASHMDVDK.L	Oxidation (M)
218 - 228	1222.5530	1221.5457	1221.5448	1	0	K.LSFTGHCDTGK.I	
237 - 249	1355.7800	1354.7727	1354.7820	-7	0	K.SNLKPVTLELGGK.S	
285 - 290	804.4040	803.3967	803.3926	5	0	R.TYVHER.V	
314 - 329	1787.8350	1786.8277	1786.8374	-5	0	K.SGVEQGPQIDPEQFEK.V	
314 - 332	2156.1380	2155.1307	2155.0909	18	1	K.SGVEQGPQIDPEQFEKVLR.Y	
336 - 350	1565.6780	1564.6707	1564.6787	-5	0	R.SGIESNATLECGGDR.L	
355 - 367	1486.7320	1485.7247	1485.7504	-17	0	R.GYFVQPTVFSDVK.D	
355 - 386	3615.7410	3614.7337	3614.8219	-24	1	R.GYFVQPTVFSDVKDDMLIAQDEIFGPVQSILK.F	Oxidation (M)
387 - 395	1122.5770	1121.5697	1121.5717	-2	1	K.FKETDDVIR.R	
402 - 411	1010.5310	1009.5237	1009.5597	-36	0	R.FGLAAGVFTK.S	
425 - 446	2464.1440	2463.1367	2463.1569	-8	0	R.AGTVWINCYDVFDAAIPFGGYK.L	
455 - 466	1368.7360	1367.7287	1367.7449	-12	0	K.GIYSLNAYLQVK.A	

202.
Match to: **clementine0.9_021650m|PAC:19268496** Score: **126** Expect: **3.4e-008**
Nominal mass (M_r): **23610**; Calculated pI value: **8.18**
NCBI BLAST search of clementine0.9_021650m|PAC:19268496 against nr
Unformatted sequence string for pasting into other applications

Fixed modifications: Carbamidomethyl (C)
Variable modifications: Oxidation (M)
Cleavage by Trypsin: cuts C-term side of KR unless next residue is P
Number of mass values matched: **9**
Sequence Coverage: **54%**

Matched peptides shown in **Bold Red**

1 MSTPFVTAIS FLLLTFATKP LVGLADPLVD VNGNKVEASR **DYYLVSAIRG**
51 **AGGGGLTLFR GRNELCPLDV VQLSSDSER**G TRLRFSMSDK **TSIINEDVDL**
101 **NVR**FSTETR**C NEPTVWR**VDS YDPSRGK**WFI TTGGVEGNPG AQTLK**NWFKF
151 ERIGRDRATY K**IVHCPSVCE SCVSLCNDVG VSNDHAR**RLA LTNGR**ALAVV**
201 **LVPGNER**SAS CAS

Start - End	Observed	Mr(expt)	Mr(calc)	ppm	Miss	Sequence
41 - 49	1099.5770	1098.5697	1098.5709	-1	0	R.DYYLVSAIR.G
50 - 60	1005.5430	1004.5357	1004.5403	-5	0	R.GAGGGGLTLFR.G
61 - 79	2174.0350	2173.0277	2173.0433	-7	1	R.GRNELCPLDVVQLSSDSER.G
63 - 79	1960.9140	1959.9067	1959.9208	-7	0	R.NELCPLDVVQLSSDSER.G
91 - 103	1487.7610	1486.7537	1486.7627	-6	0	K.TSIINEDVDLNVR.F
110 - 117	1061.4830	1060.4757	1060.4760	-0	0	R.CNEPTVWR.V
128 - 145	1875.9430	1874.9357	1874.9527	-9	0	K.WFITTGGVEGNPGAQTLK.N
162 - 187	2971.2530	2970.2457	2970.2841	-13	0	K.IVHCPSVCESCVSLCNDVGVSNDHAR.R
196 - 207	1237.7230	1236.7157	1236.7190	-3	0	R.ALAVVLVPGNER.S

205.
Match to: **clementine0.9_027444m|PAC:19265462** Score: **67** Expect: **0.025**
Nominal mass (M_r): **35554**; Calculated pI value: **6.16**
NCBI BLAST search of clementine0.9_027444m|PAC:19265462 against nr
Unformatted sequence string for pasting into other applications

Fixed modifications: Carbamidomethyl (C)
Variable modifications: Oxidation (M)
Cleavage by Trypsin: cuts C-term side of KR unless next residue is P
Number of mass values matched: **9**
Sequence Coverage: **39%**

Matched peptides shown in **Bold Red**

1 MNGPEK**EVVC VTGANGFIGT WVVR**TLLEKG YTNIHAAIFP GTDASHLFSL
51 PGATKLNVR**V YEANILDNEA ISR**AIEGCKG VFHLASPNTL DDPKDPEKEL
101 LIPAVQGTLN VLEAAKKFGV RRVVLTSSIS SIVPNPNWPQ GK**VIDETSWT**
151 **DLDFCK**SHKI WYSMSK**TLAE KAAWEFAEK**N GTDVVAIHPA TSLGPFPQPY
201 VNASGAVLQR LLQGSK**DTQE HYWLGAVHVK** DVAK**AQVLLF ETSAASGR**YL
251 CTNGIYQFAE FAEKVSK**LFP EYPIHRFKGE TQPGLVACEN AAKRLISLGL**
301 **DFTPVEETIR** EAVESLKAQG HLG

Start - End	Observed	Mr(expt)	Mr(calc)	ppm	Miss	Sequence
7 - 24	1963.9600	1962.9527	1962.9986	-23	0	K.EVVCVTGANGFIGTWVVR.T
60 - 73	1606.7930	1605.7857	1605.7998	-9	0	R.VYEANILDNEAISR.A
143 - 156	1728.8000	1727.7927	1727.7713	12	0	K.VIDETSWTDLDFCK.S
167 - 179	1493.7230	1492.7157	1492.7561	-27	1	K.TLAEKAAWEFAEK.N
217 - 230	1682.8150	1681.8077	1681.8213	-8	0	K.DTQEHYWLGAVHVK.D

235 - 248	1449.7630	1448.7557	1448.7623	-5	0	K.AQVLLFETSAASGR.Y
268 - 276	1171.6230	1170.6157	1170.6186	-2	0	K.LFPEYPIHR.F
277 - 293	1819.8820	1818.8747	1818.8934	-10	1	R.FKGETQPGLVACENAAK.R
295 - 310	1802.9700	1801.9627	1801.9826	-11	0	R.LISLGLDFTPVEETIR.E

206.
Match to: **clementine0.9_012041m|PAC:19264672** Score: **97** Expect: **2.6e-005**
Nominal mass (M_r): **43638**; Calculated pI value: **7.70**
NCBI BLAST search of clementine0.9_012041m|PAC:19264672 against nr
Unformatted sequence string for pasting into other applications

Fixed modifications: Carbamidomethyl (C)
Variable modifications: Oxidation (M)
Cleavage by Trypsin: cuts C-term side of KR unless next residue is P
Number of mass values matched: **14**
Sequence Coverage: **51%**

Matched peptides shown in **Bold Red**

```
  1 MATLASSSSS LLLSSPPSKL AQASLPPSLR LSFSSYSHLS SLVSISPSSF
 51 LACPASSRRS SVSAFTVKAS AAEKKKVLIV NTNSGGHAVI GFYLAKELLG
101 SGHEVTIMTV GDENSDKMKK PPFNRFNEIV SAGGKTVWGD PAEVGNVVGG
151 VTFDVVLDNN GKNLDAVRPV ADWAKSSGVK QFLFISSAGI YKPADEPPHV
201 EGDVVKPDAG HVQVEKYISE NFSNWASFRP QYMIGSGNNK DCEEWFFDRI
251 VRKRPVPIPG SGMQFTNIAH VRDLSSMLTL AVENPEAASS NIFNLVSDRA
301 VTLDGMAKLC AQAAGLPVEI VHYDPKAAGI DAKKAFPFRN MHFYAEPRAA
351 KDILGWRSTT NLPEDLKERF EEYVKIGRDK KAMQFEIDDK ILESLKVPIP
401 V
```

Start - End	Observed	Mr(expt)	Mr(calc)	ppm	Miss	Sequence
60 - 74	1482.7580	1481.7507	1481.7726	-15	1	R.SSVSAFTVKASAAEK.K
77 - 96	2073.0830	2072.0757	2072.1419	-32	0	K.VLIVNTNSGGHAVIGFYLAK.E
163 - 175	1454.7780	1453.7707	1453.7677	2	0	K.NLDAVRPVADWAK.S
181 - 216	3904.0250	3903.0177	3902.9843	9	0	K.QFLFISSAGIYKPADEPPHVEGDVVKPDAGHVQVEK.Y
217 - 240	2826.3500	2825.3427	2825.2867	20	0	K.YISENFSNWASFRPQYMIGSGNNK.D Oxidation (M)
241 - 249	1303.5150	1302.5077	1302.4975	8	0	K.DCEEWFFDR.I
254 - 272	2093.1030	2092.0957	2092.1000	-2	0	K.RPVPIPGSGMQFTNIAHVR.D Oxidation (M)
273 - 299	2893.4430	2892.4357	2892.4175	6	0	R.DLSSMLTLAVENPEAASSNIFNLVSDR.A
273 - 299	2909.4510	2908.4437	2908.4124	11	0	R.DLSSMLTLAVENPEAASSNIFNLVSDR.A Oxidation (M)
309 - 326	1981.0300	1980.0227	1980.0139	4	0	K.LCAQAAGLPVEIVHYDPK.A
340 - 348	1164.5290	1163.5217	1163.5182	3	0	R.NMHFYAEPR.A
340 - 348	1180.5260	1179.5187	1179.5131	5	0	R.NMHFYAEPR.A Oxidation (M)
358 - 369	1402.7130	1401.7057	1401.7099	-3	1	R.STTNLPEDLKER.F
368 - 375	1099.5040	1098.4967	1098.5345	-34	1	K.ERFEEYVK.I

207.
Match to: **clementine0.9_008926m|PAC:19259027** Score: **188** Expect: **2.1e-014**
Nominal mass (M_r): **54000**; Calculated pI value: **6.00**
NCBI BLAST search of clementine0.9_008926m|PAC:19259027 against nr
Unformatted sequence string for pasting into other applications

Fixed modifications: Carbamidomethyl (C)
Variable modifications: Oxidation (M)
Cleavage by Trypsin: cuts C-term side of KR unless next residue is P
Number of mass values matched: **24**
Sequence Coverage: **57%**

Matched peptides shown in **Bold Red**

```
  1 MAPKALDYES MNENVKKVQY AVRGELYLRA SELQKEGKKI IFTNVGNPHA
 51 LGQRPLTFPR QVVALCQAPF LLDDPNVGIV FPADAIARAK HYLSLTSGGL
101 GAYSDSRGIP GVRKEVAEFI ERRDGYPSDP ELIFLTDGAS KGVMQTLNCV
151 IRGEGDGVLV PVPQYPLYSA TISLLGGSLV PYYLEETANW GLDLNDLQQS
201 VAQARSKGIT VRAMVIINPG NPTGQCLSEA NLREILRFCY QENLVLLGDE
251 VYQQNIYQDE RPFVSSKKVL MDMGPPFSKE VQLVSFHTVS KGYWGECGQR
301 GGYFEMTNIP PKTVDEIYKI ASIALSPNVP AQIFMGLMVN PLKPGDISYE
351 QFVRESKGIL ESLRRRARMM TDGFNSCRNV VCNFTEGAMY SFPQIRLPPK
401 AIEAAKRAGK VPDVFYCLRL LEATGISTVP GSGFGQKEGV FHLRTTILPA
451 EEDMPAIMES FKKFNDEFME QYEDYRGYSR M
```

Start - End	Observed	Mr(expt)	Mr(calc)	ppm	Miss	Sequence
61 - 88	3009.5190	3008.5117	3008.5794	-22	0	R.QVVALCQAPFLLDDPNVGIVFPADAIAR.A
89 - 107	1982.9740	1981.9667	1981.9857	-10	1	R.AKHYLSLTSGGLGAYSDSR.G
91 - 107	1783.8600	1782.8527	1782.8537	-1	0	K.HYLSLTSGGLGAYSDSR.G
114 - 122	1120.5790	1119.5717	1119.5924	-18	1	R.KEVAEFIER.R
115 - 122	992.5060	991.4987	991.4974	1	0	K.EVAEFIER.R
123 - 141	2080.9900	2079.9827	2080.0113	-14	1	R.RDGYPSDPELIFLTDGASK.G
124 - 141	1924.9040	1923.8967	1923.9102	-7	0	R.DGYPSDPELIFLTDGASK.G
213 - 233	2255.0970	2254.0897	2254.1198	-13	0	R.AMVIINPGNPTGQCLSEANLR.E
213 - 233	2271.0950	2270.0877	2270.1147	-12	0	R.AMVIINPGNPTGQCLSEANLR.E Oxidation (M)
238 - 267	3681.6980	3680.6907	3680.7457	-15	0	R.FCYQENLVLLGDEVYQQNIYQDERPFVSSK.K
280 - 291	1373.7130	1372.7057	1372.7351	-21	0	K.EVQLVSFHTVSK.G
292 - 300	1112.4540	1111.4467	1111.4505	-3	0	K.GYWGECGQR.G
301 - 312	1353.6520	1352.6447	1352.6435	1	0	R.GGYFEMTNIPPK.T
301 - 312	1369.6450	1368.6377	1368.6384	-0	0	R.GGYFEMTNIPPK.T Oxidation (M)
320 - 354	3847.9300	3846.9227	3847.0052	-21	0	K.IASIALSPNVPAQIFMGLMVNPLKPGDISYEQFVR.E 2 Oxidation (M)
379 - 396	2132.9790	2131.9717	2131.9819	-5	0	R.NVVCNFTEGAMYSFPQIR.L
379 - 396	2148.9710	2147.9637	2147.9769	-6	0	R.NVVCNFTEGAMYSFPQIR.L Oxidation (M)
411 - 419	1168.5810	1167.5737	1167.5747	-1	0	K.VPDVFYCLR.L

420 - 437	1761.9150	1760.9077	1760.9309	-13	0	**R.LLEATGISTVPGSGFGQK.E**
438 - 444	857.4430	856.4357	856.4555	-23	0	**K.EGVFHLR.T**
445 - 462	2054.9580	2053.9507	2053.9588	-4	0	**R.TTILPAEEDMPAIMESFK.K** 2 Oxidation (M)
463 - 476	1929.8160	1928.8087	1928.7887	10	1	**K.KFNDEFMEQYEDYR.G** Oxidation (M)
464 - 476	1785.7260	1784.7187	1784.6988	11	0	**K.FNDEFMEQYEDYR.G**
464 - 476	1801.6950	1800.6877	1800.6937	-3	0	**K.FNDEFMEQYEDYR.G** Oxidation (M)

208.
Match to: **clementine0.9_008884m|PAC:19278695** Score: **106** Expect: **3.4e-006**
Nominal mass (M_r): **53950**; Calculated pI value: **6.19**
NCBI BLAST search of clementine0.9_008884m|PAC:19278695 against nr
Unformatted sequence string for pasting into other applications

Fixed modifications: Carbamidomethyl (C)
Variable modifications: Oxidation (M)
Cleavage by Trypsin: cuts C-term side of KR unless next residue is P
Number of mass values matched: **17**
Sequence Coverage: **41%**

Matched peptides shown in **Bold Red**

```
  1 MSCREGLMSP QTETKASVGF KAGVKDYKLT YDTPDYVTKD TDILAAFRVT
 51 PQPGVPPEEA GAAVAAESST GTWTAVWTDG LTSLDRYKGR CYNIEPVAGE
101 ENQYICYVAY PLDLFEEGSV TNMFTSIVGN VFGFKALRAL RLEDLRIPPA
151 YTKTFQGPPH GIQVERDKLN KYGRPLLGCT IKPKLGLFAK NYGRAVYECL
201 RGGLDFTKDD ENVNSQPFMR WRDRFLFCAE ALYKAQAETG EIKGHYLNAT
251 AGTCEEMLKR AVFARELGVP IVMHDYLTGG FTANTTLAHY CRDNGLLLHI
301 HRAMHAVIDR QKNHGMHFRV LAKALRLSGG DHIAHGTVIG KLEGERDITL
351 GFVDLLRDDF VEKDRSRGIY FTQDWVSIPG VIPVAFGGIH VWHMPALTEI
401 FGDDSVLQFG GGTLGHPWGN ALGAVANRVS LEACVQARNE GRDLAREGNE
451 IIREASKWSP ELAAACEVWK SIKFEFAAMD TL
```

Start - End	Observed	Mr(expt)	Mr(calc)	ppm	Miss	Sequence
5 - 21	1825.8210	1824.8137	1824.8928	-43	1	**R.EGLMSPQTETKASVGFK.A** Oxidation (M)
40 - 48	1021.5250	1020.5177	1020.5240	-6	0	**K.DTDILAAFR.V**
49 - 86	3824.8080	3823.8007	3823.8541	-14	0	**R.VTPQPGVPPEEAGAAVAAESSTGTWTAVWTDGLTSLDR.Y**
154 - 166	1465.7500	1464.7427	1464.7474	-3	0	**K.TFQGPPHGIQVER.D**
172 - 184	1502.8300	1501.8227	1501.8439	-14	0	**K.YGRPLLGCTIKPK.L**
195 - 201	910.4440	909.4367	909.4378	-1	0	**R.AVYECLR.G**
202 - 220	2169.9710	2168.9637	2168.9797	-7	1	**R.GGLDFTKDDENVNSQPFMR.W**
202 - 220	2185.9720	2184.9647	2184.9746	-5	1	**R.GGLDFTKDDENVNSQPFMR.W** Oxidation (M)
209 - 220	1451.6470	1450.6397	1450.6147	17	0	**K.DDENVNSQPFMR.W**
244 - 259	1810.8180	1809.8107	1809.8026	5	0	**K.GHYLNATAGTCEEMLK.R** Oxidation (M)
244 - 260	1966.9270	1965.9197	1965.9037	8	1	**K.GHYLNATAGTCEEMLKR.A** Oxidation (M)
266 - 292	3036.4110	3035.4037	3035.4634	-20	0	**R.ELGVPIVMHDYLTGGFTANTTLAHYCR.D**

266 - 292	3052.4350	3051.4277	3051.4583	-10	0	R.ELGVPIVMHDYLTGGFTANTTLAHYCR.D	Oxidation (M)
293 - 302	1187.6580	1186.6507	1186.6571	-5	0	R.DNGLLLHIHR.A	
313 - 319	898.4080	897.4007	897.4028	-2	0	K.NHGMHFR.V	
347 - 357	1261.7100	1260.7027	1260.7078	-4	0	R.DITLGFVDLLR.D	
458 - 470	1546.7350	1545.7277	1545.7286	-1	0	K.WSPELAAACEVWK.S	

209.
Match to: **clementine0.9_008455m|PAC:19269731** Score: **110** Expect: **1.3e-006**
Nominal mass (M_r): **57669**; Calculated pI value: **6.64**
NCBI BLAST search of clementine0.9_008455m|PAC:19269731 against nr
Unformatted sequence string for pasting into other applications

Fixed modifications: Carbamidomethyl (C)
Variable modifications: Oxidation (M)
Cleavage by Trypsin: cuts C-term side of KR unless next residue is P
Number of mass values matched: **13**
Sequence Coverage: **35%**

Matched peptides shown in **Bold Red**

```
  1 MDPYKLLPST THNAPFMTTN AGAPVWNDNH SLTVGSRGPV LLEDYHLVEK
 51 LAHFARERIP ERVVHARGAS AKGFFECTHD ISHLTCADLF RAPGVQTPVI
101 VRFSTVIHER GSPETLRDPR GFAVKFYTRE GNWDLLGNSI PVFFIRDAIK
151 FPDVIHAFKP NPKSHIQEYW RILDFCSHLP ESLSTFSWFF DDVGIPQDYR
201 HMEGFGVQTF TLVNKNGKVH YVKFHWKPTC GVKCLIDDEE AVKVGGSNHS
251 HATQDLYDSI AAGNYPEWKL YIQTIDPDHE DQFDFDPLDV TKWWPEDIIP
301 LQPVGRLVLN KNIDNFFAEN EMLAFNPGIV VPGIYYSNDK MLQCRIFAYG
351 DTQRHRLGPN YLMLPVNAPK CPHRNNHYDG FMNFMHRDEE VDYFPSRYDP
401 TRHAERYPIP SDVITGRREK ACIEKENNFK QPGERYRSWA PDRQERFLNR
451 WIKALCDPRV THEVRSIWVS YWSQADRSLG QKLASRLNVR PSI
```

Start - End	Observed	Mr(expt)	Mr(calc)	ppm	Miss	Sequence	
73 - 91	2326.0540	2325.0467	2325.0307	7	0	K.GFFECTHDISHLTCADLFR.A	
92 - 102	1136.6710	1135.6637	1135.6714	-7	0	R.APGVQTPVIVR.F	
103 - 110	988.5200	987.5127	987.5138	-1	0	R.FSTVIHER.G	
111 - 120	1127.5700	1126.5627	1126.5731	-9	1	R.GSPETLRDPR.G	
164 - 171	1118.5390	1117.5317	1117.5305	1	0	K.SHIQEYWR.I	
244 - 269	2817.2620	2816.2547	2816.2790	-9	0	K.VGGSNHSHATQDLYDSIAAGNYPEWK.L	
270 - 292	2764.2620	2763.2547	2763.2916	-13	0	K.LYIQTIDPDHEDQFDFDPLDVTK.W	
293 - 306	1705.8580	1704.8507	1704.8988	-28	0	K.WWPEDIIPLQPVGR.L	
346 - 354	1070.5220	1069.5147	1069.5193	-4	0	R.IFAYGDTQR.H	
375 - 387	1714.7410	1713.7337	1713.6776	33	0	R.NNHYDGFMNFMHR.D	2 Oxidation (M)
388 - 397	1256.5490	1255.5417	1255.5357	5	0	R.DEEVDYFPSR.Y	
407 - 417	1217.6480	1216.6407	1216.6452	-4	0	R.YPIPSDVITGR.R	
466 - 477	1497.7120	1496.7047	1496.7048	-0	0	R.SIWVSYWSQADR.S	

211.
Match to: **clementine0.9_023186m|PAC:19268625** Score: **92** Expect: **8.1e-005**
Nominal mass (M_r): **20521**; Calculated pI value: **9.16**
NCBI BLAST search of clementine0.9_023186m|PAC:19268625 against nr
Unformatted sequence string for pasting into other applications

Fixed modifications: Carbamidomethyl (C)
Variable modifications: Oxidation (M)
Cleavage by Trypsin: cuts C-term side of KR unless next residue is P
Number of mass values matched: **8**
Sequence Coverage: **41%**

Matched peptides shown in **Bold Red**

```
  1 MASSMISSAT VATANRASLA QASMVAPFTG LKSSSAFPAT KKTNNDITSI
 51 ASNGGRVQCM KVWPPTGLKK FETLSYLPPL SDEALLKEIS YLIRSGWIPC
101 LEFELEKGWV YREHHRSPGY YDGRYWTMWK LPMYGCTDAT QVLKEVGEVQ
151 KEYPHSFVRI IGFDNKRQVQ CISFIAAKPP GV
```

Start - End	Observed	Mr(expt)	Mr(calc)	ppm	Miss	Sequence
1 - 16	1629.7470	1628.7397	1628.7498	-6	0	-.MASSMISSATVATANR.A 2 Oxidation (M)
70 - 87	2064.1060	2063.0987	2063.1190	-10	1	K.KFETLSYLPPLSDEALLK.E
71 - 87	1936.0210	1935.0137	1935.0241	-5	0	K.FETLSYLPPLSDEALLK.E
88 - 94	893.5110	892.5037	892.5018	2	0	K.EISYLIR.S
95 - 107	1607.7650	1606.7577	1606.7701	-8	0	R.SGWIPCLEFELEK.G
131 - 144	1596.7640	1595.7567	1595.7688	-8	0	K.LPMYGCTDATQVLK.E
131 - 144	1612.7540	1611.7467	1611.7637	-11	0	K.LPMYGCTDATQVLK.E Oxidation (M)
152 - 159	1034.5050	1033.4977	1033.4981	-0	0	K.EYPHSFVR.I

214.
Match to: **orange1.1g025928m|PAC:18110705** Score: **48** Expect: **1.9**
Nominal mass (M_r): **26777**; Calculated pI value: **8.63**
NCBI BLAST search of orange1.1g025928m|PAC:18110705 against nr
Unformatted sequence string for pasting into other applications

Fixed modifications: Carbamidomethyl (C)
Variable modifications: Oxidation (M)
Cleavage by Trypsin: cuts C-term side of KR unless next residue is P
Number of mass values matched: **6**
Sequence Coverage: **32%**

Matched peptides shown in **Bold Red**

```
  1 MASTQCFLHH HALSTTPART SSSQRHVSNI KPTQIVCRAQ KQAVQEDDGS
```

51 AVSRRLALTV LIGAAAVGSK VSPADAAYGE SANVFGKPKT NTDFLPYNGD
101 GFKLSIPSKW NPSK**EREFPG QVLRYEDNFD SNSNVSVIIT PTDKKSITDY**
151 **GSPEEFLSK**V DYLLGKQAYS GK**TSSEGGFD PDAVATANIL EASVRPPYYF**
201 **LSVLTR**TADG DEGGKLYICK AQAGDKRWFK GTRKYVESTA SSFSVA

Start - End	Observed	Mr(expt)	Mr(calc)	ppm	Miss	Sequence
115 - 124	1230.6770	1229.6697	1229.6517	15	1	K.EREFPGQVLR.Y
117 - 124	945.5240	944.5167	944.5080	9	0	R.EFPGQVLR.Y
125 - 144	2258.0780	2257.0707	2257.0386	14	0	R.YEDNFDSNSNVSVIITPTDK.K
125 - 145	2386.1630	2385.1557	2385.1336	9	1	R.YEDNFDSNSNVSVIITPTDKK.S
146 - 159	1572.7640	1571.7567	1571.7355	14	0	K.SITDYGSPEEFLSK.V
173 - 206	3643.8570	3642.8497	3642.8206	8	0	K.TSSEGGFDPDAVATANILEASVRPPYYFLSVLTR.T
